# Single-cell sequencing-guided discovery of narciclasine as a dual VCAM-1/ICAM-1 inhibitor for atherosclerosis management

**DOI:** 10.1016/j.gendis.2026.102167

**Published:** 2026-03-27

**Authors:** Yang Wang, Zhen Cao, Huai Lan, Bo Li, Guixue Wang, Qingmei Chen

**Affiliations:** a College of Environment and Ecology, Chongqing University, Chongqing 400044, China; b Department of Cardiology, Chongqing University Jiangjin Hospital, Jiangjin Central Hospital of Chongqing, Chongqing 402260, China; c Key Laboratory for Biorheological Science and Technology of Ministry of Education, National Local Joint Engineering Laboratory for Vascular Implants, Bioengineering College of Chongqing University, Chongqing 400044, China; d Jinfeng Laboratory, Chongqing 401329, China

**Keywords:** Atherosclerosis, Machine learning, Narciclasine, Network pharmacology, Single-cell sequencing, VCAM-1/ICAM-1

## Abstract

Atherosclerosis (AS) remains a leading cause of cardiovascular morbidity and mortality worldwide, with current treatments focused primarily on reducing low-density lipoprotein levels while failing to repair damaged endothelial cells, thus highlighting the urgent need for novel therapeutic drugs. To address this challenge, we analyzed human AS patient single-cell RNA sequencing datasets to identify disease-driving genes and then employed connectivity map analysis to screen for potential therapeutic compounds. Using network pharmacology and machine learning to predict core drug targets, the study validated drug-target interactions through molecular docking, molecular dynamics simulations, and surface plasmon resonance analysis, with experimental validation conducted using endothelial cell damage models and *ApoE*^*−/−*^ atherosclerotic mice. The integrative approach successfully identified narciclasine as a promising therapeutic compound that targets vascular cell adhesion molecule 1 and intercellular adhesion molecule-1 (VCAM-1/ICAM-1) for AS treatment, with molecular studies confirming strong binding affinity and experimental validation demonstrating significant alleviation of endothelial dysfunction through downregulation of VCAM-1/ICAM-1 expression and reduction of aortic plaque burden in mouse models. This multiplatform methodology combining single-cell sequencing, network pharmacology, machine learning, computational simulation, and experimental validation provides a robust framework for drug discovery while positioning narciclasine as a promising therapeutic candidate warranting clinical investigation for AS treatment.

## Introduction

Atherosclerosis (AS) is a primary pathological cause of various cardiovascular conditions, including coronary artery disease, stable angina, and ischemic heart disease.^1^ The incidence of AS has steadily increased, making it the leading cause of death globally and responsible for approximately 17.6 million deaths annually, thereby posing a significant threat to public health.^2^ The pathogenesis of AS is a complex, dynamic process that primarily affects large- and medium-sized arteries.^3^^,^^4^ In its early stages, endothelial cells recruit monocytes to the arterial wall through chemokine receptor interactions and increase the expression of intercellular adhesion molecules (ICAM-1) and vascular cell adhesion molecules (VCAM-1).^5^, ^6^, ^7^ Simultaneously, increased cholesterol transport activity leads to significant cholesterol uptake by M1 macrophages, resulting in lipid deposition and the formation of heterogeneous atherosclerotic plaques.^8^ Inflammation renders these plaques unstable, characterized by thin fibrous caps, enlarged necrotic cores, and increased susceptibility to rupture, exposing pro-thrombotic material and triggering thrombosis and arterial occlusion.^8^^,^^9^ Thus, the progression of AS is closely associated with lipid deposition and chronic inflammation.^8^, ^9^, ^10^

Currently, lipid-lowering drugs (including statins) are the standard treatment for AS.^11^, ^12^, ^13^ Although statin therapy has been shown to reduce the risk of AS-related events, long-term use may lead to liver enzyme abnormalities, muscle toxicity, and diabetes mellitus, with poor patient compliance, and lipid-lowering drugs are unable to repair damaged endothelial cells.^13^^,^^14^ Therefore, given the shortcomings of current first-line clinical drugs, the development of safe and effective new anti-AS drugs is particularly necessary.

In recent decades, researchers have endeavored to search for potential anti-atherosclerotic agents among natural compounds through genomics.^15^ Single-cell RNA sequencing (scRNA-seq) has emerged as a powerful tool for revealing the gene expression heterogeneity involved in the development and progression of AS at the single-cell level, providing new insights into its pathological progression.^16^, ^17^, ^18^ Additionally, the connectivity map (CMAP), a comprehensive database and associated software, contains whole-genome gene expression profiles derived from human cell lines treated with more than a thousand small molecules.^19^ CMAP allows comparison of gene expression patterns between two datasets to identify small molecules that induce expression patterns similar to those of the target genes.^20^ This database has proven to be an effective tool for predicting the pharmacological functions and mechanisms of action in drug discovery. For example, CMAPs were used to identify celastrol, a small molecule that mimics obesity-related gene expression profiles, and have shown promise in treating obesity.^21^

This study aims to systematically analyze scRNA-seq data from human atherosclerotic plaques and adjacent normal tissues to guide the identification of potential drugs for AS treatment. Narciclasine was identified as a promising candidate by integrating human AS scRNA-seq data with CMAPs. Narciclasine, a compound derived from the bulbs of various *Narcissus* species, has a wide range of bioactivities, including anti-tumor, anti-inflammatory, anti-Alzheimer’s and anti-obesity effects.^22^ Through network pharmacology and machine learning, VCAM-1 and ICAM-1 have been identified as crucial factors in the development of AS and as potential core targets of narciclasine for AS treatment. These findings were further validated via molecular docking, molecular dynamics (MD) simulations (MDS), and surface plasmon resonance (SPR) analysis. In addition, narciclasine was shown to alleviate vascular damage and reduce atherosclerotic plaque formation by downregulating VCAM-1 and ICAM-1. This study integrates multiple scRNA-seq data, network pharmacology, machine learning, and computational simulations, offering new insights for drug development and the therapeutic potential of narciclasine in AS treatment.

## Materials and methods

### Analysis of AS scRNA-seq datas

Two publicly available scRNA-seq datasets, GSE155512 and GSE159677, from the GEO database (www.ncbi.nlm.nih.gov/geo) were analyzed. GSE155512 included 8867 scRNA-seq entries from three human AS carotid artery plaque tissues,^23^ whereas GSE159677 included 51,981 scRNA-seq entries from three human AS carotid artery plaques and adjacent tissues.^24^ The scRNA-seq expression matrixes were processed via the Seurat package (v4.2.0) within the R (v4.3.2) environment. The cell selection criteria included the exclusion of cells expressing fewer than 200 or more than 4000 genes. The “FeatureScatter” function was used to evaluate the percentage of mitochondrial genes and unique genes in the total number of molecules, and those with > 10% mitochondrial RNA content cell were removed (Fig. S1A). The Scater package (version 3.11) was used for further data standardization and deconvolution-based normalization. To eliminate batch effects between samples, we used the “Harmony” function for data integration. Next, 2000 highly variable genes in cells were identified for clustering. The optimal number of principal components (PCs) was determined via the “ElbowPlot” function, and dimensionality was reduced to 30 PCs via the “RunPCA” function (Fig. S1B). The feature genes were then clustered via the “FindNeighbors” and “FindClusters” functions, with a resolution of 0.5. Seurat was further employed to assign cell types to each cluster, with manual annotation on the basis of known marker genes from published datasets. Cell types were manually annotated on the basis of marker genes from published datasets, The key markers for each cell type are as follows: mast cell (Mast): tryptase alpha/beta 1 (TPSAB1)/tryptase beta 2 (TPSB2)/membrane spanning 4-domains A2 (MS4A2); B-cell (B): immunoglobulin kappa constant (IGKC)/membrane spanning 4-domains A1 (MS4A1)/CD79A; T-cell (T): CD69/T-cell receptor alpha constant (TRAC)/CD2; natural killer T-cell (NKT): cathepsin W (CTSW)/X–C motif chemokine ligand 1(XCL1)/natural killer cell granule protein 7 (NKG7); vascular smooth muscle cell (VSMCs): transgelin (TAGLN)/myosin light chain 9 (MYL9)/caldesmon 1 (CALD1); macrophages: CD68/CD14/ALF transcription elongation factor 1 (ALF1); and endothelial cells (VECs): endothelial cell surface expressed chemotaxis and apoptosis regulator (ECSCR)/platelet and endothelial cell adhesion molecule 1 (PECAM1)/Von Willebrand factor (VWF).

### Cell communication analysis

Cell–cell communication across different cell types in AS plaques and adjacent tissues was analyzed via the “CellChat” package, with the results visualized via the “Patchwork” package. Ligand‒receptor interactions were identified through the “CellChat DB.human”. The “computecommunication” function was used to assess the interaction probabilities between cells, whereas the “filtercommunication” function excluded interactions observed in fewer than 10 cells. Additionally, the “computeCommunProbPathway” function was used to calculate communication probabilities at the signaling pathway level, and the “subsetCommunication” function was employed to construct the cell–cell communication network.

### Identification of genes associated with AS plaques

Differentially expressed genes (DEGs) were identified on the basis of the log-fold change and minimum gene expression percentage between the same cell types in AS plaques and adjacent regions. The minimum expression threshold was set at 0.25, with an adjusted *P* value < 0.05. DEGs were determined via the “FindMarkers” function. DEG enrichment analysis was conducted via the Kyoto Encyclopedia of Genes and Genomes (KEGG) signaling pathways and Gene Ontology (GO) from the Metascape database (www.Metascape.org). Genes highly expressed in various cell types associated with AS plaque regions intersected, and genes upregulated in at least two cell types were considered key genes for plaque formation. Protein–protein interactions (PPIs) of key genes were analyzed via the STRING database (www.string-db.org).

### Discovery of potential therapeutic drugs for AS

Potential therapeutic drugs for AS were screened on the basis of the DEGs in various cell types within atherosclerotic plaque regions compared with those in adjacent regions (at least those differentially expressed in two types of cells). Drug targets were identified through the Binding DB database (www.bindingdb.org), PubChem database (https://pubchem.ncbi.nlm.nih.gov), STITCH database (http://stitch.embl.de), TargetNet database (http://targetnet.scbdd.com), and SwissTarget database (http://swisstargetprediction.ch). GO and KEGG signaling pathway enrichment analyses were performed via the ClueGO plugin in Cytoscape software (v3.7.2). The molecular complex detection (MCODE) plugin in Cytoscape was used to screen modules of the PPI network. The default settings for module inference were degree cutoff = 2, node score cutoff = 0.2, K-core = 2, and max depth = 100.

### Network pharmacology analysis

AS disease targets were collected from the DrugBank database (https://go.drugbank.com/drugs), the DisGenet database (www.disgenet.org), the GeneCards database (www.genards.org), the OMIM database (www.omim.org), and the TDD database (https://db.idrblab.net/ttd). Targets identified in at least three databases were selected as AS-related targets. The intersection of narciclasine targets, AS DEGs, and AS targets was determined, and KEGG signaling pathway enrichment and protein–protein interaction (PPI) analyses were performed on these intersecting genes. The relationships were visualized via Cytoscape software, and the core degree was displayed according to the visualization size.

### Core target selection via machine learning

We constructed a machine learning model based on the intersection gene expression matrix of GSE100927 from the GEO dataset, which contains transcriptomic data from 104 human peripheral artery samples, including those from the carotid, femoral, and subpopliteal regions of both atherosclerotic and control tissues. We compared the performance of four machine learning algorithms—random forest (RF), support vector machine (SVM), generalized linear model (GLM), and extreme gradient boosting (XGB)—using residual analysis and receiver operating characteristic (ROC) curves to identify core targets related to narciclasine in AS treatment. The “mlbench” and “caret” R packages were employed for SVM-RFE, with all the data normalized before model training. This integrated approach facilitated the identification of robust biomarkers with high discriminatory power, and the top 10 variables were selected as hub genes.

### Molecular docking

The crystal structures of VCAM-1 (ID: 1VSC) and ICAM-1 (ID: 5E6D) were downloaded from the protein data bank database (www.rcsb.org) for molecular docking. Unwanted ions and water molecules were eliminated from the crystal structure during protein preparation. The prepared structure was energy minimized and utilized for the subsequent computational screening and validation process. The structure of narciclasine was obtained from PubChem. Molecular docking of the drug ligand and protein 3D structures was performed via AutoDock Vina (v1.5.6). The Surflex-Dock scoring function considers hydrophobic, polar, repulsive, entropy, and solvation terms. The conformation with the highest score, on the basis of this function, was selected as the final bioactive conformation. Conformations with docking scores > 7 were considered potential drug targets. The active pockets and amino acid sites of the drug-target protein were visualized via PyMol (https://pymol.org).

### MDS analysis

MDS was conducted to evaluate the time-dependent behavior and conformational changes of the complexes, offering valuable insights into their structure, dynamics, and thermodynamics. MDS was performed via GROMACS 2022. The receptor protein was parameterized with the CHARMM force field, whereas the ligand parameters were derived from the CGenFF force field.^25^ The topology files for the system were generated via the pdb2gmx module and the AutoFF web server. The solvated system was embedded in a cubic box of TIP3P water molecules, maintaining a 1 nm buffer distance from the solute surface.^26^ Ions were added to neutralize the system charge via the gmx generation tool. Long-range electrostatic interactions were calculated via the particle mesh Ewald (PME) method with a 1 nm cutoff, and bond constraints were enforced with the SHAKE algorithm. The Verlet leapfrog integrator was employed with a 1 fs time step.

Energy minimization was performed in three phases: (1) 3000 steps of steepest descent optimization with constrained solute atoms and relaxed solvent molecules, (2) 2000 steps of conjugate gradient minimization to equilibrate counterions under positional constraints, and (3) full system optimization without restraints to achieve energy convergence. Production simulations were conducted under NPT ensemble conditions (310 K, 1 bar) for 50 ns. The trajectory data were analyzed via standard GROMACS: the root mean square deviation (RMSD) and residue-specific root mean square fluctuation (RMSF) were computed via gmx rmsd and gmx rmsf, respectively. Hydrogen bond occupancy was quantified via gmx hbond, and the radius of gyration (Rg) and solvent-accessible surface area (SASA) were determined via gmx gyrate and gmx sasa.

### Mathematical modeling of the synergistic dual-target inhibition of VCAM-1/ICAM-1

To quantitatively assess the synergistic inhibitory effect of narciclasine on VCAM-1 and ICAM-1, a mathematical modeling approach was employed. These integrated target-specific pharmacodynamic response curves and the Bliss independence model were used for synergy analysis. The inhibition of each molecular target by narciclasine was described via the classical Hill equation, a widely applied sigmoidal model in receptor–ligand interactions and drug-response modeling^27^:

$$E_{i}\left(C\right)=\frac{Cn}{IC_{50,i}^{n}+C^{n}}$$

where *E*_*i*_*(C)* denotes the inhibition efficacy of target *i* (either VCAM-1 or ICAM-1), *C* is the concentration of narciclasine, *IC*_*50,i*_ is the half-maximal inhibitory concentration, and *n* is the Hill coefficient, reflecting the steepness of the dose‒response relationship. For this model, the IC_*50,*__*VCAM-1*_ concentration and IC_5*0 of ICAM-1*_ were 5 μM and 3 μM, respectively, with a Hill coefficient of *n* = 2, indicating moderate cooperative inhibition.

The Bliss independence model was used to evaluate the combined inhibitory effect of dual-target engagement.^28^ This model assumes probabilistic independence between the effects of two agents or pathways:

E_combo_ (C) = E_1_(C) + E_2_(C) − E_1_(C) ∗ E_2_(C)

where *E1(C)* and *E2(C)* represent the individual inhibition efficacies of VCAM-1 and ICAM-1 at concentration *C*, respectively, and *E*_*combo*_*(C)* denotes the combined inhibition outcome. This framework is widely used for synergy analysis in studies of cancer, inflammation, and infectious diseases.^29^

The above models were implemented in both Python 3.9 (NumPy and Matplotlib) and MATLAB R2023b. Narciclasine concentrations ranging from 0.01 μM to 20 μM were evaluated at 500 evenly spaced intervals. The inhibition curves for each target and the calculated Bliss-combined curve were plotted to visualize the synergistic interaction.

### SPR analysis

SPR measurements were conducted via a Biacore T200 system (Cytiva) at 25 °C. Recombinant human VCAM-1 and ICAM-1 proteins (P19320-1, P05362, R&D Systems, MN, USA) were immobilized on separate flow cells of a CM5 sensor chip via standard amine coupling chemistry. A mixture of 0.4 M 1-ethyl-3-(3-dimethylaminopropyl) carbodiimide (EDC) and 0.1 M N-hydroxysuccinimide (NHS) was used to activate the carboxymethylated dextran surface. Protein solutions (10 μg/mL in 10 mM sodium acetate buffer, pH 4.5) were injected over the activated surface until approximately 5000 response units (RU) for VCAM-1 and 4800 RU for ICAM-1 were reached. Blocking was achieved with 1 M ethanolamine-HCl (pH 8.5). All binding experiments were conducted in HBS-EP + running buffer (10 mM HEPES, 150 mM NaCl, 3 mM EDTA, 0.05% surfactant P20, pH 7.4) supplemented with 5% DMSO to ensure the solubility of narciclasine (HY-16563, MCE; New Jersey, USA). Narciclasine was serially diluted in running buffer from 0.15 to 10 μM and injected over the immobilized proteins. The data were fitted to a 1:1 Langmuir binding model via Biacore T200 evaluation software (version 3.2), and the KD, kon, and koff values were determined.

### Cell culture

Human umbilical vein endothelial cells (HUVECs) were obtained from ATCC (Manassas, VA, USA) and cultured in Dulbecco’s modified Eagle’s medium (DMEM) (Gibco, Grand Island, NY, USA) supplemented with 10% fetal bovine serum (FBS) (Gibco, Grand Island, NY, USA) under 5% CO_2_ at 37 °C.

### Tube formation assay

For the *in vitro* angiogenesis assay, Matrigel (Becton Dickinson, MA, USA) was polymerized in 24-well plates at 37 °C for 30 min. Following polymerization, HUVECs (10^4^ cells/well) were seeded onto Matrigel. To establish an AS model, cells were treated with 50 mg/ml oxidized low-density lipoprotein (ox-LDL) (Union-Bio Technology, Shanghai, China). The treatment group was further exposed to 0.1 μmol/L narciclasine (HY-16563, MCE; New Jersey, USA), whereas the control group was left untreated with ox-LDL. Cells at passages 4–6 were harvested for subsequent analyses.

### RNA extraction and qRT‒PCR analysis of ICAM-1/VCAM-1 *in vitro*

Total RNA was extracted via TRIzol reagent (Takara, Tokyo, Japan), and RNA concentrations were measured via a Nanodrop ND-1000 spectrophotometer (Thermo Fisher Scientific, Inc., MA, USA). cDNA synthesis was performed with the PrimeScript RT reagent Kit (Takara, Tokyo, Japan), following the reaction conditions of 37 °C for 15 min and 85 °C for 5 min qRT‒PCR was carried out using SYBR Premix Ex Taq (Takara, Tokyo, Japan) on a CFX Connect Real-Time System (Bio-Rad, CA, USA). The following cycle conditions were used: 95 °C for 30 s; 40 cycles of 95 °C for 3 s, 55 °C for 30 s, and 72 °C for 30 s; and 95 °C for 1 min. Data analysis was performed via the 2(^–ΔΔCt^) method.^30^ The primer sequences are listed in Supplementary Table 1.

### Immunofluorescence for ICAM-1/VCAM-1 *in vitro*

To investigate the effects of narciclasine on ICAM-1/VCAM-1 protein expression, HUVECs were seeded and treated with ox-LDL or ox-LDL + narciclasine overnight. After fixation with 4% paraformaldehyde, the cells were stained for ICAM-1/VCAM-1. Primary antibodies against ICAM-1 (1:200, Proteintech, Wuhan, China) and VCAM-1 (1:200, R&D Systems, MN, USA) were used, with goat anti-mouse IgG H&L (Alexa Fluor®555, 1:200, Proteintech, Wuhan, China) and donkey anti-rabbit IgG H&L (Alexa Fluor®647, 1:200, Proteintech, Wuhan, China) as secondary antibodies. Images were captured using a Leica SP8 microscope.

### Animal experiments and groups

Six-week-old male *ApoE*^*−/−*^ mice (22 ± 1 g) were obtained from Beijing Vital River Laboratory Animal Technology (Beijing, China) and housed under specific pathogen-free conditions (26 °C, 40%–70% humidity, 45 Pa pressure, and a 12 h/12 h light/dark cycle). After 1 week of acclimatization, the mice were randomly divided into three groups: a control group, a PBS group, and a narciclasine intervention group, with eight mice in each group. The PBS and narciclasine intervention groups were fed a high-fat diet for 12 weeks and the control group received a normal diet until sacrifice. The PBS group received 200 μL of PBS via gavage, and the narciclasine group received 200 μL of narciclasine solution (2 mg/kg^31^ in deionized water) from the eighth week onward. Gavage was administered daily for 4 weeks. Following the intervention, the mice were anesthetized and sacrificed. Aortic tissues were stored in 4% formalin or at −80 °C for histopathological analysis. The animal study was approved by the Ethics Committee of Chongqing University (Approval Code: A-2019-021) and adhered to NIH guidelines for the care and use of laboratory animals.

### Oil red O staining of whole aortas after *in vivo* narciclasine therapy

To evaluate the deposition of AS plaques, whole aortas from the three groups were collected and fixed with 4% paraformaldehyde at the indicated time points and then stained with Oil Red O staining reagent, according to the manufacturer’s protocols (G1015-100 ML, ServiceBio, Wuhan, China). Images were taken after staining with an Olympus microscope.

### Histological analysis after *in vivo* narciclasine therapy

Aortic tissues were fixed in 4% paraformaldehyde, and the aortic roots were embedded in paraffin and sectioned at 5 μm thickness. The sections were stained with hematoxylin-eosin (HE) and Oil Red O (G1015-100 ML, ServiceBio, Wuhan, China) according to standard protocols. Images of the stained sections were acquired via an Olympus microscope, scanned with CaseViewer, and analyzed via ImageJ.

### Immunohistochemical analysis of ICAM-1/VCAM-1 *in vivo* after narciclasine therapy

To evaluate the ability of narciclasine to affect ICAM-1/VCAM-1 expression *in vivo*, primary antibodies against ICAM-1 (1:500, ServiceBio, Wuhan, China) and VCAM-1 (1:500, ServiceBio, Wuhan, China) were applied. The secondary antibodies were chosen on the basis of the species of the primary antibodies. After staining, the samples were viewed via an Olympus microscope, scanned via Case Viewer, and analyzed via ImageJ.

### RNA extraction and qRT‒PCR analysis of ICAM-1/VCAM-1 *in vivo*

Total RNA was extracted from the aortic roots of the three experimental groups, measured, reverse-transcribed into cDNA, and subjected to qRT‒PCR as described above. Data analysis was performed via the 2^−ΔΔCt^ method. The primer sequences for mouse β-actin/ICAM-1/VCAM-1 are also listed in Supplementary Table 1.

### Statistical analysis

The data were analyzed as the means ± SD via GraphPad 8 Prism software (GraphPad, San Diego, CA, USA). All experiments were repeated at least three times. Unpaired two-sided Student’s *t* test or one-way analysis of variance (ANOVA) was used to calculate *P* values. Statistical significance was defined as *∗p* < 0.05.

## Results

### Identification of the scRNA-seq cell subpopulations in AS plaques and adjacent regions

**Figure 1:**
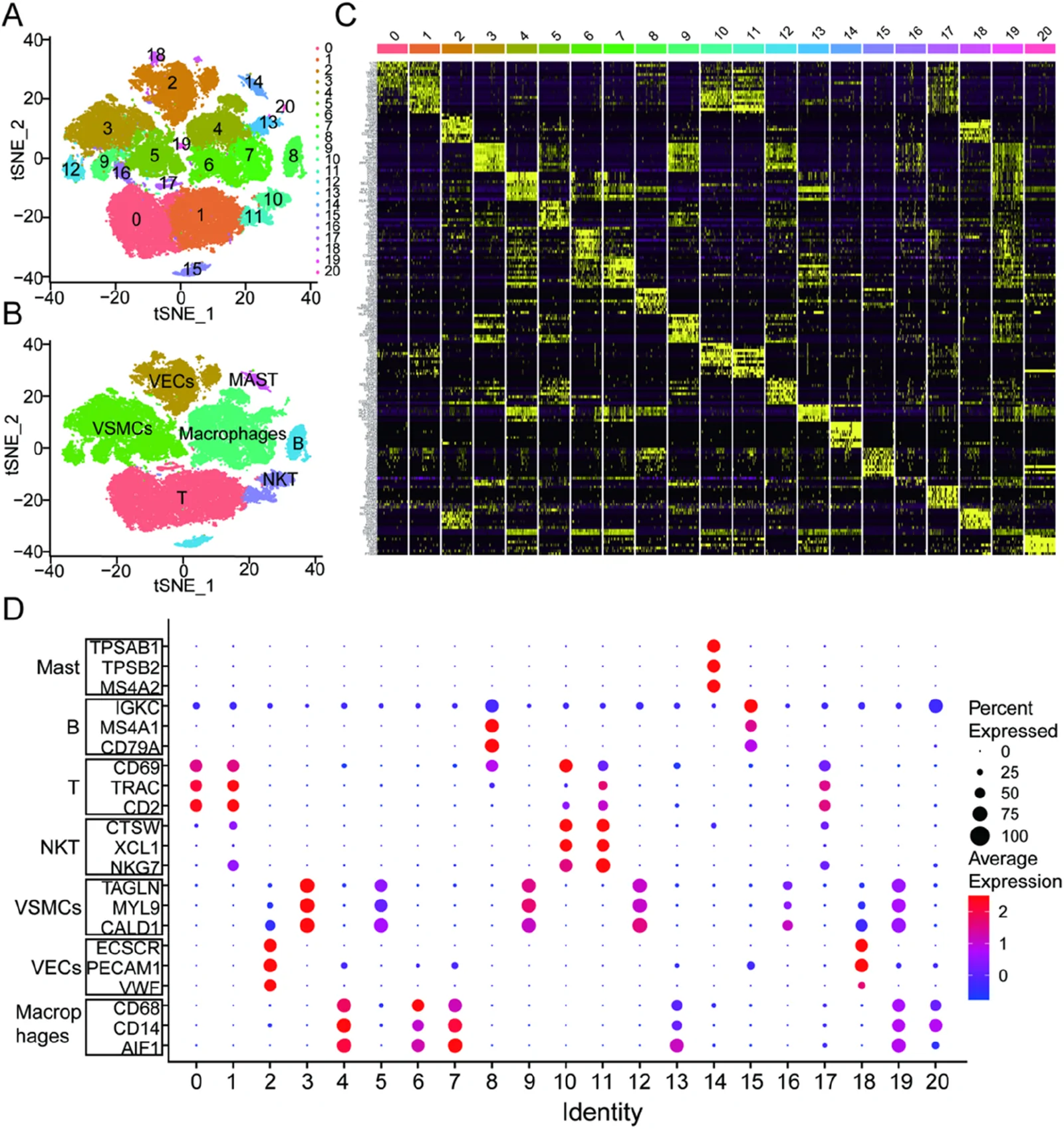
Cell subpopulations in human carotid artery AS plaques and adjacent regions via scRNA-seq. **(A)** t-SNE plot showing 21 distinct cell clusters. **(B)** t-SNE plot depicting cell types enriched with specific marker genes. **(C)** Heatmap displaying the top 10 genes for each of the 21 cell clusters. **(D)** Bubble plot showing the expression of characteristic genes within each cell cluster.

The scRNA-seq datasets GSE155512 and GSE159677 were retrieved from the Gene Expression Omnibus (GEO) database. The GSE155512 dataset consists of three human carotid artery AS plaque samples, whereas the GSE159677 dataset includes three human carotid artery AS plaque samples and three human carotid artery AS plaque adjacent samples. All samples were integrated into a t-SNE module (Fig. S1C), and t-SNE dimensionality reduction with unsupervised clustering was applied to all 54,701 cells, resulting in the identification of 21 distinct clusters (Fig. 1A). On the basis of known marker genes, these clusters were categorized into seven cell types: macrophages: CD68, CD14, and ALF1; VECs: ECSCR, PECAM1, and VWF; VSMCs: TAGLN, MYL9, and CALD1; NKT: CTSW, XCL1, and NKG7; T: CD69, TRAC, and CD2; B: IGKC, MS4A1, and CD79A; and Mast: TPSAB1, TPSB2, and MS4A2 (Fig. 1B). The “Find All Markers” function identified the top 10 highly expressed genes in each cluster, revealing specific gene expression patterns unique to each cluster (Fig. 1C). Bubble plots further displayed the expression of three marker genes in each cluster and further validated the accuracy of the cell cluster annotations (Fig. 1D).

### Interactions among different cell types in AS plaques and adjacent regions

**Figure 2:**
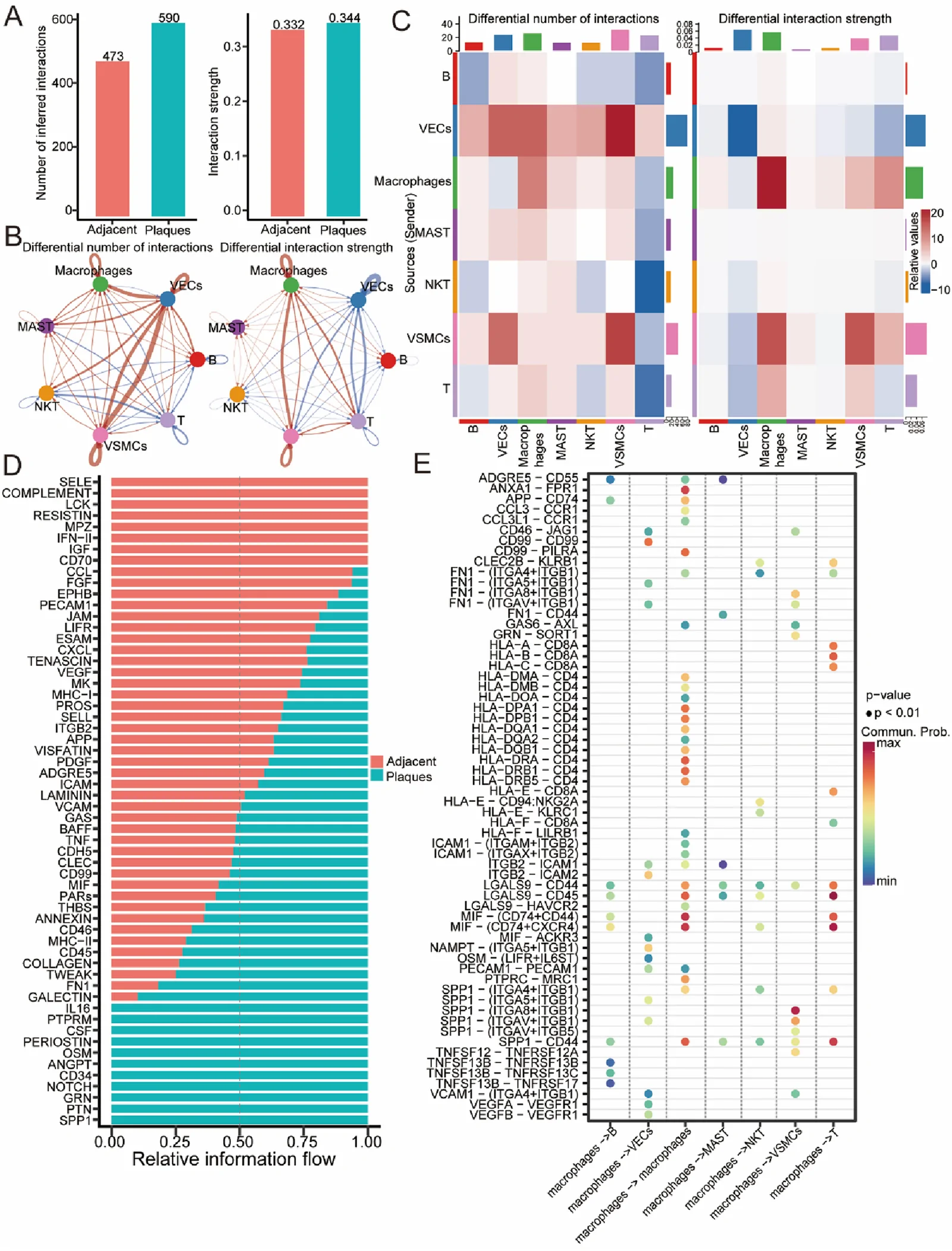
Cellular communication in adjacent and AS plaque regions. **(A)** Total number and intensity of interactions among different cell types in AS plaques and adjacent regions. **(B)** Network diagram illustrating changes in cell interactions between the AS plaque and adjacent regions. Red indicates an increase in the number or intensity of interactions, whereas blue indicates a decrease. **(C)** Heatmap of alterations in cell interactions between AS plaques and adjacent regions. **(D)** Enrichment analysis of ligand–receptor interactions at the signaling pathway level among various cell types in AS plaques and adjacent regions. **(E)** Overview of ligand–receptor interactions between macrophages and other cells in atherosclerotic plaque regions. *∗∗p* < 0.01.

The number and intensity of intercellular interactions were significantly elevated in the AS plaques (Fig. 2A). VECs presented a significant increase in the number of interactions with other cell types, whereas macrophages presented a marked increase in the intensity of these interactions (Fig. 2B). VECs, macrophages, VSMCs, and T cells presented the most substantial changes in both the number and intensity of intercellular interactions, particularly in AS plaques (Fig. 2C). Additionally, 11 signaling pathways were conserved and specifically enriched in the AS plaques, whereas 8 pathways were enriched in adjacent regions (Fig. 2D). Macrophages in AS plaques exhibited notably increased interactions with other cells through high relatively high levels of secreted phosphoprotein 1 (SPP1), with the corresponding receptor integrin subunit alpha 8 (ITGα8) ^+^/integrin β1 (ITGβ1) widely expressed in VSMCs. These findings suggest that macrophages may communicate with VSMCs via the SPP1/ITGα8^+^/ITGβ1 axes during the progression of AS plaques. SPP1, also known as macrophage-derived osteopontin (OPN), plays a critical regulatory role in AS calcification and plaque formation.^32^ Elevated expression of SPP1 by macrophages in AS plaques promotes the proliferation and migration of VSMCs, contributing to vascular hyperplasia and plaque progression.^33^ ITGα8 and ITGβ1, which are highly expressed in VSMCs, mediate transmembrane connections between the extracellular matrix and cytoskeleton and play vital roles in the initiation and progression of AS.^34^ They also regulate the transdifferentiation of VSMCs into other cell types, thereby influencing the development and progression of AS.^35^ These data suggest that the SPP1/ITGα8^+^/ITGβ1 axis plays a crucial role in AS plaque formation (Fig. 2E).

### Cellular heterogeneity among different cell types in AS plaques and adjacent regions

VECs, VSMCs, T cells, and macrophages play crucial roles in the progression of AS plaque formation, which is consistent with previous reports.^10^ scRNA-seq analysis further revealed significant transcriptional heterogeneity among these cell types in AS plaques and adjacent regions. Specifically, compared with those in cells from adjacent regions, 172, 209, 66, and 210 genes were overexpressed in VECs, VSMCs, T cells, and macrophages, respectively, in atherosclerotic plaque regions (Tables S2–S5).

**Figure 3:**
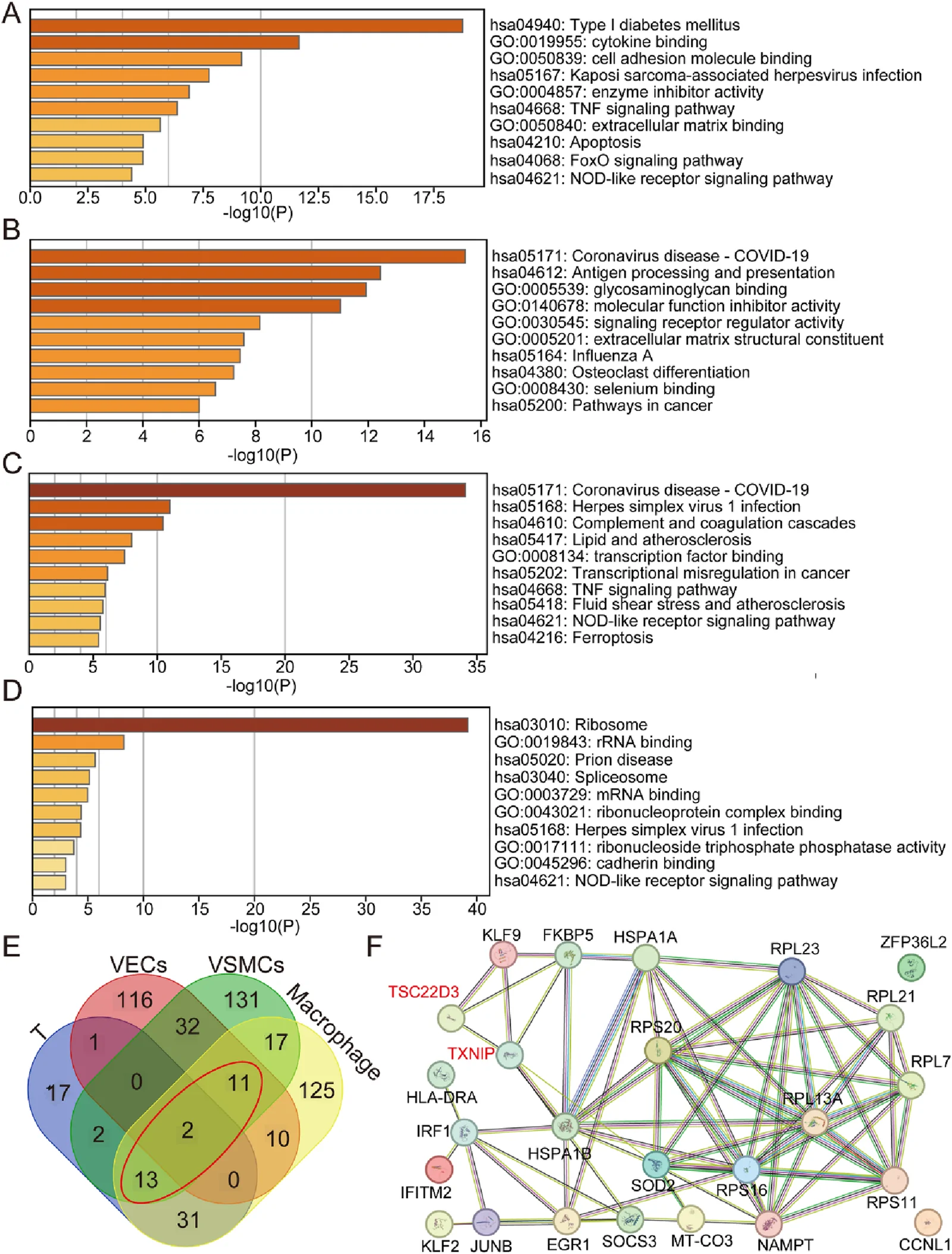
DEGs in VSMCs, VECs, T cells, and macrophages in atherosclerotic plaque regions compared with adjacent regions. **(A)** GO molecular function and KEGG signaling pathway enrichment of upregulated genes in VECs in the atherosclerotic plaque region. **(B)** GO molecular function and KEGG signaling pathway enrichment of upregulated genes in VSMCs in the atherosclerotic plaque region. **(C)** GO molecular function and KEGG signaling pathway enrichment of upregulated genes in macrophages in the atherosclerotic plaque region. **(D)** GO molecular function and KEGG signaling pathway enrichment of upregulated genes in T cells in the atherosclerotic plaque region. **(E)** Venn diagram of upregulated genes in VECs, VSMCs, macrophages, and T cells within the atherosclerotic plaque region. **(F)** PPI analysis of coupregulated genes in VECs, VSMCs, macrophages, and T cells within the atherosclerotic plaque region.

Further analysis of KEGG signaling pathways and GO molecular functions revealed cell type-specific pathway enrichment in AS plaques compared with adjacent regions. VECs were significantly enriched in immune-related pathways, such as those related to type I diabetes mellitus, TNF signaling, NOD-like receptor signaling, and extracellular matrix interactions (Fig. 3A). VSMCs were enriched in viral response pathways, such as COVID-19, influenza A, and extracellular matrix organization (Fig. 3B). Macrophages exhibit diverse levels of activation of lipid metabolism, ferroptosis, and shear stress responses (Fig. 3C). T cells were uniquely enriched in the ribosomal and spliceosome pathways (Fig. 3D). These distinct molecular signatures highlight the specialized roles of each cell type in plaque pathophysiology.

A Venn diagram of the genes upregulated in at least three of the four cell types revealed 25 key genes potentially involved in the development and progression of AS (Fig. 3E). PPI analysis of these genes revealed multiple ribosomal proteins involved in protein synthesis, as well as proteins related to oxidative stress and the immune response (Fig. 3F).

### CMAP identified narciclasine as a candidate drug for AS therapy

**Figure 4:**
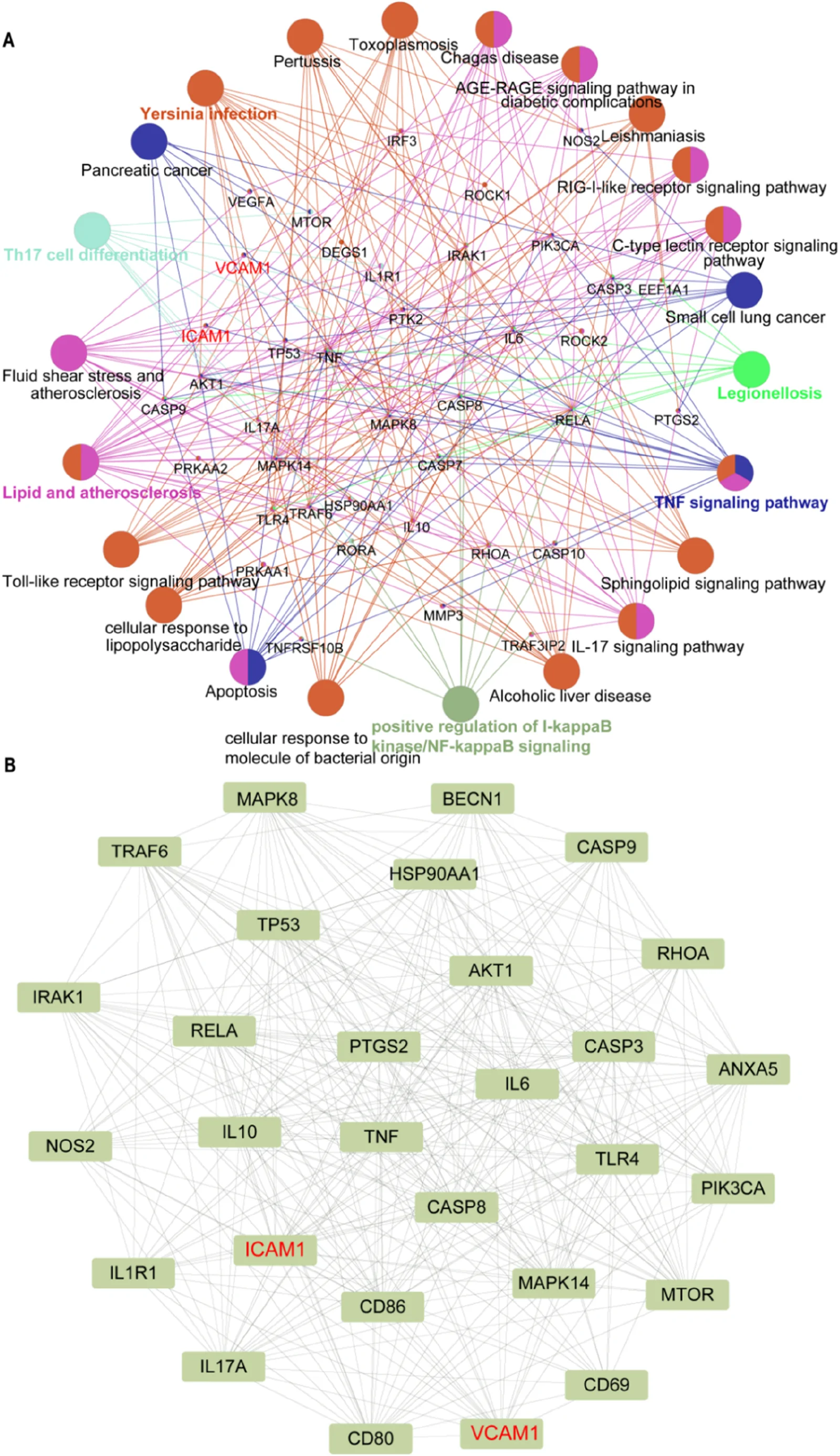
Functional analysis of narciclasine targets. **(A)** Regulatory network of narciclasine targets. The colored circles represent signaling pathways or biological processes, and the associated signaling pathways or biological processes share the same color. The lines connected to circles of the same color represent the differentially expressed genes of that signaling pathway. **(B)** MCODE clustering analysis of narciclasine drug targets on the basis of network topology.

On the basis of the DEGs identified across multiple cell types in atherosclerotic plaque regions, potential therapeutic drugs for AS were screened via CMAPs (Table S6). Narciclasine emerged as the most promising candidate, with a score of 99.75. The targets of narciclasine were subsequently identified by integrating data from databases such as BindingDB, PubChem, STITCH, TargetNet, and SwissTarget, resulting in 108 unique targets (Table S7). A drug-target regulatory network was constructed, highlighting that narciclasine’s therapeutic targets were predominantly associated with signaling pathways and biological processes such as the TNF signaling pathway, lipid and atherosclerosis, Yersinia infection, Legionellosis, Th17 cell differentiation, and positive regulation of I-kappa B kinase/NF-kappa B signaling (Fig. 4A). These target genes are involved mainly in inflammation, antibacterial responses, lipid metabolism, and AS. Narciclasine target clustering via MCODE analysis revealed that the most significant modules were associated with anti-inflammatory effects (Fig. 4B). Among these molecules, VCAM-1 and ICAM-1 are located at the core of drug targets.

### Network pharmacology and machine learning analysis revealed that narciclasine potentially targets VCAM-1/ICAM-1 to treat AS

**Figure 5:**
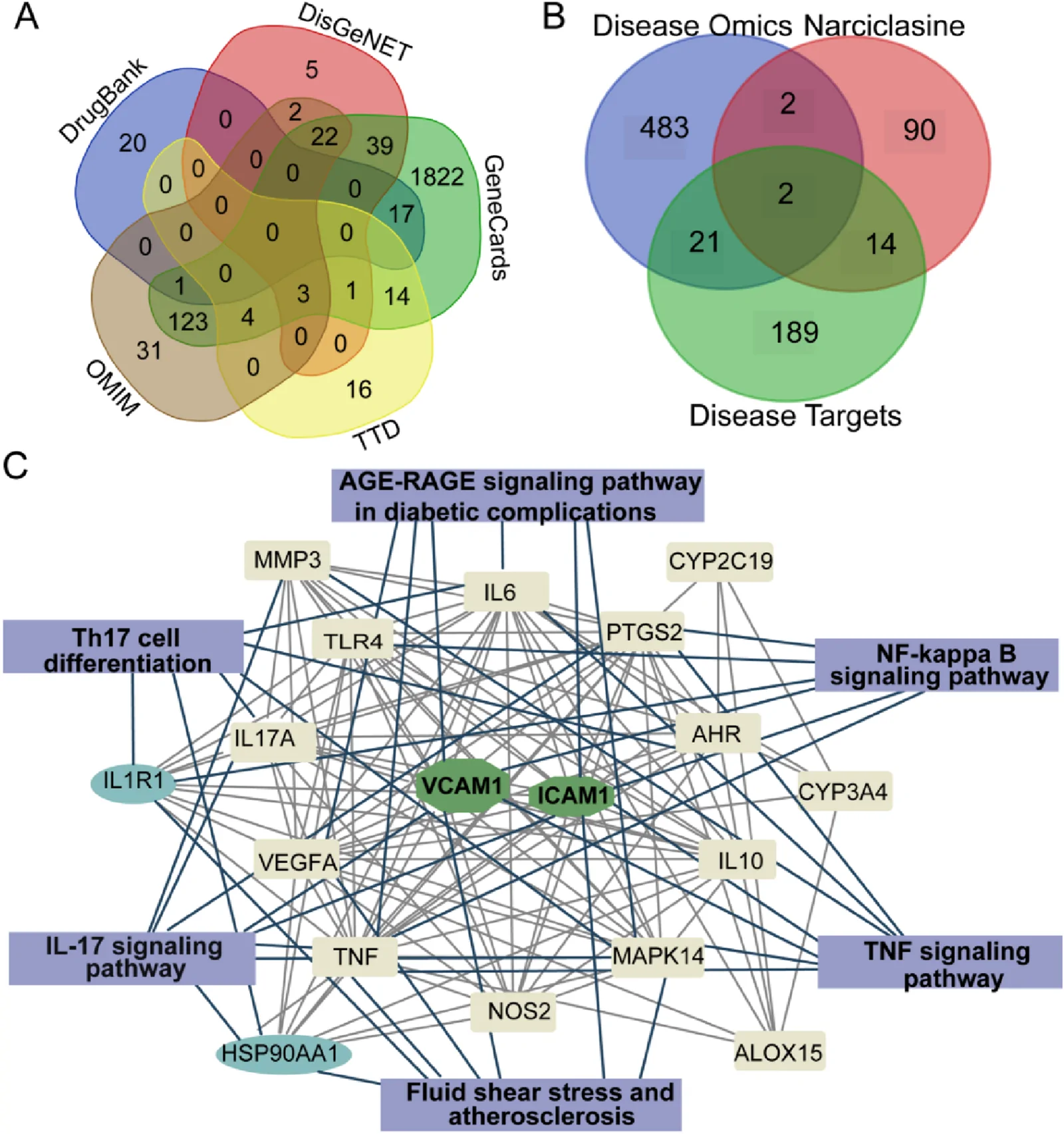
Network pharmacology analysis of the key anti-AS targets of narciclasine. **(A)** Venn diagram of AS-related targets collected from the DrugBank, DisGeNET, GeneCards, OMIM, and TTD databases. **(B)** Overlapping genes among narciclasine targets, AS targets, and genes upregulated in AS plaques. **(C)** Network pharmacology map showing interactions among genes and signaling pathways. The purple rectangles represent signaling pathways; the green octagons represent common genes across all three categories; the blue ellipses indicate overlapping genes between narciclasine targets and highly expressed genes in AS plaque regions; and the light khaki quadrilaterals represent overlapping genes between narciclasine targets and AS targets.

Network pharmacology analysis identified potential therapeutic targets of narciclasine for AS. Targets found in at least two AS-related databases were selected as candidate genes (Fig. 5A). Notably, ICAM-1 and VCAM-1 were shared across narciclasine targets, AS-related targets and genes highly expressed in AS plaques (Fig. 5B). KEGG and PPI analyses further revealed that VCAM-1 and ICAM-1 were central hub genes, suggesting their key roles in narciclasine’s anti-AS effects (Fig. 5C). Furthermore, we also applied machine learning algorithms to identify core target genes of narciclasine for AS treatment. To assess the validity of the machine learning models, we evaluated the residuals and ROC curves. Smaller residuals indicate better model performance. In terms of residual size, the machine learning models were ranked as follows: XGB ≈ SVM > RF > GLM (Fig. S2A–B). A larger ROC value suggested better performance, with values greater than 0.7 considered acceptable. The models were ranked as follows: RF = SVM > XGB > GLM (Fig. S2C). The SVM model was ultimately selected, and the key genes identified by SVM were ICAM1, SOD2, TLR4, IL1R1, CCL4, IRF1, CCL3, IL1B, VCAM1, and TNF (Fig. S2D). The machine learning results further confirmed that network pharmacology predicted the potential of narciclasine, which targets VCAM-1/ICAM-1 to treat AS.

### Analysis of the binding affinity between narciclasine and VCAM-1/ICAM-1

**Figure 6:**
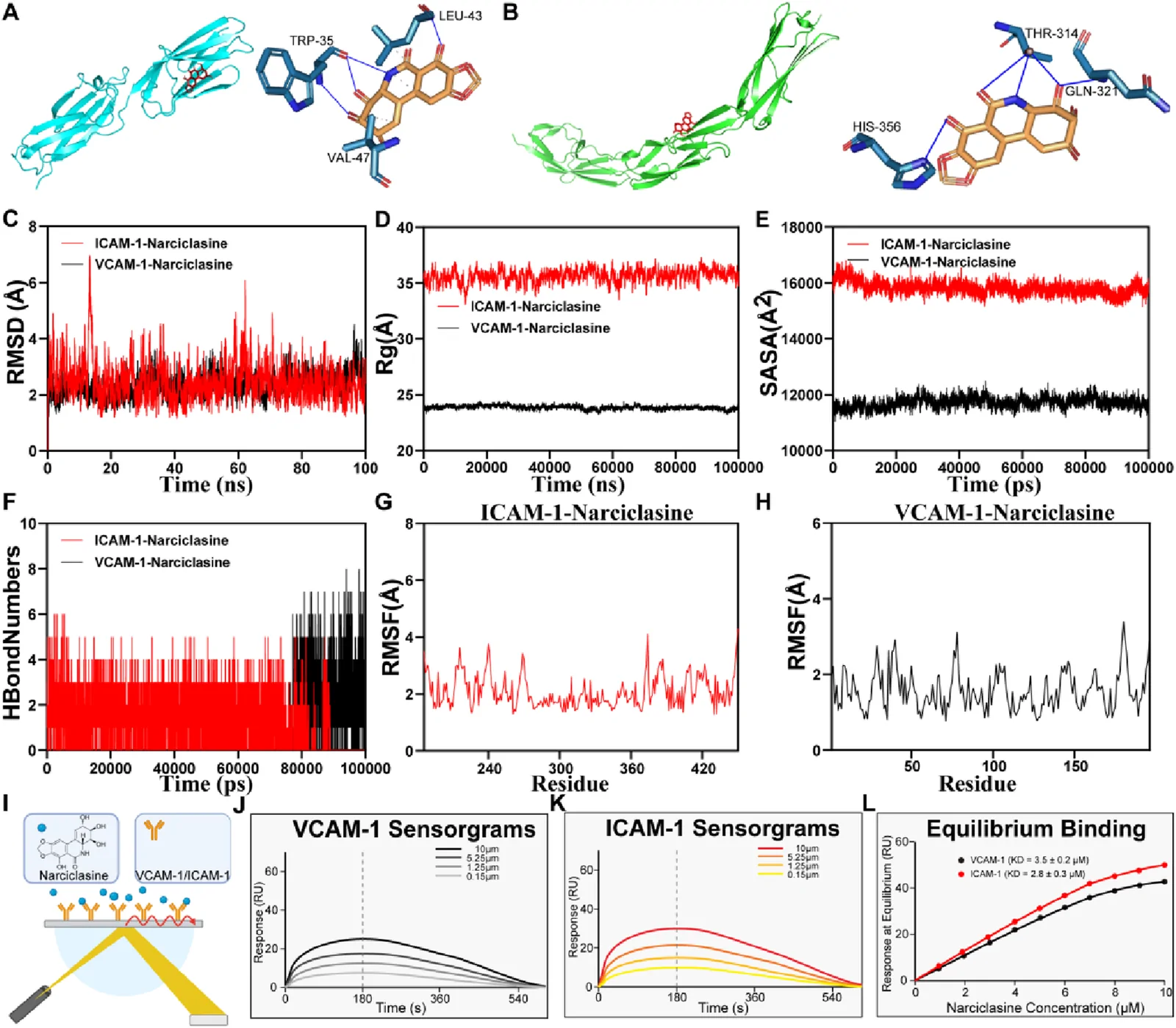
Molecular docking, MDS, and SPR confirmed VCAM-1 and ICAM-1 as targets of narciclasine. **(A)** Molecular docking models of narciclasine binding to the VCAM-1 protein. **(B)** Molecular docking models of narciclasine binding to the ICAM-1 protein. **(C)** RMSD analysis assessing the conformational stability of the ICAM-1–narciclasine and VCAM-1-narciclasine complexes. **(D)** Rg analysis evaluating overall structural changes and protein compactness. **(E)** SASA analysis of the interactions between target proteins and narciclasine. **(F)** Hydrogen bond network analysis of both the ICAM-1-narciclasine and VCAM-1-narciclasine complexes. **(G**, **H)** RMSF analysis of amino acid residue flexibility in the ICAM-1-narciclasine and VCAM-1-narciclasine complexes. **(I)** Schematic diagram illustrating the SPR setup. **(J**, **K)** Sensograms of VCAM-1 and ICAM-1 expression in response to narciclasine (0.15–10 μM) at 25 °C. **(L)** Equilibrium binding analysis of ICAM-1-narciclasine and VCAM-1-narciclasine interactions.

A comprehensive analysis combining network pharmacology and machine learning identified VCAM-1 and ICAM-1 as the core targets of narciclasine in the treatment of AS. Molecular docking revealed strong interactions between narciclasine and VCAM-1 (score: 7.1) and ICAM-1 (score: 7.8), both of which exceeded the threshold score of 7 for high-affinity binding (Fig. 6A and B). Mathematical modeling further demonstrated that dual inhibition of VCAM-1 and ICAM-1 resulted in greater suppression of leukocyte migration than inhibition of either molecule alone (Fig. S3). These findings support the role of ICAM-1 and VCAM-1 as key therapeutic targets of narciclasine in AS. MDS revealed that the ICAM-1-narciclasine complex stabilized rapidly (∼20 ns) with consistent RMSD fluctuations of approximately 2.3 Å, whereas the VCAM-1-narciclasine complex stabilized more slowly (∼75 ns) near 2.8 Å (Fig. 6C), indicating favorable binding. Rg analysis confirmed stable tertiary structures (Fig. 6D), and SASA analysis revealed initial fluctuations followed by stabilization, which was consistent with conformational adjustment during binding (Fig. 6E). Hydrogen bond analysis revealed 0–6 bonds for ICAM-1 and 0–8 bonds for VCAM-1, with both averaging ∼4 bonds, indicating complex stability (Fig. 6F). The RMSF values remained below 3 Å for most residues, indicating limited mobility and increased rigidity upon binding (Fig. 6G and H). Overall, the simulations confirmed the formation of stable, energetically favorable complexes between narciclasine and both adhesion molecules.

SPR analysis validated these interactions (Fig. 6I), with sensorgrams fitting well to a 1:1 Langmuir binding model (*χ*^2^ = 1.74 for VCAM-1; *χ*^2^ = 2.05 for ICAM-1). The association rates were 2.3 × 10^3^ M^−1^s^−1^ and 3.1 × 10^3^ M^−1^s^−1^, and the dissociation rates were 8.1 × 10^−3^ s^−1^ and 8.7 × 10^−3^ s^−1^ for VCAM-1 and ICAM-1, respectively (Fig. 6J and K). Steady-state analysis confirmed the 1:1 binding stoichiometry (Fig. 6L). Thermodynamic profiling revealed enthalpy-driven binding (ΔH° = −22.8 ± 1.5 kJ/mol for VCAM-1 and -25.6 ± 1.3 kJ/mol for ICAM-1) and micromolar binding affinities (KD = 3.5 ± 0.21 μM and 2.8 ± 0.3 μM) (Table S8), which is consistent with the hydrogen bonding patterns predicted via simulation. These findings experimentally confirm the strong and specific interactions between narciclasine and VCAM-1/ICAM-1.

### Narcissine protected the endothelium and promoted AS plaque regression by inhibiting VCAM-1/ICAM-1

**Figure 7:**
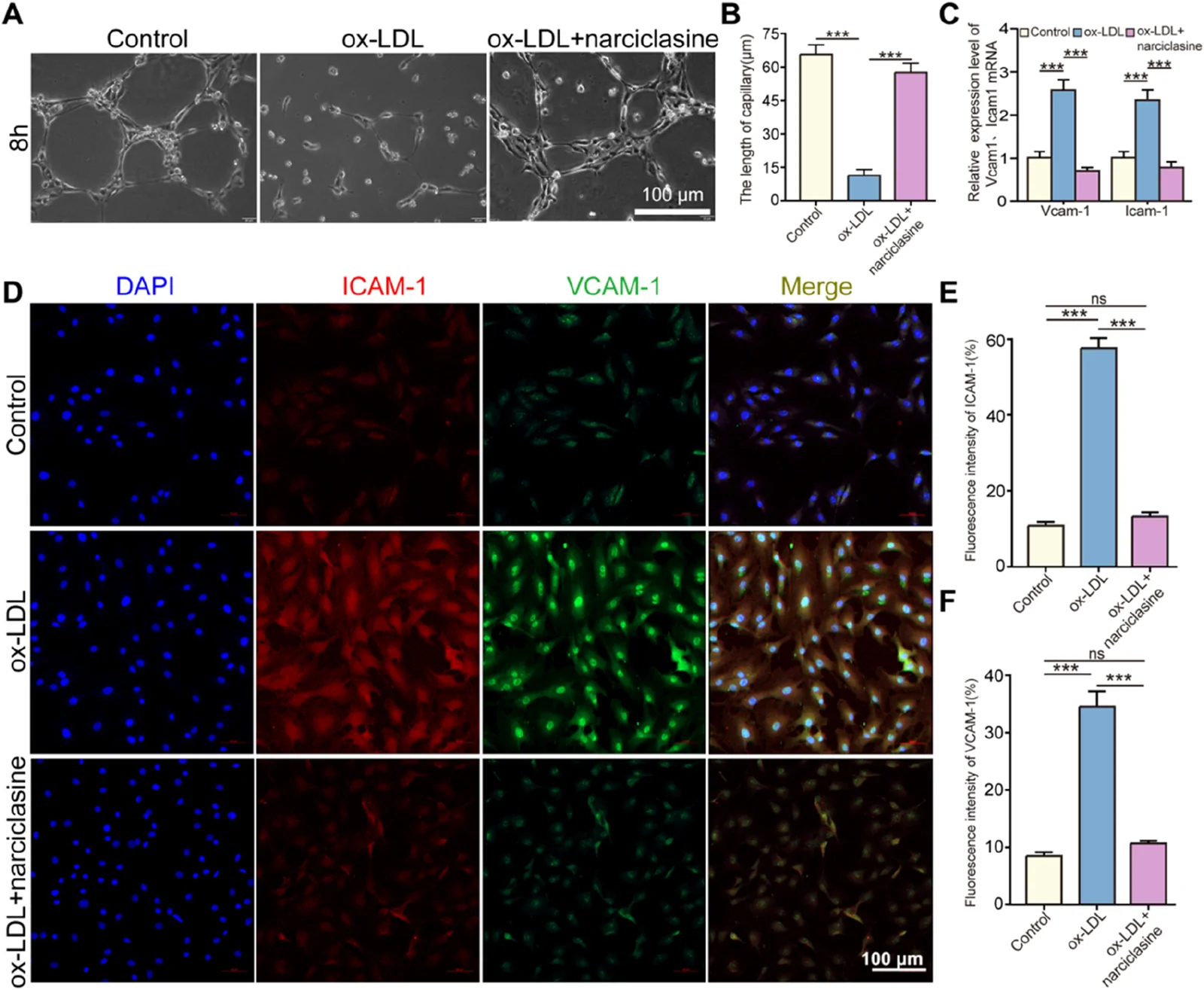
Narciclasine alleviated vascular injury by inhibiting VCAM-1/ICAM-1 *in vitro*. **(A)** Phase contrast images showing HUVEC tube formation in the control, ox-LDL-treated, and narciclasine-treated ox-LDL groups. **(B)** Semiquantification analysis of capillary length in the three groups (ANOVA test, *n* = 5, *∗∗∗p* = 5.42 × 10^−8^. Mean ± SD, control: 63.96 ± 5.06, ox-LDL: 12.52 ± 1.85; ox-LDL + narciclasine: 57.90 ± 4.84). **(C)** Relative gene expression levels of ICAM-1(ANOVA test, *n* = 4, ∗∗∗*p* = 3.5 × 10^−10^. Mean ± SD, control: 1 ± 0.04, ox-LDL: 2.58 ± 0.15; ox-LDL + narciclasine: 0.63 ± 0.02) and VCAM-1 (ANOVA test, *n* = 4, ∗∗∗*p* = 2.55 × 10^−9^. Mean ± SD, control: 1 ± 0.04, ox-LDL: 2.28 ± 0.14; ox-LDL + narciclasine: 0.73 ± 0.04) in HUVECs in the three groups. **(D)** Immunofluorescence costaining images of ICAM-1 and VCAM-1. **(E**, **F)** Semiquantification analysis of the immunofluorescence intensity of ICAM-1 (ANOVA test, *n* = 6, *∗∗∗p* = 0.0036. Mean ± SD, control: 11.14 ± 0.55, ox-LDL: 55.89 ± 2.01; ox-LDL + narciclasine: 13.59 ± 0.74) and VCAM-1 (ANOVA test, *n* = 6, ∗∗∗*p* = 0.0024. Mean ± SD, control: 4.61 ± 0.27, ox-LDL: 34.56 ± 2.63; ox-LDL + narciclasine: 5.78 ± 0.15).

Given that VCAM-1 and ICAM-1 are expressed predominantly by VECs and play important roles in AS development,^36^ we investigated the effects of narciclasine on these adhesion molecules in VECs. An AS cell model was established by inducing VECs with oxidized low-density lipoprotein (ox-LDL). Vascular tube formation assays revealed that narciclasine treatment significantly alleviated ox-LDL-induced endothelial damage, with capillary lengths comparable to those in the control group (Fig. 7A and B). qPCR and immunofluorescence analysis demonstrated that ox-LDL markedly increased VCAM-1 and ICAM-1 expression in HUVECs, whereas narciclasine treatment significantly reduced their expression levels, restoring them to near-control levels (Fig. 7C–F).

**Figure 8:**
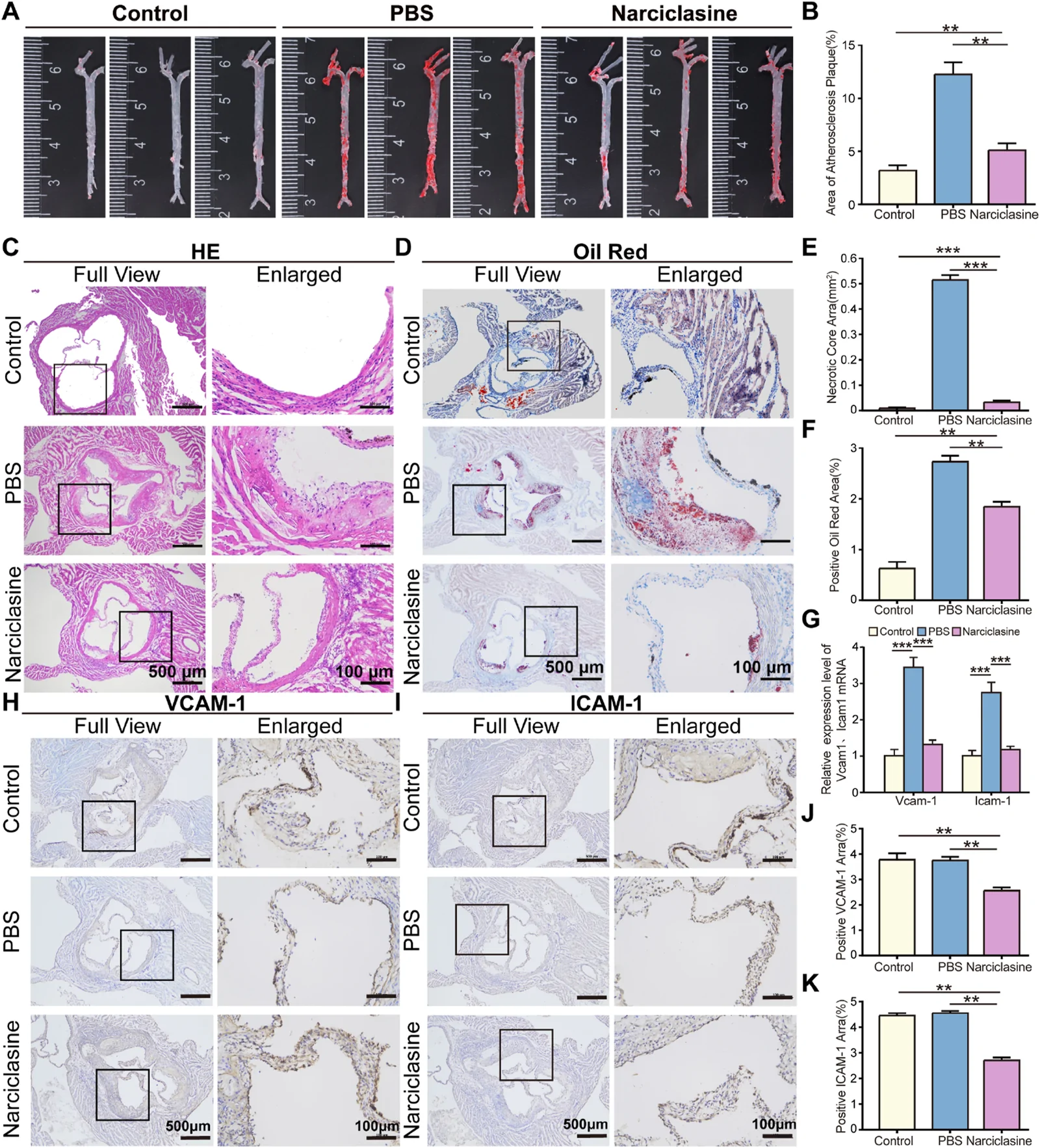
Narciclasine alleviated AS progression by targeting VCAM-1/ICAM-1 *in vivo*. **(A)** Oil Red O stained images of whole aortas from control, PBS-treated, and narciclasine-treated *ApoE*^*−/−*^ mice. **(B)** Semi-quantification analysis of the atherosclerotic plaque areas (ANOVA test, *n* = 4, ∗∗*p* = 0.0004. Mean ± SD, control: 3.2 ± 0.54, PBS: 12.27 ± 1.02; narciclasine: 5.16 ± 0.66). **(C)** HE images of aortic root sections from control, PBS-treated, and narciclasine-treated mice. **(D)** Oil red O images of aortic root sections from control, PBS-treated, and narciclasine-treated mice. **(E)** Semiquantification of the necrotic core areas (ANOVA test, *n* = 4, ∗∗∗*p* = 0.0002. Mean ± SD, control: 0.0049 ± 0.0004, PBS: 0.52 ± 0.015; narciclasine: 0.033 ± 0.0036). **(F)** Semiquantification of the oil red O area of lipid-rich areas in aortic root sections (ANOVA test, *n* = 4, ∗∗*p* = 0.0015. Mean ± SD, control: 0.64 ± 0.114, PBS: 2.74 ± 0.108; narciclasine: 1.84 ± 0.103). **(G)** Relative gene expression levels of VCAM-1 (ANOVA test, *n* = 4, ∗∗∗*p* = 0.001. Mean ± SD, control: 1 ± 0.05, PBS: 3.42 ± 0.17; narciclasine: 1.25 ± 0.03) and ICAM-1 across the three groups (ANOVA test, *n* = 4, ∗∗∗*p* = 0.001. Mean ± SD, control: 1 ± 0.04, PBS: 2.84 ± 0.16; narciclasine: 1.18 ± 0.03). **(H, I)** Immunohistochemistry images of VCAM-1 and ICAM-1 in the aortic roots. **(J, K)** Semiquantification of VCAM-1 (ANOVA test, *n* = 5, ∗∗*p* = 0.001. Mean ± SD, control: 4.47 ± 0.075, PBS: 4.56 ± 0.074; narciclasine: 2.72 ± 0.087) and ICAM-1 (ANOVA test, *n* = 4, ∗∗*p* = 0.0066. Mean ± SD, control: 3.79 ± 0.173, PBS: 3.73 ± 0.11; narciclasine: 2.54 ± 0.108) protein expression in the aortic root.

Furthermore, we investigated the effect of narciclasine on high-fat diet-fed *ApoE*^*−/−*^ mice. The whole aorta Oil Red O staining results (Fig. 8A, B) revealed alleviation of plaque accumulation, compared to HFD-fed *ApoE*^*−/−*^ mice that received PBS. Additionally, the control mice that received a normal diet were significantly different from those in the above groups. These results suggested that narciclasine was able to inhibit AS progression. Moreover, HE and Oil Red O staining of aortic root sections revealed that narciclasine administration significantly reduced the necrotic core size and lipid accumulation compared with those in the PBS-treated group (Fig. 8C–F), indicating the suppression of AS plaque formation. Furthermore, the gene and protein expression levels of VCAM-1 and ICAM-1 were markedly lower in narciclasine-treated mice than in PBS-treated controls (Fig. 8G–K). These findings, both *in vitro* and *in vivo*, suggest that narciclasine mitigates AS progression by downregulating VCAM-1 and ICAM-1.

## Discussion

AS is the leading cause of morbidity and mortality worldwide.^6^^,^^37^ Current treatment strategies primarily include lipid-lowering drugs (such as statins), antiplatelet drugs and blood pressure control.^38^ However, these treatments predominantly aim to manage disease progression rather than reverse damage. Moreover, damaged VEC regeneration is a slow and insufficient process.^39^ Notably, a substantial proportion of patients continue to experience adverse cardiovascular events despite achieving target lipid levels, a phenomenon termed “residual risk”.^40^ Therefore, there is a critical need for novel, effective, and well-tolerated therapies to prevent or improve long-term outcomes and survival in patients with AS.

In this study, we introduce a novel tripartite drug discovery paradigm that integrates single-cell transcriptomics, computational pharmacology, and multidimensional experimental validation, identifying narciclasine as a promising therapeutic candidate for AS. Narciclasine (also known as lycoricidinol) is a natural alkaloid found in daffodils and other Amaryllidaceae family plants that has potent anticancer effects on the brain, skin, and breast tumors and colon carcinoma.^41^^,^^42^ Previous research has identified eukaryotic translation elongation factor 1 alpha 1 (eEF1A1) as a direct molecular target of narciclasine,^43^ which was also predicted as a potential target in our research (Table S7; Fig. 4A). Importantly, eEF1A1 modulates macrophage glycolytic reprogramming, a key process in AS pathophysiology.^44^ Network pharmacology and bioinformatics analyses further revealed that narciclasine influences multiple key signaling pathways implicated in AS, including the TNF signaling pathway, lipid metabolism, and AS. These signaling pathways are commonly involved in the progression of AS, indicating that narciclasine is highly promising for the treatment of AS.

Further integration of narciclasine targets, AS-associated genes, and plaque-upregulated genes identified VCAM-1 and ICAM-1 as core molecular targets. VCAM-1 and ICAM-1 are immunoglobulin superfamily adhesion molecules critical for leukocyte adhesion to VECs.^45^ These proteins facilitate leukocyte recruitment and transmigration during inflammatory responses.^46^ In early AS, their upregulation on activated VECs promotes monocyte adhesion and infiltration, initiating plaque development.^3^, ^4^, ^5^, ^6^^,^^36^ Inhibition of these molecules has been shown to reduce the plaque burden in preclinical models. For example, magnolol decreased VCAM-1 expression by ∼50%, whereas probucol reduced VCAM-1 mRNA levels by up to 64.8% ± 1.9%, both of which resulted in substantial plaque attenuation.^47^ TNF-α inhibitors also disrupt VCAM-1/ICAM-1 expression by blocking TNF-α receptor signaling.^48^ These factors that inhibit VCAM-1/ICAM-1 to remedy AS prompted us to investigate the therapeutic potential of narciclasine. Molecular docking, MDS, and SPR experiments confirmed the strong binding affinities of narciclasine for VCAM-1 and ICAM-1.

Both *in vitro* and *in vivo* experiments demonstrated that narciclasine mitigated ox-LDL-induced endothelial damage and reduced plaque formation in AS model mice by downregulating VCAM-1 and ICAM-1 expression. These effects were comparable to those observed with magnolol and TNF-α inhibitors.^47^^,^^48^ Notably, our findings align with recent research indicating that narciclasine inhibits AS progression in elderly individuals by delaying VSMC senescence, as identified through large-scale GEO data analyses and CMAP predictions.^49^ This parallel evidence not only reinforces the validity of our findings but also suggests a potential anti-aging mechanism for narciclasine in AS therapy. However, clinical translation remains a challenge. Narciclasine has not yet been evaluated in human clinical trials, and its limited natural supply—primarily its isolation from daffodil bulbs—poses poses a bottleneck. Encouragingly, recent advancements in synthetic chemistry have enabled the organic synthesis of narciclasine and its analogues.^50^ Thus, the path toward the clinical application of narciclasine in tumor or AS treatment appears increasingly feasible. Although narciclasine is generally well-tolerated at the prescribed dose, it has a narrow therapeutic window, and adverse reactions are an important reason limiting its clinical application. Adverse reactions to narciclasine are preventable and controllable. By integrating therapeutic drug monitoring technologies and artificial intelligence algorithms, personalized narciclasine medication models based on genetic background and metabolic characteristics can be constructed to systematically analyze the molecular mechanisms of adverse reactions and establish a dose–response/toxicity prediction system. Based on multi-dimensional mechanism analysis and technological innovation, future research can explore the synergistic effects of narciclasine with antiviral drugs and cardiovascular protectants, and develop novel compound formulations or targeted delivery systems.

## Conclusions

In conclusion, our study revealed narciclasine as a promising therapeutic drug for AS. Through a multilayered approach involving scRNA-seq data from clinical AS samples, CMAP screening, network pharmacology, molecular docking, machine learning, dynamics simulations, and SPR analysis, we demonstrated that VCAM-1 and ICAM-1 are direct targets of narciclasine. These findings were further validated through experimental assays in cell and mouse models. This integrative strategy represents a novel framework for disease-specific drug discovery, offering valuable insights for the development of new anti-AS therapies.

## CRediT authorship contribution statement

**Yang Wang:** Writing – original draft, Methodology, Conceptualization. **Zhen Cao:** Writing – original draft, Methodology, Conceptualization. **Huai Lan:** Data curation. **Bo Li:** Data curation. **Guixue Wang:** Supervision, Funding acquisition. **Qingmei Chen:** Writing – review & editing, Writing – original draft.

## Data availability

The datasets presented in this study can be found in online repositories. The names of the repositories/repositories and accession number(s) can be found in the article/Supplementary material.

## Ethics declaration

The animal study was approved by the Ethics Committee of Chongqing University (Approval Code: A-2019-021) and adhered to NIH guidelines for the care and use of laboratory animals.

## Acknowledgements

The Graphical Abstract was created in BioRender. Wang, Y. (2025) https://BioRender.com/xeprvps, the agreement number: QO28P51GZW.

## Funding

This work was financially supported by the 10.13039/501100001809National Natural Science Foundation of China (No. 32471366), 10.13039/501100002338Fundamental Research Funds for the Central Universities (No. 2023CDJYGRH-YB13), Starting Grants for Research (No. 2023qdjfxm004) and the Science and Technology Innovation Project of Jinfeng Laboratory, Chongqing, China (No. jfkyjf202203001).

## Conflict of interests

Guixue Wang is a member of *Genes & Diseases* Editorial Board. To minimize bias, he/she was excluded from all editorial decision-making related to the acceptance of this article for publication. The remaining authors declare that they have no competing interests.
