# Supplementary material for: Single-cell sequencing-guided discovery of narciclasine as a dual VCAM-1/ICAM-1 inhibitor for atherosclerosis management
Source: Genes Dis. 2026 Mar 27;14(1):102167. doi: 10.1016/j.gendis.2026.102167 (PMC13589088; doi:10.1016/j.gendis.2026.102167)
Supplement: Supplementary file 1 [file mmc1.doc]

**Supplementary Materials**

**Supplementary Figures**

**Supplementary Figure 1.** Analysis process of the sc-RNA datasets. **(A)** Quality control of single-cell sequencing samples. **(B)** Elbow analysis of single-cell sequencing samples. **(C)** tSNE diagram of single-cell sequencing sample integration.

**Supplementary Figure 2.** **Identification of key targets via machine learning. (A)** Root mean square values of the residuals for the four machine learning models. The red dots represent the root mean square of the residuals. **(B)** Reverse cumulative distribution of the residuals for the four machine learning models. **(C)** ROC curves for the four machine learning models. **(D)** The ten most significant genes identified by the SVM.

**Supplementary Figure 3.** Mathematical model curves of the efficiency of narciclasine-mediated inhibition of VCAM-1, ICAM-1 and their combination.

**Supplementary Table 1.** **Primer sequences**

| **Gene** | **Forward primer** | **Reverse primer** |
| --- | --- | --- |
| **Human β-actin** | AAGCAGGAGTATGACGAGTCCG | GCCTTCATACATCTCAAGTTGG |
| **Human Vcam-1** | TCTCATTGACTTGCAGCACCACAG | CCTCATTCGTCACCTTCCCATTCAG |
| **Human Icam-1** | GGTAGCAGCCGCAGTCATAATGG | GTGGCTTGTGTGTTCGGTTTCATG |
| **Mouse β-actin** | AGTATCCATGAAAATAAGTGGTTACAGG | TTTGCAGTACATAATTTACACAGAAGC |
| **Mouse Vcam-1** | GGAGCCTGTCAGTTTTGAGAATG | TTGGGGAAAGAGTAGATGTCCAC |
| **Mouse Icam-1** | GATGGAGCAAGACTGTGAACAC | TGCTTGTGTCTACTGGATATCATG |

**Supplementary Table 2**. DEGs in VECs within atherosclerotic plaque tissue

| Gene | p_val | avg_log2FC | Percentage of cells expressing genes in Control | Percentage of cells expressing genes in AS | p_val_adj |
| --- | --- | --- | --- | --- | --- |
| ACKR1 | 7.51E-73 | -2.174428857 | 0.242 | 0.531 | 1.77E-68 |
| TXNIP | 3.23E-85 | -1.422481326 | 0.638 | 0.821 | 7.62E-81 |
| STC1 | 5.25E-38 | -1.318318733 | 0.136 | 0.345 | 1.24E-33 |
| HLA-DPA1 | 2.70E-61 | -1.317582473 | 0.648 | 0.787 | 6.37E-57 |
| HLA-DQA1 | 8.46E-50 | -1.281785544 | 0.115 | 0.353 | 2.00E-45 |
| HLA-DRA | 2.04E-61 | -1.191093577 | 0.689 | 0.819 | 4.82E-57 |
| RGS16 | 4.95E-33 | -1.167070432 | 0.227 | 0.427 | 1.17E-28 |
| CTSC | 1.12E-40 | -1.038591526 | 0.375 | 0.566 | 2.65E-36 |
| HLA-DPB1 | 8.87E-45 | -0.911397525 | 0.569 | 0.719 | 2.09E-40 |
| CD74 | 1.38E-49 | -0.876866321 | 0.884 | 0.934 | 3.27E-45 |
| HLA-DRB1 | 5.66E-53 | -0.859849632 | 0.878 | 0.923 | 1.33E-48 |
| SPARCL1 | 4.32E-60 | -0.845566697 | 0.884 | 0.917 | 1.02E-55 |
| PLAT | 3.61E-38 | -0.840872603 | 0.323 | 0.547 | 8.53E-34 |
| RAMP3 | 4.82E-51 | -0.83729008 | 0.539 | 0.756 | 1.14E-46 |
| SNCG | 3.03E-39 | -0.809689434 | 0.556 | 0.689 | 7.16E-35 |
| C2CD4B | 1.16E-08 | -0.796230244 | 0.229 | 0.31 | 0.000273194 |
| AQP1 | 3.65E-54 | -0.78524143 | 0.617 | 0.799 | 8.62E-50 |
| ADIRF | 1.14E-65 | -0.743694919 | 0.758 | 0.887 | 2.68E-61 |
| HLA-DQB1 | 4.27E-35 | -0.741130132 | 0.261 | 0.459 | 1.01E-30 |
| OLFM1 | 3.67E-31 | -0.708382344 | 0.252 | 0.441 | 8.67E-27 |
| IFITM1 | 1.36E-30 | -0.696751346 | 0.358 | 0.523 | 3.20E-26 |
| DAAM1 | 8.15E-26 | -0.686033624 | 0.271 | 0.43 | 1.92E-21 |
| ATF3 | 1.59E-23 | -0.679639457 | 0.285 | 0.446 | 3.76E-19 |
| CTSH | 6.42E-32 | -0.665521992 | 0.2 | 0.402 | 1.52E-27 |
| NFKBIA | 1.94E-17 | -0.659832119 | 0.788 | 0.842 | 4.59E-13 |
| RASA4 | 1.33E-36 | -0.650250555 | 0.079 | 0.275 | 3.13E-32 |
| HLA-DMA | 2.35E-31 | -0.640863857 | 0.372 | 0.547 | 5.54E-27 |
| ICAM1 | 6.62E-21 | -0.639007545 | 0.252 | 0.393 | 1.56E-16 |
| IRF1 | 2.68E-13 | -0.638999743 | 0.433 | 0.522 | 6.31E-09 |
| SPRY1 | 3.05E-28 | -0.635672262 | 0.371 | 0.568 | 7.20E-24 |
| PERP | 7.27E-23 | -0.601808426 | 0.192 | 0.346 | 1.72E-18 |
| IGFBP4 | 1.02E-34 | -0.590554522 | 0.87 | 0.923 | 2.40E-30 |
| HLA-DRB5 | 2.32E-37 | -0.571358735 | 0.683 | 0.811 | 5.47E-33 |
| TSPAN7 | 2.20E-26 | -0.571224426 | 0.532 | 0.661 | 5.19E-22 |
| NOSTRIN | 3.24E-29 | -0.570519249 | 0.199 | 0.392 | 7.65E-25 |
| GIMAP4 | 4.89E-28 | -0.550893655 | 0.558 | 0.687 | 1.15E-23 |
| LIFR | 2.16E-22 | -0.545723574 | 0.227 | 0.382 | 5.11E-18 |
| TESC | 1.82E-18 | -0.530817177 | 0.166 | 0.294 | 4.30E-14 |
| PDK4 | 8.03E-16 | -0.526564984 | 0.22 | 0.345 | 1.90E-11 |
| CCL14 | 3.03E-16 | -0.512758403 | 0.327 | 0.446 | 7.14E-12 |
| SH3BP5 | 1.00E-26 | -0.496241657 | 0.467 | 0.62 | 2.37E-22 |
| TMEM88 | 1.31E-20 | -0.495836253 | 0.134 | 0.278 | 3.09E-16 |
| HLA-B | 1.49E-40 | -0.484781202 | 0.983 | 0.988 | 3.51E-36 |
| PSMB9 | 1.10E-20 | -0.481094198 | 0.558 | 0.661 | 2.61E-16 |
| ITGA6 | 3.92E-19 | -0.478129578 | 0.294 | 0.443 | 9.26E-15 |
| CNN3 | 4.86E-21 | -0.477500757 | 0.551 | 0.652 | 1.15E-16 |
| EGR1 | 3.88E-10 | -0.476807292 | 0.543 | 0.61 | 9.16E-06 |
| ZFP36 | 1.50E-11 | -0.474836001 | 0.816 | 0.854 | 3.54E-07 |
| CST3 | 2.52E-24 | -0.471871374 | 0.926 | 0.956 | 5.95E-20 |
| SOCS3 | 4.08E-15 | -0.465587549 | 0.683 | 0.73 | 9.63E-11 |
| GSN | 3.66E-26 | -0.464979699 | 0.844 | 0.917 | 8.63E-22 |
| SYNE2 | 2.13E-23 | -0.463320335 | 0.422 | 0.589 | 5.03E-19 |
| CYP1B1 | 4.45E-05 | -0.457377671 | 0.268 | 0.323 | 1 |
| GIMAP7 | 5.59E-21 | -0.453269546 | 0.803 | 0.869 | 1.32E-16 |
| MGLL | 1.63E-11 | -0.451933585 | 0.405 | 0.498 | 3.85E-07 |
| CD320 | 1.77E-12 | -0.447195173 | 0.505 | 0.577 | 4.17E-08 |
| GADD45B | 8.75E-12 | -0.440721912 | 0.61 | 0.688 | 2.07E-07 |
| IL6ST | 5.36E-19 | -0.426465371 | 0.687 | 0.792 | 1.27E-14 |
| VCAM1 | 1.68E-06 | -0.424323304 | 0.263 | 0.331 | 0.039662629 |
| TSHZ2 | 4.46E-17 | -0.42367745 | 0.186 | 0.322 | 1.05E-12 |
| ETS2 | 1.95E-17 | -0.421639394 | 0.626 | 0.729 | 4.60E-13 |
| MMRN1 | 1.35E-10 | -0.421509194 | 0.329 | 0.426 | 3.18E-06 |
| JAM2 | 4.64E-23 | -0.421292176 | 0.535 | 0.653 | 1.09E-18 |
| TIMP3 | 1.27E-21 | -0.41971383 | 0.352 | 0.519 | 2.99E-17 |
| FBLN2 | 1.48E-16 | -0.417159233 | 0.246 | 0.391 | 3.49E-12 |
| SOD2 | 1.66E-09 | -0.416951043 | 0.313 | 0.404 | 3.93E-05 |
| HLA-A | 9.95E-35 | -0.412612571 | 0.97 | 0.983 | 2.35E-30 |
| BCAM | 3.13E-20 | -0.410784894 | 0.594 | 0.702 | 7.38E-16 |
| GBP1 | 1.47E-13 | -0.407186373 | 0.214 | 0.327 | 3.46E-09 |
| NBL1 | 2.22E-15 | -0.405568462 | 0.249 | 0.368 | 5.23E-11 |
| ODF3B | 1.36E-17 | -0.399362668 | 0.135 | 0.261 | 3.20E-13 |
| POLE4 | 7.30E-20 | -0.399186328 | 0.4 | 0.535 | 1.72E-15 |
| ITM2A | 4.75E-16 | -0.39874135 | 0.553 | 0.692 | 1.12E-11 |
| LGALS3 | 2.60E-23 | -0.398068368 | 0.896 | 0.925 | 6.13E-19 |
| SDCBP | 1.26E-21 | -0.393196782 | 0.733 | 0.817 | 2.98E-17 |
| APOLD1 | 5.41E-16 | -0.392965003 | 0.144 | 0.272 | 1.28E-11 |
| TMEM273 | 6.12E-18 | -0.389754333 | 0.144 | 0.271 | 1.44E-13 |
| IFIT3 | 1.81E-11 | -0.385095727 | 0.179 | 0.282 | 4.27E-07 |
| ARID5B | 1.98E-12 | -0.381963759 | 0.541 | 0.626 | 4.66E-08 |
| RASSF9 | 5.75E-13 | -0.376536619 | 0.212 | 0.325 | 1.36E-08 |
| C21orf91 | 9.62E-16 | -0.37378639 | 0.148 | 0.266 | 2.27E-11 |
| PLVAP | 1.41E-19 | -0.372428755 | 0.661 | 0.779 | 3.33E-15 |
| PLAC9 | 6.22E-16 | -0.368621065 | 0.174 | 0.307 | 1.47E-11 |
| CALCOCO2 | 9.64E-14 | -0.368515811 | 0.452 | 0.555 | 2.28E-09 |
| HLA-F | 2.14E-13 | -0.367382486 | 0.504 | 0.581 | 5.04E-09 |
| IL33 | 2.85E-11 | -0.364502687 | 0.473 | 0.558 | 6.74E-07 |
| GBP2 | 1.16E-11 | -0.362439016 | 0.257 | 0.353 | 2.73E-07 |
| HSD17B11 | 6.01E-15 | -0.362345742 | 0.338 | 0.452 | 1.42E-10 |
| CRIP1 | 1.36E-07 | -0.362257414 | 0.187 | 0.263 | 0.003200653 |
| EMCN | 1.07E-11 | -0.361778595 | 0.471 | 0.584 | 2.53E-07 |
| TGFBR2 | 8.24E-14 | -0.360724916 | 0.77 | 0.829 | 1.95E-09 |
| MALL | 6.35E-12 | -0.357466062 | 0.325 | 0.436 | 1.50E-07 |
| LDHA | 4.89E-20 | -0.35724727 | 0.808 | 0.843 | 1.15E-15 |
| TPD52L1 | 3.15E-11 | -0.354814552 | 0.273 | 0.365 | 7.43E-07 |
| NR2F2 | 2.13E-14 | -0.354209905 | 0.456 | 0.574 | 5.02E-10 |
| BTN3A2 | 5.28E-15 | -0.352494249 | 0.174 | 0.294 | 1.25E-10 |
| KLF9 | 9.49E-14 | -0.352124648 | 0.317 | 0.428 | 2.24E-09 |
| NRN1 | 3.66E-11 | -0.350675983 | 0.513 | 0.6 | 8.65E-07 |
| ITGB4 | 1.86E-14 | -0.348647975 | 0.277 | 0.405 | 4.40E-10 |
| TFPI | 8.06E-12 | -0.348167906 | 0.55 | 0.643 | 1.90E-07 |
| WNK1 | 5.54E-14 | -0.342881092 | 0.246 | 0.364 | 1.31E-09 |
| ZFP36L2 | 3.31E-11 | -0.340376193 | 0.771 | 0.822 | 7.81E-07 |
| ZNF385D | 7.66E-12 | -0.337604021 | 0.234 | 0.344 | 1.81E-07 |
| CLEC2B | 8.41E-10 | -0.335244435 | 0.381 | 0.469 | 1.99E-05 |
| LRRFIP1 | 8.46E-17 | -0.335126523 | 0.804 | 0.853 | 2.00E-12 |
| TACC1 | 3.67E-18 | -0.334927741 | 0.632 | 0.735 | 8.65E-14 |
| ADAM15 | 2.54E-14 | -0.333371058 | 0.455 | 0.577 | 5.98E-10 |
| NAMPT | 2.85E-09 | -0.331063598 | 0.459 | 0.531 | 6.73E-05 |
| EPS8 | 7.85E-16 | -0.329603837 | 0.217 | 0.351 | 1.85E-11 |
| IFI16 | 1.37E-15 | -0.328321389 | 0.607 | 0.71 | 3.23E-11 |
| TNXB | 3.80E-13 | -0.327948677 | 0.148 | 0.264 | 8.98E-09 |
| RARRES3 | 4.99E-09 | -0.327535493 | 0.294 | 0.382 | 0.0001179 |
| DUSP23 | 9.30E-12 | -0.327084529 | 0.542 | 0.633 | 2.20E-07 |
| HLA-E | 1.78E-27 | -0.326829697 | 0.961 | 0.983 | 4.21E-23 |
| TSC22D3 | 5.74E-09 | -0.325679391 | 0.551 | 0.633 | 0.000135437 |
| LAP3 | 2.72E-09 | -0.32443373 | 0.539 | 0.615 | 6.41E-05 |
| CAVIN2 | 7.49E-08 | -0.323341478 | 0.636 | 0.69 | 0.00176841 |
| KANK3 | 9.48E-12 | -0.320000085 | 0.352 | 0.462 | 2.24E-07 |
| NET1 | 1.27E-09 | -0.318520353 | 0.214 | 0.304 | 3.00E-05 |
| PRCP | 1.47E-22 | -0.318119895 | 0.462 | 0.663 | 3.46E-18 |
| PMP22 | 2.18E-10 | -0.316560577 | 0.322 | 0.425 | 5.14E-06 |
| HSPD1 | 6.08E-14 | -0.315943349 | 0.55 | 0.649 | 1.44E-09 |
| NASP | 2.29E-11 | -0.308986962 | 0.495 | 0.579 | 5.41E-07 |
| ITM2B | 5.84E-25 | -0.308157513 | 0.987 | 0.99 | 1.38E-20 |
| CYYR1 | 5.20E-13 | -0.307721632 | 0.419 | 0.532 | 1.23E-08 |
| WARS | 9.26E-06 | -0.307366026 | 0.24 | 0.3 | 0.218498504 |
| CCDC69 | 2.47E-11 | -0.307132918 | 0.205 | 0.306 | 5.82E-07 |
| LY6E | 3.58E-09 | -0.306343077 | 0.681 | 0.722 | 8.45E-05 |
| CXorf36 | 7.46E-10 | -0.30045359 | 0.264 | 0.359 | 1.76E-05 |
| EPHX1 | 3.28E-09 | -0.29642142 | 0.326 | 0.41 | 7.75E-05 |
| TMOD3 | 2.48E-08 | -0.293285633 | 0.647 | 0.688 | 0.000586399 |
| RAB11A | 3.32E-13 | -0.292083037 | 0.646 | 0.707 | 7.84E-09 |
| TAGLN2 | 2.64E-21 | -0.291183416 | 0.918 | 0.944 | 6.24E-17 |
| HMGB2 | 3.65E-09 | -0.289016366 | 0.356 | 0.438 | 8.62E-05 |
| SAMHD1 | 4.78E-10 | -0.288394499 | 0.171 | 0.259 | 1.13E-05 |
| KLF2 | 1.17E-16 | -0.284725045 | 0.856 | 0.918 | 2.77E-12 |
| CDKN1A | 8.36E-07 | -0.28439253 | 0.341 | 0.413 | 0.019728809 |
| POSTN | 5.95E-13 | -0.283556776 | 0.233 | 0.369 | 1.41E-08 |
| TPM3 | 2.14E-15 | -0.283035849 | 0.829 | 0.875 | 5.04E-11 |
| IL1R1 | 3.26E-11 | -0.282434357 | 0.325 | 0.436 | 7.69E-07 |
| LPAR6 | 2.80E-08 | -0.281307615 | 0.389 | 0.469 | 0.000660499 |
| CFLAR | 1.83E-11 | -0.280775942 | 0.411 | 0.514 | 4.33E-07 |
| HYAL2 | 0.00096028 | -0.279541872 | 0.746 | 0.766 | 1 |
| TNFSF10 | 0.000562888 | -0.278393586 | 0.778 | 0.778 | 1 |
| NEDD9 | 6.01E-07 | -0.276502836 | 0.259 | 0.334 | 0.014197504 |
| TGFBR3 | 8.59E-08 | -0.276120178 | 0.325 | 0.405 | 0.002027886 |
| H3F3B | 2.75E-20 | -0.275437051 | 0.982 | 0.987 | 6.49E-16 |
| ANP32B | 8.63E-11 | -0.275019593 | 0.609 | 0.676 | 2.04E-06 |
| LMCD1 | 4.76E-12 | -0.27498644 | 0.532 | 0.633 | 1.12E-07 |
| IFNGR1 | 6.86E-10 | -0.273107729 | 0.43 | 0.523 | 1.62E-05 |
| KLF4 | 1.03E-10 | -0.272573425 | 0.523 | 0.626 | 2.42E-06 |
| AHNAK | 1.49E-09 | -0.27227739 | 0.634 | 0.711 | 3.52E-05 |
| GIMAP6 | 4.24E-10 | -0.272129043 | 0.232 | 0.325 | 1.00E-05 |
| SYPL1 | 2.32E-10 | -0.272107871 | 0.521 | 0.606 | 5.47E-06 |
| EIF2S3 | 2.41E-09 | -0.271143466 | 0.309 | 0.397 | 5.68E-05 |
| JUNB | 1.01E-07 | -0.268404492 | 0.914 | 0.932 | 0.00239188 |
| HIPK3 | 3.46E-08 | -0.268195037 | 0.328 | 0.409 | 0.000815786 |
| MAP3K8 | 5.65E-08 | -0.266358162 | 0.254 | 0.333 | 0.001334609 |
| IFITM2 | 1.46E-17 | -0.265045948 | 0.942 | 0.963 | 3.45E-13 |
| RCN1 | 1.34E-07 | -0.263850984 | 0.388 | 0.458 | 0.003160832 |
| SERPINB1 | 7.05E-07 | -0.263231533 | 0.541 | 0.599 | 0.016635159 |
| IL3RA | 1.33E-07 | -0.258559298 | 0.346 | 0.419 | 0.00314788 |
| NFKBIZ | 7.71E-05 | -0.258236912 | 0.32 | 0.374 | 1 |
| SFPQ | 8.47E-09 | -0.257755138 | 0.545 | 0.615 | 0.000199876 |
| CAST | 1.71E-12 | -0.254652803 | 0.803 | 0.839 | 4.03E-08 |
| ATP1A1 | 1.15E-08 | -0.253022486 | 0.523 | 0.592 | 0.000272345 |
| GSTK1 | 6.74E-08 | -0.252432341 | 0.587 | 0.634 | 0.001591363 |
| C1orf115 | 1.40E-09 | -0.252256121 | 0.162 | 0.252 | 3.31E-05 |
| MKL2 | 8.40E-07 | -0.251365615 | 0.297 | 0.375 | 0.019834088 |
| CSRNP1 | 3.74E-07 | -0.251142254 | 0.259 | 0.337 | 0.008832022 |
| PGM5 | 1.77E-12 | -0.25011392 | 0.163 | 0.274 | 4.18E-08 |
| DNAJA1 | 1.98E-08 | -0.250039202 | 0.78 | 0.805 | 0.000466191 |
| SOX18 | 5.70E-10 | 0.250427502 | 0.815 | 0.77 | 1.35E-05 |
| SEC61B | 2.54E-13 | 0.250896055 | 0.839 | 0.781 | 5.99E-09 |
| PLEKHO1 | 1.53E-13 | 0.250950721 | 0.267 | 0.165 | 3.62E-09 |
| S100A13 | 3.12E-11 | 0.25264338 | 0.844 | 0.776 | 7.35E-07 |
| PAWR | 9.96E-10 | 0.253439241 | 0.265 | 0.18 | 2.35E-05 |
| MYDGF | 3.92E-10 | 0.253874613 | 0.669 | 0.574 | 9.26E-06 |
| NAA38 | 8.40E-12 | 0.253916959 | 0.659 | 0.553 | 1.98E-07 |
| GSTP1 | 6.89E-09 | 0.256112975 | 0.911 | 0.91 | 0.000162662 |
| BEX3 | 3.43E-09 | 0.256321151 | 0.623 | 0.535 | 8.11E-05 |
| SOX4 | 0.000201778 | 0.256389365 | 0.576 | 0.511 | 1 |
| FHL1 | 2.70E-10 | 0.257870466 | 0.377 | 0.278 | 6.37E-06 |
| IGFBP7 | 6.87E-14 | 0.261523587 | 0.967 | 0.966 | 1.62E-09 |
| S100A6 | 8.89E-18 | 0.261628227 | 0.987 | 0.983 | 2.10E-13 |
| LAMB1 | 5.10E-13 | 0.264974182 | 0.468 | 0.338 | 1.20E-08 |
| LDHB | 1.31E-08 | 0.26550818 | 0.668 | 0.605 | 0.000310289 |
| CALD1 | 6.36E-15 | 0.26629861 | 0.809 | 0.684 | 1.50E-10 |
| PGLS | 3.33E-13 | 0.267151129 | 0.61 | 0.497 | 7.85E-09 |
| SGCE | 5.43E-21 | 0.269078855 | 0.251 | 0.129 | 1.28E-16 |
| C4orf48 | 1.64E-20 | 0.269500372 | 0.406 | 0.251 | 3.86E-16 |
| ITGB1 | 1.61E-12 | 0.272213361 | 0.831 | 0.771 | 3.81E-08 |
| SRGN | 6.79E-14 | 0.274850029 | 0.784 | 0.672 | 1.60E-09 |
| MZT2A | 4.85E-15 | 0.275713617 | 0.481 | 0.342 | 1.14E-10 |
| BNIP3L | 5.69E-13 | 0.276387417 | 0.537 | 0.42 | 1.34E-08 |
| ACKR3 | 1.94E-09 | 0.27743948 | 0.267 | 0.182 | 4.58E-05 |
| MEDAG | 5.87E-15 | 0.277787517 | 0.254 | 0.15 | 1.39E-10 |
| RAB34 | 1.30E-21 | 0.281242402 | 0.29 | 0.158 | 3.06E-17 |
| LIMCH1 | 6.94E-13 | 0.285300316 | 0.488 | 0.361 | 1.64E-08 |
| COL15A1 | 0.180879149 | 0.28532987 | 0.254 | 0.295 | 1 |
| LAMA4 | 5.19E-08 | 0.286151245 | 0.461 | 0.371 | 0.001225443 |
| SSR4 | 1.71E-14 | 0.286331608 | 0.891 | 0.84 | 4.04E-10 |
| RBP1 | 2.02E-14 | 0.289142544 | 0.34 | 0.225 | 4.77E-10 |
| BST2 | 1.90E-13 | 0.289441301 | 0.817 | 0.756 | 4.48E-09 |
| RHOB | 2.62E-18 | 0.293656519 | 0.733 | 0.577 | 6.19E-14 |
| METRNL | 1.02E-05 | 0.300118014 | 0.555 | 0.505 | 0.240054063 |
| RPS10 | 5.54E-08 | 0.300209541 | 0.852 | 0.802 | 0.001307509 |
| HTRA1 | 2.08E-11 | 0.301283164 | 0.605 | 0.491 | 4.90E-07 |
| RFLNB | 5.86E-17 | 0.302380235 | 0.27 | 0.154 | 1.38E-12 |
| PKIG | 4.04E-10 | 0.302384311 | 0.531 | 0.434 | 9.53E-06 |
| TMEM120A | 1.33E-12 | 0.303598562 | 0.387 | 0.277 | 3.15E-08 |
| LTBP2 | 4.65E-19 | 0.304130816 | 0.328 | 0.191 | 1.10E-14 |
| CCDC85B | 4.72E-22 | 0.306075079 | 0.94 | 0.911 | 1.11E-17 |
| GJA4 | 6.27E-16 | 0.3067032 | 0.339 | 0.213 | 1.48E-11 |
| CHCHD10 | 3.97E-20 | 0.310982554 | 0.368 | 0.23 | 9.36E-16 |
| SAT1 | 0.000231622 | 0.312846385 | 0.747 | 0.679 | 1 |
| ALDH1A1 | 6.70E-27 | 0.320145085 | 0.278 | 0.136 | 1.58E-22 |
| OAZ1 | 2.21E-21 | 0.322585164 | 0.911 | 0.868 | 5.22E-17 |
| BMP4 | 8.02E-15 | 0.324189894 | 0.364 | 0.239 | 1.89E-10 |
| FAM107B | 1.72E-18 | 0.325825252 | 0.483 | 0.329 | 4.06E-14 |
| CRIM1 | 4.20E-07 | 0.330214211 | 0.595 | 0.532 | 0.009906197 |
| TNFRSF12A | 2.08E-13 | 0.337677077 | 0.274 | 0.172 | 4.92E-09 |
| C12orf57 | 7.31E-17 | 0.341009895 | 0.705 | 0.605 | 1.72E-12 |
| MYL9 | 4.18E-15 | 0.343159576 | 0.486 | 0.358 | 9.86E-11 |
| PCDH17 | 6.82E-17 | 0.34895448 | 0.411 | 0.279 | 1.61E-12 |
| HSP90B1 | 8.40E-20 | 0.352805267 | 0.928 | 0.886 | 1.98E-15 |
| GPX4 | 3.77E-20 | 0.355150488 | 0.859 | 0.788 | 8.89E-16 |
| PMEPA1 | 8.45E-19 | 0.355443737 | 0.395 | 0.259 | 1.99E-14 |
| TIMP1 | 9.05E-21 | 0.357142481 | 0.971 | 0.943 | 2.14E-16 |
| RALA | 4.77E-11 | 0.358797149 | 0.431 | 0.326 | 1.13E-06 |
| WWTR1 | 3.93E-18 | 0.365418026 | 0.726 | 0.624 | 9.28E-14 |
| MYL12B | 3.67E-28 | 0.36956157 | 0.973 | 0.953 | 8.67E-24 |
| FILIP1 | 6.91E-24 | 0.370648685 | 0.462 | 0.289 | 1.63E-19 |
| RFK | 1.35E-15 | 0.373159484 | 0.426 | 0.299 | 3.19E-11 |
| TPM2 | 3.41E-25 | 0.380736812 | 0.331 | 0.182 | 8.05E-21 |
| FBLIM1 | 4.42E-28 | 0.384044529 | 0.451 | 0.263 | 1.04E-23 |
| CYBA | 4.69E-28 | 0.388000749 | 0.597 | 0.408 | 1.11E-23 |
| HOMER3 | 1.94E-20 | 0.388456402 | 0.381 | 0.241 | 4.57E-16 |
| MTRNR2L8 | 0.041350569 | 0.392322403 | 0.433 | 0.485 | 1 |
| SELL | 1.37E-15 | 0.399671182 | 0.29 | 0.179 | 3.23E-11 |
| LXN | 2.11E-06 | 0.402726001 | 0.436 | 0.369 | 0.049718822 |
| OMD | 8.32E-21 | 0.408167691 | 0.402 | 0.244 | 1.96E-16 |
| TUBA1A | 1.54E-09 | 0.416232657 | 0.721 | 0.642 | 3.64E-05 |
| AC004540.2 | 2.46E-26 | 0.419206552 | 0.259 | 0.127 | 5.80E-22 |
| FTL | 4.37E-27 | 0.433570355 | 0.996 | 0.99 | 1.03E-22 |
| BMX | 1.78E-23 | 0.452450501 | 0.516 | 0.347 | 4.21E-19 |
| IGFBP3 | 6.09E-24 | 0.455263408 | 0.397 | 0.231 | 1.44E-19 |
| BGN | 5.11E-46 | 0.457201046 | 0.689 | 0.393 | 1.21E-41 |
| TAGLN | 1.11E-08 | 0.461704852 | 0.303 | 0.219 | 0.000260951 |
| RGS3 | 3.93E-19 | 0.462188799 | 0.411 | 0.273 | 9.27E-15 |
| COL8A1 | 5.50E-20 | 0.46670074 | 0.418 | 0.271 | 1.30E-15 |
| CPE | 4.10E-25 | 0.494400397 | 0.437 | 0.265 | 9.67E-21 |
| RGCC | 3.76E-26 | 0.503492674 | 0.397 | 0.227 | 8.86E-22 |
| OGN | 2.90E-22 | 0.504546658 | 0.382 | 0.225 | 6.84E-18 |
| FN1 | 4.15E-38 | 0.511238601 | 0.622 | 0.368 | 9.80E-34 |
| EDN1 | 3.39E-22 | 0.519182157 | 0.523 | 0.353 | 7.99E-18 |
| COL4A2 | 3.05E-15 | 0.52925936 | 0.413 | 0.29 | 7.21E-11 |
| THBS1 | 8.75E-37 | 0.532566942 | 0.376 | 0.188 | 2.06E-32 |
| FTH1 | 7.38E-15 | 0.559154239 | 0.996 | 0.994 | 1.74E-10 |
| LGALS1 | 3.07E-42 | 0.601282 | 0.789 | 0.554 | 7.25E-38 |
| EFEMP1 | 9.51E-34 | 0.623402209 | 0.523 | 0.313 | 2.25E-29 |
| CTGF | 4.99E-21 | 0.624521412 | 0.531 | 0.38 | 1.18E-16 |
| COL4A1 | 8.75E-15 | 0.627746594 | 0.42 | 0.303 | 2.07E-10 |
| PLCG2 | 0.095112782 | 0.631333768 | 0.621 | 0.604 | 1 |
| SERPINE1 | 3.21E-30 | 0.680404618 | 0.387 | 0.215 | 7.58E-26 |
| PROCR | 4.47E-23 | 0.703645077 | 0.613 | 0.458 | 1.05E-18 |
| S100A4 | 2.71E-26 | 0.726125586 | 0.5 | 0.32 | 6.40E-22 |
| MTRNR2L12 | 0.396018001 | 0.744495237 | 0.784 | 0.821 | 1 |
| CYR61 | 3.00E-32 | 0.806459091 | 0.543 | 0.362 | 7.08E-28 |
| IGKC | 8.94E-29 | 0.885519072 | 0.488 | 0.297 | 2.11E-24 |
| ITLN1 | 3.86E-25 | 1.040277295 | 0.372 | 0.218 | 9.12E-21 |
| MGP | 9.89E-33 | 1.183466285 | 0.928 | 0.894 | 2.33E-28 |

**Supplementary Table 3**. DEGs in VSMCs within atherosclerotic plaque tissue

| Gene | p_val | avg_log2FC | Percentage of cells expressing genes in Control | Percentage of cells expressing genes in AS | p_val_adj |
| --- | --- | --- | --- | --- | --- |
| HSPB7 | 0 | 0.988293916 | 0.604 | 0.211 | 0 |
| CSRP1 | 0 | 0.963735481 | 0.898 | 0.723 | 0 |
| RAMP1 | 0 | 1.015806281 | 0.823 | 0.504 | 0 |
| SUSD5 | 0 | 0.910559685 | 0.568 | 0.202 | 0 |
| MTRNR2L12 | 0 | 2.097913015 | 0.878 | 0.705 | 0 |
| TRIM36 | 0 | 0.560154858 | 0.294 | 0.026 | 0 |
| RPS10 | 0 | 1.260232749 | 0.896 | 0.724 | 0 |
| FLNA | 0 | 1.156593244 | 0.865 | 0.72 | 0 |
| TPM2 | 0 | 1.305891752 | 0.973 | 0.876 | 0 |
| TPH1 | 0 | 0.566130044 | 0.338 | 0.078 | 0 |
| ITGB1 | 0 | 0.991668642 | 0.94 | 0.86 | 0 |
| STK38L | 0 | 1.128832743 | 0.661 | 0.263 | 0 |
| SAMD4A | 0 | 0.583058345 | 0.364 | 0.074 | 0 |
| RTN1 | 0 | 1.228305883 | 0.483 | 0.056 | 0 |
| FOS | 0 | -1.386526531 | 0.553 | 0.82 | 0 |
| ADSSL1 | 0 | 0.527872608 | 0.286 | 0.052 | 0 |
| CRIP1 | 0 | 1.349340158 | 0.78 | 0.48 | 0 |
| B2M | 0 | -0.662517181 | 0.988 | 0.983 | 0 |
| TNFRSF12A | 0 | 1.181106173 | 0.727 | 0.393 | 0 |
| PLCG2 | 0 | 1.602061588 | 0.792 | 0.413 | 0 |
| MYOCD | 0 | 0.713628072 | 0.406 | 0.105 | 0 |
| SGCA | 0 | 0.837976536 | 0.723 | 0.35 | 0 |
| DSTN | 0 | 0.815445183 | 0.979 | 0.95 | 0 |
| RPS21 | 0 | 0.816099187 | 0.951 | 0.888 | 0 |
| CNN1 | 0 | 1.254281824 | 0.698 | 0.367 | 0 |
| PPP1R14A | 0 | 0.999821061 | 0.868 | 0.678 | 0 |
| RPL13A | 0 | -0.91100572 | 0.955 | 0.939 | 0 |
| MT-ND3 | 0 | 1.075720984 | 0.972 | 0.958 | 0 |
| MFGE8 | 6.83E-308 | 0.791460128 | 0.931 | 0.818 | 1.61E-303 |
| FAM198B | 5.51E-307 | 0.571845261 | 0.489 | 0.169 | 1.30E-302 |
| SMTN | 4.90E-305 | 0.671115612 | 0.477 | 0.164 | 1.16E-300 |
| HIST1H4C | 8.00E-304 | 0.866767263 | 0.777 | 0.501 | 1.89E-299 |
| ALDH1B1 | 9.48E-300 | 0.643572604 | 0.465 | 0.157 | 2.24E-295 |
| MTRNR2L8 | 5.04E-299 | 1.489609791 | 0.643 | 0.372 | 1.19E-294 |
| RPS20 | 5.13E-298 | -1.025585783 | 0.751 | 0.827 | 1.21E-293 |
| HLA-A | 1.94E-297 | -0.851716215 | 0.8 | 0.881 | 4.58E-293 |
| PPP1R12B | 2.12E-297 | 0.962067485 | 0.716 | 0.436 | 5.01E-293 |
| MT-ATP8 | 4.51E-296 | 0.804693182 | 0.65 | 0.331 | 1.06E-291 |
| IGFBP2 | 7.97E-296 | 0.896896857 | 0.929 | 0.685 | 1.88E-291 |
| HLA-B | 7.02E-294 | -0.879600006 | 0.598 | 0.839 | 1.66E-289 |
| RPL36A | 4.16E-287 | 0.760554207 | 0.751 | 0.476 | 9.82E-283 |
| GRIA2 | 1.81E-278 | 0.553900216 | 0.365 | 0.098 | 4.27E-274 |
| NEXN | 6.23E-278 | 0.8831315 | 0.812 | 0.576 | 1.47E-273 |
| C5orf46 | 1.21E-277 | 0.754605627 | 0.265 | 0.053 | 2.85E-273 |
| ACTN1 | 3.13E-276 | 0.810019968 | 0.831 | 0.6 | 7.38E-272 |
| ARID5A | 3.01E-273 | 0.651811277 | 0.544 | 0.232 | 7.10E-269 |
| PPDPF | 7.66E-270 | 0.653474336 | 0.906 | 0.748 | 1.81E-265 |
| PDE4DIP | 1.61E-269 | 0.651917141 | 0.571 | 0.256 | 3.80E-265 |
| MYEF2 | 1.12E-267 | 0.454330419 | 0.373 | 0.107 | 2.64E-263 |
| HACD1 | 2.02E-262 | 0.558202184 | 0.481 | 0.181 | 4.76E-258 |
| TC2N | 1.18E-260 | 0.480497288 | 0.304 | 0.074 | 2.79E-256 |
| SCX | 2.76E-260 | 0.601967286 | 0.35 | 0.099 | 6.51E-256 |
| CRIM1 | 2.93E-260 | 0.765630444 | 0.715 | 0.424 | 6.93E-256 |
| FIBIN | 6.95E-260 | 0.774224432 | 0.612 | 0.297 | 1.64E-255 |
| MRPL33 | 3.39E-256 | 0.716876521 | 0.784 | 0.552 | 8.00E-252 |
| MCAM | 2.69E-254 | 0.903413281 | 0.691 | 0.409 | 6.34E-250 |
| PDLIM7 | 8.43E-254 | 0.700653265 | 0.778 | 0.508 | 1.99E-249 |
| CEBPD | 3.60E-253 | -1.450801481 | 0.453 | 0.695 | 8.50E-249 |
| TAGLN | 6.91E-253 | 0.692124064 | 0.981 | 0.92 | 1.63E-248 |
| CST3 | 7.07E-246 | -0.84628892 | 0.972 | 0.962 | 1.67E-241 |
| ZNF385D | 7.22E-245 | 0.498991337 | 0.459 | 0.167 | 1.70E-240 |
| NOV | 1.02E-243 | 0.804006216 | 0.811 | 0.554 | 2.40E-239 |
| NDUFA13 | 6.44E-241 | 0.569334076 | 0.739 | 0.45 | 1.52E-236 |
| PMEPA1 | 1.69E-240 | 0.671436779 | 0.805 | 0.547 | 3.98E-236 |
| RPL36 | 1.47E-239 | 0.548932325 | 0.956 | 0.918 | 3.46E-235 |
| ATP5F1E | 2.21E-238 | 0.482817105 | 0.959 | 0.899 | 5.22E-234 |
| NPNT | 2.29E-236 | 0.483500376 | 0.431 | 0.152 | 5.40E-232 |
| JUN | 3.41E-230 | -1.436283515 | 0.748 | 0.817 | 8.05E-226 |
| SPINT2 | 3.92E-229 | 0.63699778 | 0.777 | 0.473 | 9.26E-225 |
| ACTN4 | 1.17E-228 | 0.68078875 | 0.826 | 0.605 | 2.76E-224 |
| MYH10 | 1.40E-227 | 0.763508432 | 0.849 | 0.59 | 3.30E-223 |
| SCRG1 | 3.96E-226 | 0.549489207 | 0.559 | 0.25 | 9.35E-222 |
| C1R | 8.13E-225 | -1.316117309 | 0.543 | 0.74 | 1.92E-220 |
| MEF2A | 3.32E-221 | 0.599509411 | 0.649 | 0.359 | 7.84E-217 |
| CD44 | 1.81E-220 | 0.605416822 | 0.661 | 0.37 | 4.26E-216 |
| SUGCT | 7.98E-220 | 0.604060606 | 0.61 | 0.31 | 1.88E-215 |
| HAND2 | 5.91E-218 | 0.372764091 | 0.365 | 0.118 | 1.39E-213 |
| AOC3 | 4.86E-217 | 0.497506484 | 0.533 | 0.242 | 1.15E-212 |
| SLC25A4 | 3.15E-215 | 0.628491092 | 0.786 | 0.534 | 7.44E-211 |
| KALRN | 2.20E-213 | 0.425539738 | 0.407 | 0.147 | 5.19E-209 |
| HLA-C | 3.92E-211 | -0.663843462 | 0.84 | 0.886 | 9.25E-207 |
| DUSP26 | 4.35E-211 | 0.395589341 | 0.363 | 0.121 | 1.03E-206 |
| MT-CO1 | 1.64E-210 | -0.54635836 | 0.99 | 0.976 | 3.87E-206 |
| ATP5ME | 3.95E-207 | 0.553231306 | 0.838 | 0.664 | 9.33E-203 |
| PLEKHO1 | 5.13E-207 | 0.514117851 | 0.529 | 0.248 | 1.21E-202 |
| COX7A1 | 1.11E-206 | 0.561849727 | 0.857 | 0.678 | 2.62E-202 |
| ITIH5 | 4.45E-206 | 0.466774218 | 0.403 | 0.151 | 1.05E-201 |
| HSPB8 | 1.59E-205 | 0.565012611 | 0.476 | 0.212 | 3.75E-201 |
| SORT1 | 5.51E-205 | 0.474485903 | 0.509 | 0.23 | 1.30E-200 |
| SLC22A3 | 1.63E-201 | 0.353276453 | 0.305 | 0.09 | 3.85E-197 |
| FMOD | 7.82E-201 | 0.563365405 | 0.567 | 0.286 | 1.85E-196 |
| NEDD9 | 1.17E-200 | 0.447916453 | 0.362 | 0.128 | 2.76E-196 |
| PET100 | 9.06E-199 | 0.474806645 | 0.643 | 0.358 | 2.14E-194 |
| MYL6 | 1.84E-198 | 0.448315087 | 0.984 | 0.954 | 4.34E-194 |
| ITM2B | 2.13E-197 | -0.629244139 | 0.959 | 0.949 | 5.04E-193 |
| CTHRC1 | 8.26E-196 | 0.497602042 | 0.527 | 0.241 | 1.95E-191 |
| RPS27 | 1.55E-195 | 0.51549327 | 0.976 | 0.962 | 3.66E-191 |
| TNS1 | 6.13E-195 | 0.604186957 | 0.683 | 0.415 | 1.45E-190 |
| GOLGA8A | 1.19E-194 | 0.36718875 | 0.317 | 0.1 | 2.81E-190 |
| LPP | 1.43E-194 | 0.697987264 | 0.822 | 0.654 | 3.36E-190 |
| MYO1D | 1.21E-193 | 0.532503333 | 0.598 | 0.316 | 2.85E-189 |
| RNF115 | 3.07E-193 | 0.635647583 | 0.589 | 0.332 | 7.25E-189 |
| CSPG4 | 4.24E-193 | 0.417971089 | 0.368 | 0.133 | 1.00E-188 |
| PLAC9 | 5.35E-193 | -0.856223082 | 0.508 | 0.729 | 1.26E-188 |
| FOSB | 1.78E-192 | -1.206982794 | 0.23 | 0.506 | 4.20E-188 |
| RPLP0 | 6.82E-191 | -0.601404148 | 0.913 | 0.9 | 1.61E-186 |
| JUNB | 6.95E-190 | -1.253073459 | 0.636 | 0.767 | 1.64E-185 |
| ARHGAP24 | 7.48E-188 | 0.379385435 | 0.331 | 0.113 | 1.77E-183 |
| PRUNE2 | 1.37E-187 | 0.368392094 | 0.505 | 0.223 | 3.23E-183 |
| MRAP2 | 1.37E-187 | 0.39529377 | 0.398 | 0.154 | 3.24E-183 |
| YAP1 | 4.32E-187 | 0.47316888 | 0.513 | 0.247 | 1.02E-182 |
| NPR1 | 1.50E-186 | 0.346755361 | 0.363 | 0.13 | 3.55E-182 |
| MYLK | 1.26E-185 | 0.567832507 | 0.778 | 0.527 | 2.97E-181 |
| ST6GAL2 | 8.70E-185 | 0.355039446 | 0.301 | 0.094 | 2.05E-180 |
| RPS29 | 6.96E-184 | 0.494592201 | 0.941 | 0.894 | 1.64E-179 |
| RGS5 | 9.22E-184 | 0.619470144 | 0.83 | 0.613 | 2.18E-179 |
| SGCD | 7.21E-183 | 0.367171754 | 0.371 | 0.14 | 1.70E-178 |
| MGST3 | 3.52E-182 | 0.475549263 | 0.945 | 0.888 | 8.30E-178 |
| IFITM2 | 8.60E-182 | -0.642097405 | 0.68 | 0.825 | 2.03E-177 |
| MAN2A1 | 1.43E-181 | 0.313832459 | 0.295 | 0.092 | 3.38E-177 |
| MYH11 | 3.79E-181 | 0.619681526 | 0.834 | 0.633 | 8.94E-177 |
| ATP5MD | 2.12E-180 | 0.488557244 | 0.849 | 0.675 | 4.99E-176 |
| LAMA5 | 3.30E-179 | 0.317624892 | 0.292 | 0.091 | 7.80E-175 |
| IGFBP5 | 3.24E-178 | -1.111286209 | 0.731 | 0.813 | 7.64E-174 |
| GALNT18 | 2.63E-177 | 0.30004058 | 0.273 | 0.082 | 6.20E-173 |
| PCDH7 | 1.19E-176 | 0.479342378 | 0.69 | 0.416 | 2.80E-172 |
| SNRPN | 6.10E-174 | 0.506628739 | 0.767 | 0.525 | 1.44E-169 |
| MYLIP | 1.67E-172 | 0.48574476 | 0.463 | 0.216 | 3.93E-168 |
| UQCR11 | 6.47E-172 | 0.471872518 | 0.846 | 0.674 | 1.53E-167 |
| TTLL7 | 4.40E-171 | 0.511634902 | 0.635 | 0.37 | 1.04E-166 |
| SLIRP | 5.27E-169 | 0.479364827 | 0.713 | 0.478 | 1.24E-164 |
| IFITM3 | 5.27E-169 | -0.468493791 | 0.959 | 0.949 | 1.24E-164 |
| MRVI1 | 3.83E-168 | 0.494209048 | 0.559 | 0.295 | 9.03E-164 |
| ACTC1 | 4.69E-168 | 0.996879922 | 0.334 | 0.126 | 1.11E-163 |
| PPP1CB | 5.16E-166 | 0.518192511 | 0.864 | 0.706 | 1.22E-161 |
| MT2A | 5.90E-166 | -1.041278591 | 0.648 | 0.766 | 1.39E-161 |
| ACTG2 | 1.36E-165 | 0.660452255 | 0.468 | 0.218 | 3.21E-161 |
| COX7C | 1.07E-164 | 0.410391545 | 0.947 | 0.881 | 2.53E-160 |
| MT-ND4L | 4.52E-164 | 0.616520303 | 0.859 | 0.776 | 1.07E-159 |
| FBLN1 | 1.01E-162 | -2.479880868 | 0.126 | 0.351 | 2.38E-158 |
| RASSF3 | 1.27E-162 | 0.418217912 | 0.403 | 0.172 | 3.00E-158 |
| FILIP1 | 4.63E-162 | 0.583295323 | 0.598 | 0.35 | 1.09E-157 |
| FSTL3 | 1.34E-161 | 0.423339218 | 0.405 | 0.176 | 3.17E-157 |
| FILIP1L | 2.09E-161 | 0.911574712 | 0.808 | 0.665 | 4.94E-157 |
| SLMAP | 2.34E-161 | 0.536445556 | 0.533 | 0.281 | 5.53E-157 |
| MPP7 | 5.53E-161 | 0.279449996 | 0.253 | 0.076 | 1.30E-156 |
| DAPK3 | 2.15E-160 | 0.39940761 | 0.421 | 0.19 | 5.08E-156 |
| TMEM47 | 2.31E-160 | 0.560249604 | 0.685 | 0.451 | 5.45E-156 |
| ZFP36 | 1.54E-159 | -1.375879651 | 0.532 | 0.676 | 3.62E-155 |
| DYNC1I2 | 1.70E-159 | 0.465280926 | 0.764 | 0.553 | 4.02E-155 |
| MIF | 2.09E-157 | 0.521281637 | 0.759 | 0.545 | 4.94E-153 |
| DRAP1 | 2.33E-157 | 0.428555967 | 0.753 | 0.506 | 5.51E-153 |
| ILK | 7.80E-157 | 0.472980158 | 0.819 | 0.62 | 1.84E-152 |
| C5orf24 | 1.41E-155 | 0.458477943 | 0.602 | 0.354 | 3.33E-151 |
| RCAN2 | 1.54E-155 | 0.482798723 | 0.725 | 0.465 | 3.64E-151 |
| C9orf3 | 1.48E-154 | 0.494089789 | 0.809 | 0.601 | 3.49E-150 |
| TWSG1 | 4.01E-154 | 0.397097475 | 0.511 | 0.263 | 9.47E-150 |
| GAPDH | 5.81E-153 | 0.356930006 | 0.986 | 0.964 | 1.37E-148 |
| SPEG | 3.37E-152 | 0.355720866 | 0.431 | 0.192 | 7.95E-148 |
| COL4A2 | 3.42E-152 | 0.467396478 | 0.719 | 0.468 | 8.08E-148 |
| PDLIM1 | 8.52E-152 | 0.589060296 | 0.767 | 0.57 | 2.01E-147 |
| PDLIM5 | 3.34E-151 | 0.461816119 | 0.732 | 0.493 | 7.89E-147 |
| SYNPO | 1.19E-149 | 0.404508611 | 0.574 | 0.314 | 2.82E-145 |
| SORBS1 | 1.35E-149 | 0.389830996 | 0.527 | 0.272 | 3.18E-145 |
| PKD1 | 2.24E-149 | 0.362633084 | 0.384 | 0.163 | 5.28E-145 |
| UBL5 | 6.25E-149 | 0.397913731 | 0.888 | 0.763 | 1.48E-144 |
| DACT3 | 1.06E-148 | 0.378578212 | 0.433 | 0.2 | 2.50E-144 |
| RPL7 | 7.74E-148 | -0.571948377 | 0.946 | 0.92 | 1.83E-143 |
| THRB | 2.99E-147 | 0.306971546 | 0.344 | 0.138 | 7.05E-143 |
| ITGA8 | 3.70E-147 | 0.432659381 | 0.821 | 0.58 | 8.73E-143 |
| CALM1 | 2.94E-146 | 0.389888967 | 0.974 | 0.931 | 6.93E-142 |
| SLC20A2 | 1.43E-145 | 0.300558512 | 0.309 | 0.117 | 3.37E-141 |
| TES | 1.59E-145 | 0.414743096 | 0.478 | 0.239 | 3.76E-141 |
| LIMS2 | 4.04E-145 | 0.369104036 | 0.505 | 0.256 | 9.54E-141 |
| DCN | 1.32E-143 | -2.441514091 | 0.137 | 0.363 | 3.11E-139 |
| CAMK2G | 3.64E-143 | 0.3212087 | 0.408 | 0.182 | 8.58E-139 |
| TGFB1I1 | 5.06E-143 | 0.445453858 | 0.829 | 0.631 | 1.19E-138 |
| ATN1 | 1.07E-142 | 0.286667795 | 0.346 | 0.141 | 2.52E-138 |
| FERMT2 | 4.36E-142 | 0.47671079 | 0.779 | 0.579 | 1.03E-137 |
| CLIC4 | 5.32E-142 | 0.441185348 | 0.744 | 0.52 | 1.26E-137 |
| RPS2 | 1.90E-140 | -0.447546702 | 0.97 | 0.944 | 4.48E-136 |
| HMCN1 | 3.17E-139 | 0.335883744 | 0.496 | 0.249 | 7.49E-135 |
| CFH | 6.23E-139 | -1.11879654 | 0.311 | 0.537 | 1.47E-134 |
| CES1 | 7.35E-139 | 0.309692268 | 0.423 | 0.191 | 1.73E-134 |
| AP002884.1 | 9.76E-139 | 0.378520385 | 0.43 | 0.205 | 2.30E-134 |
| FOXC1 | 2.34E-138 | 0.502362822 | 0.744 | 0.533 | 5.52E-134 |
| INHBA | 1.08E-137 | 0.466596433 | 0.582 | 0.326 | 2.55E-133 |
| TPM1 | 1.42E-137 | 0.407395862 | 0.965 | 0.906 | 3.34E-133 |
| TMA7 | 1.89E-137 | 0.361253198 | 0.91 | 0.786 | 4.46E-133 |
| ENAH | 4.85E-137 | 0.515748997 | 0.65 | 0.423 | 1.14E-132 |
| NDUFB1 | 5.59E-137 | 0.414600645 | 0.789 | 0.581 | 1.32E-132 |
| CST6 | 9.72E-137 | 0.482803627 | 0.293 | 0.113 | 2.29E-132 |
| MEF2C | 1.10E-136 | 0.404688349 | 0.802 | 0.568 | 2.60E-132 |
| SORBS2 | 2.38E-136 | 0.310351479 | 0.603 | 0.333 | 5.61E-132 |
| KCNMA1 | 2.13E-135 | 0.285308597 | 0.492 | 0.242 | 5.02E-131 |
| EGR1 | 7.12E-135 | -1.034156291 | 0.39 | 0.587 | 1.68E-130 |
| SUB1 | 7.18E-135 | 0.423296704 | 0.848 | 0.68 | 1.69E-130 |
| MAOB | 1.53E-134 | 0.267845231 | 0.264 | 0.094 | 3.62E-130 |
| TBL1X | 2.78E-134 | 0.269305643 | 0.312 | 0.122 | 6.56E-130 |
| NDUFA4 | 4.22E-134 | 0.424742123 | 0.935 | 0.862 | 9.96E-130 |
| LRRFIP1 | 5.25E-134 | 0.41966992 | 0.783 | 0.545 | 1.24E-129 |
| SOD2 | 5.75E-134 | -0.800833148 | 0.103 | 0.319 | 1.36E-129 |
| RPS26 | 2.40E-133 | 0.30989931 | 0.969 | 0.934 | 5.67E-129 |
| PRRX1 | 5.42E-133 | -0.828278024 | 0.43 | 0.589 | 1.28E-128 |
| COPRS | 2.21E-132 | 0.407800091 | 0.765 | 0.53 | 5.21E-128 |
| BAG2 | 3.36E-132 | 0.527942576 | 0.463 | 0.249 | 7.93E-128 |
| NMT1 | 8.16E-132 | 0.314265908 | 0.458 | 0.229 | 1.93E-127 |
| OCIAD2 | 1.25E-131 | 0.312357282 | 0.353 | 0.152 | 2.96E-127 |
| WWP2 | 5.06E-131 | 0.259210535 | 0.279 | 0.105 | 1.19E-126 |
| PLN | 5.20E-131 | 0.392627492 | 0.754 | 0.509 | 1.23E-126 |
| C11orf96 | 1.53E-130 | 0.608384678 | 0.895 | 0.791 | 3.61E-126 |
| CRNDE | 3.59E-130 | 0.341127385 | 0.518 | 0.275 | 8.48E-126 |
| SUN1 | 1.41E-129 | 0.324045822 | 0.418 | 0.2 | 3.34E-125 |
| PRR34-AS1 | 1.61E-128 | 0.312655316 | 0.34 | 0.146 | 3.81E-124 |
| MZT2B | 1.20E-127 | 0.371634962 | 0.901 | 0.764 | 2.84E-123 |
| LHFPL6 | 1.49E-125 | -0.696228101 | 0.669 | 0.736 | 3.52E-121 |
| MXRA7 | 2.32E-125 | 0.437744442 | 0.8 | 0.634 | 5.47E-121 |
| KIF1B | 2.73E-125 | 0.293540926 | 0.306 | 0.125 | 6.43E-121 |
| EIF3J | 3.64E-125 | 0.346041667 | 0.562 | 0.322 | 8.60E-121 |
| HSPG2 | 6.55E-125 | 0.316173898 | 0.557 | 0.318 | 1.55E-120 |
| LMCD1 | 8.54E-125 | 0.431466889 | 0.72 | 0.476 | 2.02E-120 |
| GUK1 | 9.90E-125 | 0.360462102 | 0.93 | 0.821 | 2.34E-120 |
| EHBP1L1 | 2.04E-124 | 0.281163359 | 0.402 | 0.189 | 4.81E-120 |
| PDLIM4 | 3.73E-124 | 0.383362537 | 0.511 | 0.282 | 8.80E-120 |
| TXNIP | 7.64E-124 | -1.290828587 | 0.349 | 0.518 | 1.80E-119 |
| PLS3 | 3.07E-123 | 0.411688092 | 0.884 | 0.746 | 7.26E-119 |
| TGFBR1 | 4.73E-123 | 0.318843514 | 0.356 | 0.161 | 1.12E-118 |
| PRMT2 | 1.52E-122 | 0.397903012 | 0.678 | 0.455 | 3.58E-118 |
| IGFBP4 | 4.78E-122 | -0.969934474 | 0.251 | 0.444 | 1.13E-117 |
| CFL2 | 6.54E-121 | 0.432505646 | 0.615 | 0.392 | 1.54E-116 |
| PTPN11 | 1.08E-120 | 0.336754515 | 0.501 | 0.277 | 2.54E-116 |
| COX6C | 1.08E-120 | 0.349057825 | 0.909 | 0.817 | 2.55E-116 |
| ARHGEF9 | 1.90E-120 | 0.310247787 | 0.423 | 0.21 | 4.47E-116 |
| AKAP6 | 1.90E-120 | 0.281841545 | 0.277 | 0.108 | 4.49E-116 |
| KCTD10 | 1.38E-119 | 0.260880674 | 0.331 | 0.143 | 3.26E-115 |
| PSMA2 | 2.20E-118 | 0.310069029 | 0.584 | 0.346 | 5.20E-114 |
| DUSP1 | 3.14E-118 | -0.678633478 | 0.817 | 0.845 | 7.40E-114 |
| ENO1 | 4.47E-118 | 0.413618626 | 0.849 | 0.684 | 1.06E-113 |
| NDUFA1 | 6.53E-118 | 0.349553557 | 0.836 | 0.66 | 1.54E-113 |
| CPXM2 | 9.19E-118 | 0.323839037 | 0.494 | 0.268 | 2.17E-113 |
| TCEAL2 | 4.05E-117 | 0.354131524 | 0.458 | 0.233 | 9.55E-113 |
| KTN1 | 1.29E-116 | 0.352198945 | 0.86 | 0.708 | 3.04E-112 |
| PALLD | 1.44E-116 | 0.451268288 | 0.826 | 0.677 | 3.40E-112 |
| RPLP1 | 1.60E-116 | 0.25445864 | 0.992 | 0.983 | 3.78E-112 |
| PRKG1 | 2.67E-116 | 0.338565291 | 0.617 | 0.379 | 6.30E-112 |
| PFKP | 3.76E-116 | 0.273869025 | 0.305 | 0.13 | 8.88E-112 |
| SOCS3 | 2.27E-115 | -0.833880648 | 0.158 | 0.355 | 5.36E-111 |
| NBEAL1 | 1.63E-114 | 0.391163791 | 0.57 | 0.348 | 3.84E-110 |
| VCL | 2.50E-114 | 0.369909743 | 0.691 | 0.48 | 5.90E-110 |
| GJA1 | 6.33E-114 | 0.355373258 | 0.487 | 0.267 | 1.49E-109 |
| NDUFC1 | 1.44E-113 | 0.348596144 | 0.839 | 0.672 | 3.40E-109 |
| GSTP1 | 8.96E-113 | 0.331271097 | 0.968 | 0.919 | 2.12E-108 |
| RAB23 | 5.44E-112 | 0.297391896 | 0.313 | 0.138 | 1.29E-107 |
| MINOS1 | 5.68E-112 | 0.349078375 | 0.777 | 0.568 | 1.34E-107 |
| PLP2 | 1.02E-111 | 0.399085057 | 0.812 | 0.606 | 2.41E-107 |
| GUCY1A1 | 2.33E-111 | -0.769444859 | 0.426 | 0.572 | 5.51E-107 |
| RASL12 | 2.67E-111 | 0.281784002 | 0.378 | 0.182 | 6.31E-107 |
| RPS28 | 7.58E-111 | 0.290869669 | 0.972 | 0.948 | 1.79E-106 |
| MT-CO3 | 1.27E-110 | -0.420889841 | 0.989 | 0.974 | 2.99E-106 |
| PKD2 | 2.88E-110 | 0.384469497 | 0.473 | 0.264 | 6.79E-106 |
| VIM | 1.41E-109 | 0.262418143 | 0.995 | 0.992 | 3.33E-105 |
| SCARA3 | 4.27E-109 | 0.305332461 | 0.421 | 0.214 | 1.01E-104 |
| UBE2H | 5.91E-109 | 0.348000096 | 0.613 | 0.394 | 1.39E-104 |
| RPL26 | 1.04E-108 | 0.290030452 | 0.973 | 0.942 | 2.45E-104 |
| CALD1 | 1.45E-108 | 0.304302634 | 0.982 | 0.971 | 3.42E-104 |
| PAM | 2.63E-108 | 0.349466129 | 0.589 | 0.37 | 6.22E-104 |
| ACTA2 | 5.64E-108 | 0.396442846 | 0.925 | 0.768 | 1.33E-103 |
| GLIPR1 | 1.43E-107 | 0.417194842 | 0.689 | 0.482 | 3.38E-103 |
| SVIP | 2.45E-107 | 0.292640169 | 0.473 | 0.257 | 5.78E-103 |
| RPL27 | 2.05E-106 | 0.319028356 | 0.941 | 0.891 | 4.83E-102 |
| SERPINE1 | 3.51E-106 | 0.68688904 | 0.406 | 0.214 | 8.28E-102 |
| TSPAN2 | 3.52E-106 | 0.252416744 | 0.445 | 0.229 | 8.31E-102 |
| FAM129A | 8.31E-106 | 0.359424254 | 0.681 | 0.458 | 1.96E-101 |
| C1S | 8.36E-106 | -1.048921661 | 0.456 | 0.587 | 1.97E-101 |
| A2M | 1.93E-105 | 0.37427051 | 0.901 | 0.722 | 4.56E-101 |
| RPL15 | 3.37E-105 | 0.288936475 | 0.976 | 0.95 | 7.95E-101 |
| SERPINF1 | 7.83E-105 | -1.430986055 | 0.086 | 0.258 | 1.85E-100 |
| COL21A1 | 9.44E-105 | 0.383130151 | 0.557 | 0.337 | 2.23E-100 |
| SEC61G | 3.36E-104 | 0.327370677 | 0.839 | 0.684 | 7.92E-100 |
| ELOB | 4.83E-104 | 0.294498466 | 0.922 | 0.818 | 1.14E-99 |
| SMOC2 | 1.35E-103 | 0.392505563 | 0.705 | 0.493 | 3.19E-99 |
| DMKN | 1.50E-103 | 0.250693395 | 0.348 | 0.163 | 3.55E-99 |
| LUM | 2.22E-103 | -0.843901978 | 0.205 | 0.413 | 5.25E-99 |
| MAP1B | 2.45E-103 | 0.41407378 | 0.906 | 0.788 | 5.78E-99 |
| ALCAM | 2.75E-103 | 0.359181091 | 0.529 | 0.31 | 6.48E-99 |
| PCOLCE | 1.20E-102 | -0.637907526 | 0.426 | 0.587 | 2.84E-98 |
| ADGRE5 | 2.31E-102 | 0.307074558 | 0.439 | 0.239 | 5.44E-98 |
| IL33 | 2.20E-100 | -0.537397268 | 0.141 | 0.319 | 5.20E-96 |
| BTG2 | 2.40E-99 | -1.149232421 | 0.307 | 0.461 | 5.67E-95 |
| MSRB3 | 3.09E-99 | 0.354247909 | 0.744 | 0.539 | 7.30E-95 |
| RBPMS | 3.58E-99 | 0.328976375 | 0.724 | 0.506 | 8.44E-95 |
| SNRPG | 6.18E-99 | 0.309323373 | 0.705 | 0.497 | 1.46E-94 |
| COX7A2 | 6.93E-99 | 0.309331183 | 0.903 | 0.795 | 1.64E-94 |
| MSN | 8.64E-99 | 0.335508005 | 0.67 | 0.46 | 2.04E-94 |
| RPL37 | 7.13E-98 | 0.282648725 | 0.975 | 0.954 | 1.68E-93 |
| MARCKS | 9.92E-98 | -0.639833925 | 0.292 | 0.475 | 2.34E-93 |
| FRZB | 1.48E-97 | 0.284173501 | 0.852 | 0.654 | 3.50E-93 |
| KIF5B | 1.85E-97 | 0.354876126 | 0.787 | 0.611 | 4.36E-93 |
| KCNMB1 | 3.84E-97 | 0.28221896 | 0.56 | 0.335 | 9.07E-93 |
| ROMO1 | 6.37E-97 | 0.324554101 | 0.694 | 0.491 | 1.50E-92 |
| PXDC1 | 1.57E-96 | 0.364194869 | 0.575 | 0.371 | 3.70E-92 |
| COX20 | 1.60E-95 | 0.318533938 | 0.65 | 0.441 | 3.77E-91 |
| GSN | 1.95E-95 | -1.621135898 | 0.937 | 0.919 | 4.61E-91 |
| ZYX | 2.13E-95 | 0.273394824 | 0.609 | 0.384 | 5.03E-91 |
| NCKAP1 | 5.77E-95 | 0.267794929 | 0.494 | 0.287 | 1.36E-90 |
| FOXN3 | 2.77E-94 | 0.27478714 | 0.431 | 0.236 | 6.54E-90 |
| ADH1B | 3.40E-94 | -1.000395614 | 0.323 | 0.498 | 8.04E-90 |
| IFI16 | 3.91E-94 | -0.553497845 | 0.334 | 0.497 | 9.23E-90 |
| BCAM | 1.87E-93 | 0.256873494 | 0.462 | 0.254 | 4.40E-89 |
| METAP2 | 2.40E-93 | 0.317621551 | 0.64 | 0.432 | 5.65E-89 |
| ARPP19 | 4.44E-93 | 0.282185968 | 0.484 | 0.281 | 1.05E-88 |
| SYNE1 | 7.93E-93 | 0.355542126 | 0.631 | 0.425 | 1.87E-88 |
| CRISPLD1 | 1.43E-92 | 0.279602031 | 0.458 | 0.255 | 3.37E-88 |
| HLA-E | 3.20E-92 | -0.565528505 | 0.675 | 0.729 | 7.56E-88 |
| AQP1 | 5.21E-92 | 0.277643451 | 0.727 | 0.497 | 1.23E-87 |
| IER2 | 5.63E-92 | -0.965883523 | 0.611 | 0.675 | 1.33E-87 |
| PDGFRL | 7.68E-92 | -0.709769699 | 0.115 | 0.278 | 1.81E-87 |
| GADD45B | 4.11E-91 | -1.207008988 | 0.498 | 0.605 | 9.71E-87 |
| LIMCH1 | 7.37E-91 | 0.293962517 | 0.668 | 0.443 | 1.74E-86 |
| PTMS | 3.50E-90 | 0.291000426 | 0.931 | 0.817 | 8.27E-86 |
| SOD3 | 1.52E-89 | 0.326779982 | 0.951 | 0.89 | 3.59E-85 |
| TIMP1 | 7.39E-89 | -0.483291805 | 0.96 | 0.939 | 1.74E-84 |
| COX17 | 9.82E-89 | 0.303808247 | 0.691 | 0.486 | 2.32E-84 |
| CRIP2 | 1.42E-88 | 0.320325061 | 0.887 | 0.723 | 3.35E-84 |
| AKAP12 | 1.44E-88 | -0.729993715 | 0.24 | 0.395 | 3.39E-84 |
| SBDS | 7.02E-88 | 0.330457288 | 0.888 | 0.78 | 1.66E-83 |
| NINJ1 | 7.25E-88 | 0.287145204 | 0.48 | 0.279 | 1.71E-83 |
| RPL23 | 8.96E-88 | -0.41806861 | 0.913 | 0.874 | 2.12E-83 |
| ZEB1 | 1.46E-87 | 0.269611718 | 0.523 | 0.319 | 3.46E-83 |
| DPYSL3 | 1.81E-86 | 0.279120176 | 0.523 | 0.323 | 4.27E-82 |
| MBNL1-AS1 | 2.97E-86 | 0.529017386 | 0.453 | 0.268 | 7.01E-82 |
| DDIT4 | 3.97E-86 | -0.681695971 | 0.103 | 0.259 | 9.36E-82 |
| FGF7 | 5.56E-86 | -0.740948272 | 0.115 | 0.274 | 1.31E-81 |
| SELENOK | 6.66E-86 | 0.298983014 | 0.795 | 0.613 | 1.57E-81 |
| CCNI | 4.43E-85 | 0.301976666 | 0.939 | 0.863 | 1.05E-80 |
| MT-CO2 | 4.62E-85 | -0.313428275 | 0.988 | 0.973 | 1.09E-80 |
| COPS9 | 8.64E-85 | 0.288610125 | 0.783 | 0.614 | 2.04E-80 |
| NDUFB7 | 2.22E-84 | 0.279957568 | 0.865 | 0.718 | 5.25E-80 |
| CCDC107 | 2.60E-84 | 0.309504095 | 0.784 | 0.609 | 6.13E-80 |
| CCDC3 | 3.37E-84 | 0.268705136 | 0.592 | 0.374 | 7.96E-80 |
| BCAP29 | 3.72E-84 | 0.292787133 | 0.75 | 0.554 | 8.78E-80 |
| CAMTA1 | 7.35E-84 | 0.292070037 | 0.761 | 0.577 | 1.74E-79 |
| MYH9 | 1.06E-83 | 0.305433621 | 0.811 | 0.63 | 2.51E-79 |
| SELENOM | 2.02E-83 | 0.277212347 | 0.961 | 0.918 | 4.77E-79 |
| CHMP3 | 3.37E-83 | 0.275993368 | 0.593 | 0.392 | 7.96E-79 |
| PDLIM3 | 1.35E-82 | 0.332700332 | 0.864 | 0.711 | 3.20E-78 |
| MFAP4 | 1.76E-82 | 0.331196199 | 0.815 | 0.674 | 4.14E-78 |
| EMP2 | 4.10E-82 | 0.310353109 | 0.838 | 0.686 | 9.69E-78 |
| AGT | 5.70E-82 | -0.683618202 | 0.129 | 0.285 | 1.35E-77 |
| TIMM8B | 7.46E-82 | 0.250291197 | 0.545 | 0.344 | 1.76E-77 |
| UQCRQ | 9.63E-82 | 0.283121086 | 0.827 | 0.682 | 2.27E-77 |
| RPL31 | 1.27E-81 | -0.4272312 | 0.829 | 0.808 | 3.01E-77 |
| EIF5B | 1.87E-80 | 0.276225196 | 0.758 | 0.56 | 4.42E-76 |
| H3F3B | 2.65E-80 | -0.292805597 | 0.969 | 0.952 | 6.26E-76 |
| OMD | 3.03E-80 | -0.797180193 | 0.353 | 0.489 | 7.16E-76 |
| PLTP | 3.31E-80 | -0.616473529 | 0.127 | 0.276 | 7.82E-76 |
| RPS11 | 8.96E-80 | -0.355299885 | 0.957 | 0.923 | 2.11E-75 |
| APOE | 1.09E-78 | -1.964060029 | 0.108 | 0.256 | 2.57E-74 |
| HSPA1B | 1.37E-78 | -0.884469167 | 0.361 | 0.494 | 3.23E-74 |
| EFHD1 | 3.01E-78 | 0.254467297 | 0.611 | 0.401 | 7.10E-74 |
| SON | 1.54E-77 | 0.251324371 | 0.875 | 0.772 | 3.63E-73 |
| MACF1 | 1.54E-77 | 0.309186846 | 0.655 | 0.47 | 3.64E-73 |
| ARPC1B | 8.89E-77 | 0.313758572 | 0.626 | 0.429 | 2.10E-72 |
| C1orf122 | 3.16E-76 | 0.254569418 | 0.676 | 0.473 | 7.47E-72 |
| TFPI | 1.07E-75 | -0.622044326 | 0.392 | 0.513 | 2.53E-71 |
| BST2 | 2.21E-75 | -0.467628534 | 0.316 | 0.495 | 5.21E-71 |
| UBC | 5.52E-75 | 0.266568239 | 0.97 | 0.945 | 1.30E-70 |
| YWHAH | 5.65E-75 | 0.303550353 | 0.705 | 0.518 | 1.33E-70 |
| FXYD1 | 1.92E-74 | 0.284437439 | 0.866 | 0.751 | 4.52E-70 |
| SELENOW | 2.15E-74 | 0.264669243 | 0.952 | 0.894 | 5.08E-70 |
| HLA-DRB1 | 4.86E-74 | -0.696729102 | 0.113 | 0.263 | 1.15E-69 |
| KLF2 | 3.17E-73 | -0.847492267 | 0.471 | 0.571 | 7.48E-69 |
| PPFIBP1 | 2.04E-72 | 0.253380319 | 0.62 | 0.424 | 4.81E-68 |
| RNF7 | 3.44E-72 | 0.281430212 | 0.793 | 0.622 | 8.13E-68 |
| RAB32 | 4.58E-72 | 0.304908037 | 0.468 | 0.293 | 1.08E-67 |
| ZFP36L1 | 1.04E-71 | -0.661602391 | 0.479 | 0.572 | 2.44E-67 |
| NDUFB8 | 1.52E-71 | 0.270581125 | 0.833 | 0.685 | 3.59E-67 |
| IRF1 | 1.97E-70 | -0.573399311 | 0.125 | 0.273 | 4.65E-66 |
| SERBP1 | 2.29E-70 | 0.264927294 | 0.824 | 0.671 | 5.40E-66 |
| GLS | 5.98E-70 | 0.259421119 | 0.624 | 0.434 | 1.41E-65 |
| FAM177A1 | 7.49E-70 | 0.253291838 | 0.665 | 0.468 | 1.77E-65 |
| PMP22 | 9.83E-70 | -0.687800125 | 0.521 | 0.59 | 2.32E-65 |
| RRAS | 1.09E-69 | 0.272198414 | 0.89 | 0.775 | 2.58E-65 |
| MT1M | 1.25E-69 | -0.703493757 | 0.287 | 0.421 | 2.94E-65 |
| CAST | 2.96E-69 | -0.418332254 | 0.794 | 0.783 | 6.99E-65 |
| SELENOP | 3.14E-69 | -0.704720919 | 0.28 | 0.413 | 7.40E-65 |
| FNBP1 | 3.39E-69 | 0.253867764 | 0.362 | 0.205 | 8.00E-65 |
| ABI3BP | 3.51E-69 | -0.811375911 | 0.177 | 0.313 | 8.28E-65 |
| C4orf3 | 3.53E-69 | 0.268430818 | 0.88 | 0.745 | 8.34E-65 |
| 7-Sep | 3.86E-69 | -0.342794124 | 0.933 | 0.895 | 9.11E-65 |
| TMSB4X | 4.60E-69 | 0.25696124 | 0.981 | 0.967 | 1.09E-64 |
| CCDC80 | 4.88E-69 | -1.245399225 | 0.494 | 0.566 | 1.15E-64 |
| RPL21 | 8.16E-69 | -0.290021949 | 0.975 | 0.957 | 1.93E-64 |
| NR2F2 | 9.49E-69 | -0.528480258 | 0.546 | 0.637 | 2.24E-64 |
| CSRP2 | 3.56E-68 | 0.262682939 | 0.621 | 0.415 | 8.40E-64 |
| CD59 | 4.49E-68 | -0.446796938 | 0.731 | 0.748 | 1.06E-63 |
| MT1E | 5.36E-68 | -0.633585665 | 0.409 | 0.525 | 1.26E-63 |
| NPC2 | 8.22E-68 | -0.384874279 | 0.636 | 0.699 | 1.94E-63 |
| NOP10 | 6.24E-67 | 0.266015144 | 0.715 | 0.533 | 1.47E-62 |
| SNHG8 | 9.17E-67 | 0.300861905 | 0.683 | 0.506 | 2.16E-62 |
| CD74 | 2.83E-65 | -0.775503032 | 0.195 | 0.344 | 6.68E-61 |
| ANKRD37 | 1.16E-64 | 0.256877548 | 0.342 | 0.194 | 2.73E-60 |
| NUCKS1 | 2.87E-64 | -0.366027851 | 0.89 | 0.864 | 6.78E-60 |
| RSL24D1 | 3.10E-64 | 0.258948 | 0.795 | 0.635 | 7.31E-60 |
| MBNL1 | 4.66E-64 | 0.251596924 | 0.778 | 0.631 | 1.10E-59 |
| PKM | 1.87E-63 | 0.282104228 | 0.851 | 0.709 | 4.41E-59 |
| SERPING1 | 3.36E-63 | -0.537331922 | 0.872 | 0.838 | 7.94E-59 |
| NR4A1 | 1.68E-62 | -1.026276722 | 0.257 | 0.374 | 3.96E-58 |
| HSPB1 | 3.54E-62 | 0.269332822 | 0.966 | 0.925 | 8.35E-58 |
| PSME1 | 3.62E-62 | -0.393130246 | 0.649 | 0.694 | 8.56E-58 |
| PPP1R15A | 5.73E-61 | -0.641409147 | 0.492 | 0.562 | 1.35E-56 |
| ADI1 | 5.87E-61 | 0.260598129 | 0.806 | 0.663 | 1.38E-56 |
| GADD45A | 9.96E-61 | 0.325570601 | 0.679 | 0.503 | 2.35E-56 |
| ADIRF | 6.34E-60 | 0.277142567 | 0.974 | 0.913 | 1.50E-55 |
| TSC22D1 | 1.75E-58 | -0.669180362 | 0.608 | 0.645 | 4.12E-54 |
| NFKBIA | 7.73E-58 | -0.582999649 | 0.542 | 0.615 | 1.83E-53 |
| LTBP1 | 1.23E-55 | 0.286728956 | 0.837 | 0.693 | 2.90E-51 |
| RPS16 | 6.57E-54 | -0.28900426 | 0.966 | 0.936 | 1.55E-49 |
| LDHA | 3.64E-53 | 0.290605782 | 0.916 | 0.82 | 8.60E-49 |
| HLA-DRA | 4.89E-52 | -0.479564115 | 0.128 | 0.25 | 1.15E-47 |
| ARHGAP29 | 5.19E-52 | -0.48434729 | 0.316 | 0.419 | 1.23E-47 |
| CCNL1 | 1.32E-51 | -0.489620658 | 0.275 | 0.387 | 3.11E-47 |
| PSMB9 | 7.24E-51 | -0.344047308 | 0.182 | 0.309 | 1.71E-46 |
| CCL2 | 7.29E-51 | -0.846133229 | 0.189 | 0.318 | 1.72E-46 |
| EPHX1 | 2.02E-49 | -0.457339971 | 0.275 | 0.38 | 4.78E-45 |
| RCN3 | 5.01E-49 | -0.368700969 | 0.268 | 0.384 | 1.18E-44 |
| METTL7A | 7.25E-48 | -0.451089957 | 0.206 | 0.315 | 1.71E-43 |
| LY6E | 1.19E-47 | -0.371027808 | 0.381 | 0.497 | 2.80E-43 |
| PDIA3 | 1.82E-46 | -0.367660146 | 0.767 | 0.748 | 4.29E-42 |
| NRGN | 2.04E-46 | 0.264701664 | 0.442 | 0.292 | 4.82E-42 |
| CTSK | 4.36E-40 | -0.452247474 | 0.306 | 0.399 | 1.03E-35 |
| ZFP36L2 | 6.73E-40 | -0.684433563 | 0.62 | 0.632 | 1.59E-35 |
| SSR4 | 4.81E-39 | -0.293568507 | 0.838 | 0.799 | 1.14E-34 |
| NEAT1 | 7.83E-39 | -0.740967768 | 0.784 | 0.78 | 1.85E-34 |
| ANGPT2 | 4.69E-38 | -0.46810157 | 0.193 | 0.289 | 1.11E-33 |
| CYR61 | 1.15E-36 | -0.561662553 | 0.723 | 0.737 | 2.72E-32 |
| NBL1 | 2.08E-36 | -0.404868714 | 0.53 | 0.583 | 4.90E-32 |
| LRRC17 | 5.99E-36 | -0.457849787 | 0.26 | 0.348 | 1.41E-31 |
| PIK3R1 | 1.38E-35 | -0.567730216 | 0.334 | 0.407 | 3.26E-31 |
| LGALS3BP | 3.61E-35 | -0.376522317 | 0.443 | 0.507 | 8.52E-31 |
| MTUS1 | 6.48E-35 | -0.325866626 | 0.181 | 0.278 | 1.53E-30 |
| LAMA4 | 3.63E-34 | -0.344211326 | 0.168 | 0.259 | 8.57E-30 |
| IL32 | 4.94E-34 | -0.345858157 | 0.151 | 0.25 | 1.17E-29 |
| PLSCR1 | 4.99E-34 | -0.336218423 | 0.233 | 0.327 | 1.18E-29 |
| PSAP | 3.24E-33 | -0.372833791 | 0.804 | 0.771 | 7.66E-29 |
| GPX3 | 8.10E-33 | -0.56756909 | 0.329 | 0.4 | 1.91E-28 |
| BTG1 | 1.42E-32 | -0.498155399 | 0.7 | 0.684 | 3.36E-28 |
| RARRES3 | 9.19E-32 | -0.297350971 | 0.234 | 0.332 | 2.17E-27 |
| CXCL12 | 1.72E-29 | -0.331118933 | 0.373 | 0.463 | 4.07E-25 |
| CDKN1C | 1.02E-28 | -0.456784984 | 0.341 | 0.402 | 2.41E-24 |
| OLFML2B | 4.71E-28 | -0.366271978 | 0.191 | 0.267 | 1.11E-23 |
| TENT5A | 6.65E-27 | -0.37871883 | 0.261 | 0.332 | 1.57E-22 |
| CRYAB | 7.46E-27 | 0.252030609 | 0.908 | 0.831 | 1.76E-22 |
| TSC22D3 | 3.18E-26 | -0.46539212 | 0.596 | 0.608 | 7.49E-22 |
| TCF4 | 3.30E-26 | -0.399774538 | 0.538 | 0.561 | 7.80E-22 |
| PDGFRB | 1.18E-25 | -0.406170553 | 0.377 | 0.435 | 2.78E-21 |
| SELENOF | 1.69E-25 | -0.30532069 | 0.476 | 0.512 | 3.99E-21 |
| PPP1R10 | 1.89E-25 | -0.324643109 | 0.261 | 0.335 | 4.45E-21 |
| TSPAN4 | 2.59E-25 | -0.325949359 | 0.333 | 0.395 | 6.12E-21 |
| LEPROT | 2.63E-25 | -0.256856781 | 0.753 | 0.724 | 6.20E-21 |
| IER3 | 2.66E-25 | -0.507426862 | 0.241 | 0.312 | 6.29E-21 |
| NDN | 8.56E-25 | -0.295238692 | 0.365 | 0.431 | 2.02E-20 |
| GUCY1B1 | 8.90E-25 | -0.320655831 | 0.187 | 0.258 | 2.10E-20 |
| NRP1 | 1.66E-24 | -0.392309955 | 0.229 | 0.297 | 3.93E-20 |
| HNRNPC | 1.93E-24 | -0.288695182 | 0.469 | 0.507 | 4.55E-20 |
| PNRC1 | 2.49E-24 | -0.339506755 | 0.897 | 0.833 | 5.88E-20 |
| DEPP1 | 3.13E-24 | -0.56375858 | 0.223 | 0.294 | 7.38E-20 |
| CYBA | 3.26E-24 | -0.310652327 | 0.447 | 0.504 | 7.68E-20 |
| CITED2 | 2.40E-23 | -0.336637135 | 0.266 | 0.334 | 5.67E-19 |
| HSPA1A | 3.90E-22 | -0.787929271 | 0.725 | 0.687 | 9.21E-18 |
| NUCB2 | 1.08E-21 | -0.35316942 | 0.589 | 0.582 | 2.55E-17 |
| NCOA7 | 2.29E-21 | -0.336106477 | 0.246 | 0.31 | 5.41E-17 |
| VCAN | 6.47E-21 | -0.433763873 | 0.732 | 0.722 | 1.53E-16 |
| PLPP3 | 2.00E-20 | -0.370281436 | 0.192 | 0.251 | 4.72E-16 |
| PLPP1 | 2.00E-20 | -0.354572897 | 0.448 | 0.478 | 4.72E-16 |
| SELENBP1 | 2.08E-20 | -0.274949272 | 0.23 | 0.291 | 4.91E-16 |
| KLF9 | 6.69E-20 | -0.387301135 | 0.366 | 0.402 | 1.58E-15 |
| SDF4 | 7.34E-19 | -0.264046572 | 0.436 | 0.462 | 1.73E-14 |
| DNAJB1 | 9.64E-19 | -0.542079339 | 0.326 | 0.368 | 2.28E-14 |
| EFEMP1 | 1.27E-18 | -0.513459241 | 0.773 | 0.709 | 3.01E-14 |
| HNRNPU | 2.70E-18 | -0.397610699 | 0.641 | 0.623 | 6.38E-14 |
| GLUL | 2.97E-18 | -0.414779681 | 0.314 | 0.356 | 7.02E-14 |
| TOB1 | 6.74E-18 | -0.26906239 | 0.265 | 0.32 | 1.59E-13 |
| ZEB2 | 1.05E-17 | -0.30285193 | 0.353 | 0.397 | 2.48E-13 |
| CDKN1A | 1.37E-17 | -0.406611192 | 0.234 | 0.289 | 3.23E-13 |
| ARHGDIB | 6.77E-17 | -0.317618837 | 0.417 | 0.455 | 1.60E-12 |
| H1FX | 7.79E-17 | -0.31631618 | 0.504 | 0.527 | 1.84E-12 |
| TIMP3 | 3.04E-16 | -0.847624994 | 0.543 | 0.518 | 7.17E-12 |
| RTN3 | 4.22E-16 | -0.274690674 | 0.399 | 0.43 | 9.96E-12 |
| FUS | 7.27E-16 | -0.335564687 | 0.611 | 0.595 | 1.72E-11 |
| ADD3 | 3.13E-15 | -0.328512844 | 0.241 | 0.286 | 7.39E-11 |
| MLEC | 8.16E-15 | -0.252095642 | 0.385 | 0.419 | 1.93E-10 |
| FKBP5 | 9.03E-15 | -0.328265229 | 0.206 | 0.253 | 2.13E-10 |
| PGF | 1.03E-14 | -0.300192286 | 0.229 | 0.282 | 2.43E-10 |
| RCN1 | 1.41E-14 | -0.27102689 | 0.361 | 0.388 | 3.32E-10 |
| ATF3 | 2.18E-14 | -0.507837098 | 0.409 | 0.44 | 5.15E-10 |
| PCOLCE2 | 7.57E-14 | -0.713149353 | 0.251 | 0.29 | 1.79E-09 |
| C2orf40 | 9.56E-14 | -0.411868469 | 0.453 | 0.488 | 2.26E-09 |
| JAK1 | 3.94E-13 | -0.264753949 | 0.487 | 0.494 | 9.30E-09 |
| IER5L | 9.75E-13 | -0.288981073 | 0.298 | 0.341 | 2.30E-08 |
| NNMT | 1.01E-12 | -0.269079471 | 0.783 | 0.753 | 2.38E-08 |
| INMT | 1.01E-12 | -0.338471932 | 0.296 | 0.328 | 2.40E-08 |
| PHLDA2 | 1.49E-12 | -0.297069223 | 0.199 | 0.252 | 3.52E-08 |
| DAB2 | 3.47E-12 | -0.27877587 | 0.233 | 0.276 | 8.18E-08 |
| FCGRT | 3.63E-12 | -0.314470483 | 0.504 | 0.498 | 8.56E-08 |
| SLC38A2 | 6.52E-12 | -0.314401211 | 0.524 | 0.529 | 1.54E-07 |
| CALCOCO2 | 1.98E-11 | -0.264234077 | 0.432 | 0.441 | 4.68E-07 |
| CTSC | 2.67E-11 | -0.309009269 | 0.215 | 0.261 | 6.29E-07 |
| NAMPT | 1.43E-10 | -0.286481928 | 0.226 | 0.262 | 3.37E-06 |
| LIMA1 | 4.83E-10 | -0.321372754 | 0.505 | 0.504 | 1.14E-05 |
| SLIT3 | 1.48E-09 | -0.358442302 | 0.283 | 0.306 | 3.48E-05 |
| DNAJC3 | 1.81E-09 | -0.250484193 | 0.477 | 0.476 | 4.27E-05 |
| CYB5A | 2.06E-09 | -0.324689978 | 0.441 | 0.439 | 4.86E-05 |
| EPB41L2 | 2.64E-09 | -0.291714946 | 0.48 | 0.476 | 6.23E-05 |
| OS9 | 3.24E-09 | -0.251429561 | 0.439 | 0.434 | 7.66E-05 |
| UACA | 3.34E-09 | -0.352940162 | 0.51 | 0.507 | 7.88E-05 |
| CTGF | 4.38E-09 | -0.492245812 | 0.825 | 0.764 | 0.000103325 |
| ANGPT1 | 4.81E-09 | -0.336832102 | 0.358 | 0.372 | 0.000113495 |
| ID2 | 7.66E-09 | -0.343715858 | 0.334 | 0.354 | 0.000180825 |
| ASPH | 8.73E-09 | -0.2537086 | 0.492 | 0.487 | 0.00020597 |
| IL6ST | 1.06E-08 | -0.298016445 | 0.548 | 0.522 | 0.000250164 |
| EPAS1 | 3.56E-08 | -0.32367673 | 0.5 | 0.487 | 0.000840207 |
| RND3 | 5.13E-08 | -0.274044707 | 0.275 | 0.302 | 0.001209868 |
| SULF1 | 8.33E-08 | -0.374621274 | 0.472 | 0.467 | 0.00196719 |
| ALDH2 | 2.37E-07 | -0.290109153 | 0.603 | 0.562 | 0.005602645 |
| OGN | 2.87E-07 | -0.371987106 | 0.846 | 0.777 | 0.006767359 |
| GNG11 | 1.07E-06 | -0.279921355 | 0.536 | 0.509 | 0.025237619 |
| MCL1 | 1.26E-06 | -0.294853798 | 0.426 | 0.427 | 0.029739013 |
| CEBPB | 3.00E-06 | -0.268887873 | 0.594 | 0.551 | 0.070712753 |
| CHD9 | 5.53E-06 | -0.252512992 | 0.483 | 0.468 | 0.130581162 |
| RARRES2 | 1.94E-05 | -0.257735772 | 0.565 | 0.563 | 0.457208985 |
| SFRP4 | 8.05E-05 | -1.128098432 | 0.303 | 0.304 | 1 |
| RHOB | 0.000107194 | -0.494161816 | 0.664 | 0.602 | 1 |
| ADAMTS1 | 0.000774255 | -0.358062792 | 0.411 | 0.403 | 1 |
| GPNMB | 0.001623641 | -0.270811557 | 0.522 | 0.432 | 1 |
| FGL2 | 0.01016997 | -0.481390616 | 0.621 | 0.497 | 1 |
| ZBTB20 | 0.020346291 | -0.252367446 | 0.576 | 0.534 | 1 |
| WISP2 | 0.042081815 | -0.382729463 | 0.372 | 0.326 | 1 |
| SERTAD1 | 0.050787946 | -0.250646246 | 0.406 | 0.378 | 1 |
| FBN1 | 0.181884126 | -0.65880249 | 0.399 | 0.36 | 1 |
| MMP2 | 0.308083563 | -0.341921076 | 0.358 | 0.338 | 1 |
| PTGIS | 0.430024693 | -0.307555007 | 0.5 | 0.439 | 1 |
| IGFBP6 | 0.799206197 | -0.985944629 | 0.831 | 0.714 | 1 |

**Supplementary Table 4**. DEGs in T cells within atherosclerotic plaque tissue

| Gene | p_val | avg_log2FC | Percentage of cells expressing genes in Control | Percentage of cells expressing genes in AS | p_val_adj |
| --- | --- | --- | --- | --- | --- |
| LGALS3 | 2.65E-40 | 0.669420887 | 0.378 | 0.27 | 6.25E-36 |
| CSTB | 8.57E-31 | 0.578715634 | 0.492 | 0.425 | 2.02E-26 |
| IGKC | 1.55E-56 | 0.573426036 | 0.59 | 0.418 | 3.67E-52 |
| PLP2 | 4.78E-75 | 0.548041281 | 0.579 | 0.41 | 1.13E-70 |
| S100A10 | 2.44E-60 | 0.485654637 | 0.871 | 0.81 | 5.76E-56 |
| HIST1H4C | 3.15E-20 | 0.48220294 | 0.44 | 0.365 | 7.45E-16 |
| S100A11 | 4.18E-53 | 0.482136793 | 0.757 | 0.669 | 9.87E-49 |
| RGCC | 4.46E-31 | 0.455464589 | 0.755 | 0.705 | 1.05E-26 |
| GZMK | 8.54E-28 | 0.454736477 | 0.642 | 0.557 | 2.02E-23 |
| LGALS1 | 9.61E-29 | 0.453355619 | 0.583 | 0.492 | 2.27E-24 |
| VIM | 1.03E-69 | 0.435984906 | 0.965 | 0.958 | 2.43E-65 |
| HLA-DRB1 | 8.57E-12 | 0.423852531 | 0.486 | 0.441 | 2.02E-07 |
| S100A4 | 4.57E-50 | 0.415275847 | 0.9 | 0.855 | 1.08E-45 |
| HLA-DPA1 | 1.04E-20 | 0.413804929 | 0.5 | 0.431 | 2.44E-16 |
| CLIC1 | 1.31E-51 | 0.404460277 | 0.813 | 0.753 | 3.08E-47 |
| FABP5 | 5.41E-16 | 0.403827679 | 0.273 | 0.209 | 1.28E-11 |
| FTL | 1.58E-41 | 0.401186839 | 0.966 | 0.946 | 3.72E-37 |
| CD74 | 1.04E-24 | 0.399751058 | 0.742 | 0.685 | 2.46E-20 |
| GSTP1 | 7.88E-15 | 0.399609973 | 0.413 | 0.362 | 1.86E-10 |
| EMP3 | 1.27E-39 | 0.392658318 | 0.734 | 0.68 | 2.99E-35 |
| GAPDH | 1.50E-53 | 0.39008802 | 0.947 | 0.938 | 3.54E-49 |
| APOBEC3G | 1.80E-20 | 0.376800662 | 0.475 | 0.403 | 4.26E-16 |
| SH3BGRL3 | 2.10E-59 | 0.368062345 | 0.944 | 0.922 | 4.96E-55 |
| CD81 | 2.52E-27 | 0.367778442 | 0.673 | 0.619 | 5.95E-23 |
| FTH1 | 8.98E-53 | 0.360955582 | 0.98 | 0.971 | 2.12E-48 |
| GZMA | 3.88E-26 | 0.35332742 | 0.727 | 0.663 | 9.16E-22 |
| ISG15 | 1.57E-06 | 0.347213561 | 0.294 | 0.263 | 0.037059244 |
| CKLF | 1.56E-24 | 0.338920361 | 0.522 | 0.443 | 3.67E-20 |
| TPI1 | 1.34E-22 | 0.336661245 | 0.618 | 0.568 | 3.16E-18 |
| ALOX5AP | 2.92E-18 | 0.336294944 | 0.528 | 0.469 | 6.89E-14 |
| S100A6 | 5.16E-44 | 0.335122205 | 0.881 | 0.848 | 1.22E-39 |
| MYL6 | 1.11E-42 | 0.32947835 | 0.887 | 0.87 | 2.61E-38 |
| MYL12A | 1.88E-52 | 0.326893688 | 0.921 | 0.896 | 4.45E-48 |
| ACTB | 1.38E-41 | 0.32385178 | 0.981 | 0.984 | 3.25E-37 |
| CXCR3 | 1.19E-10 | 0.322802997 | 0.282 | 0.235 | 2.81E-06 |
| H1FX | 4.78E-08 | 0.319319477 | 0.4 | 0.368 | 0.001127174 |
| PFN1 | 1.09E-36 | 0.319088727 | 0.927 | 0.911 | 2.58E-32 |
| CTSC | 1.67E-09 | 0.312384066 | 0.446 | 0.408 | 3.95E-05 |
| PSME2 | 1.23E-12 | 0.309781873 | 0.545 | 0.518 | 2.90E-08 |
| H3F3B | 7.52E-53 | 0.308033242 | 0.967 | 0.967 | 1.78E-48 |
| PSMB9 | 1.32E-17 | 0.307881581 | 0.604 | 0.564 | 3.13E-13 |
| PSMA7 | 4.46E-24 | 0.307562419 | 0.698 | 0.66 | 1.05E-19 |
| DNAJC15 | 3.61E-17 | 0.306129208 | 0.262 | 0.196 | 8.52E-13 |
| TAGLN2 | 9.49E-23 | 0.298092903 | 0.722 | 0.682 | 2.24E-18 |
| IGLC2 | 4.34E-17 | 0.297246292 | 0.37 | 0.293 | 1.02E-12 |
| TXN | 1.87E-19 | 0.296199911 | 0.543 | 0.486 | 4.41E-15 |
| REEP5 | 8.91E-12 | 0.29486784 | 0.412 | 0.363 | 2.10E-07 |
| HLA-DPB1 | 6.91E-10 | 0.294739584 | 0.439 | 0.396 | 1.63E-05 |
| TUBA1A | 5.14E-11 | 0.293973297 | 0.544 | 0.51 | 1.21E-06 |
| LY6E | 2.01E-11 | 0.293268489 | 0.522 | 0.491 | 4.73E-07 |
| TRBC2 | 5.80E-17 | 0.292875151 | 0.781 | 0.766 | 1.37E-12 |
| PPP1R14B | 1.04E-10 | 0.287774837 | 0.311 | 0.26 | 2.46E-06 |
| CTSW | 4.23E-09 | 0.287704017 | 0.399 | 0.359 | 9.98E-05 |
| TIMP1 | 9.59E-07 | 0.281762548 | 0.333 | 0.302 | 0.022633957 |
| JPT1 | 2.49E-08 | 0.276757599 | 0.401 | 0.372 | 0.000588823 |
| ANXA1 | 1.11E-15 | 0.274482112 | 0.834 | 0.824 | 2.63E-11 |
| MRPS6 | 1.08E-09 | 0.272001739 | 0.292 | 0.247 | 2.55E-05 |
| CYBA | 1.13E-29 | 0.270680454 | 0.845 | 0.823 | 2.67E-25 |
| FAM96B | 4.26E-11 | 0.269580787 | 0.391 | 0.347 | 1.01E-06 |
| CD99 | 9.69E-18 | 0.269457953 | 0.714 | 0.688 | 2.29E-13 |
| MYL12B | 4.30E-29 | 0.269272075 | 0.831 | 0.806 | 1.02E-24 |
| TSPO | 1.43E-14 | 0.266712472 | 0.523 | 0.48 | 3.37E-10 |
| COX5A | 1.36E-07 | 0.265741956 | 0.396 | 0.372 | 0.003201101 |
| RPS27L | 7.40E-10 | 0.263254737 | 0.46 | 0.423 | 1.75E-05 |
| GNG5 | 9.18E-13 | 0.26245108 | 0.455 | 0.409 | 2.17E-08 |
| ARPC3 | 1.95E-23 | 0.260975028 | 0.769 | 0.742 | 4.59E-19 |
| COX6A1 | 1.20E-14 | 0.260655242 | 0.605 | 0.577 | 2.83E-10 |
| RABAC1 | 1.55E-09 | 0.258675259 | 0.476 | 0.445 | 3.66E-05 |
| PSMB6 | 3.80E-10 | 0.257279763 | 0.375 | 0.338 | 8.98E-06 |
| HLA-B | 6.34E-64 | 0.256830659 | 0.989 | 0.99 | 1.50E-59 |
| CCDC85B | 6.48E-12 | 0.253246992 | 0.522 | 0.493 | 1.53E-07 |
| COX8A | 3.15E-09 | 0.252732128 | 0.523 | 0.504 | 7.43E-05 |
| ATP6V1F | 3.13E-07 | 0.250472333 | 0.361 | 0.335 | 0.00739425 |
| ATP5MC3 | 2.48E-09 | 0.250030927 | 0.491 | 0.469 | 5.86E-05 |
| ARID5B | 4.28E-18 | -0.250190902 | 0.267 | 0.362 | 1.01E-13 |
| CLEC2D | 1.74E-19 | -0.250855495 | 0.314 | 0.427 | 4.11E-15 |
| SRSF5 | 1.72E-20 | -0.252094724 | 0.383 | 0.491 | 4.05E-16 |
| SLA | 3.07E-19 | -0.253054896 | 0.344 | 0.456 | 7.26E-15 |
| RPL34 | 6.18E-45 | -0.25337553 | 0.971 | 0.976 | 1.46E-40 |
| RBM39 | 1.29E-27 | -0.25718171 | 0.508 | 0.641 | 3.04E-23 |
| STK4 | 1.74E-22 | -0.257671317 | 0.578 | 0.689 | 4.12E-18 |
| RPLP2 | 6.05E-56 | -0.259610887 | 0.96 | 0.974 | 1.43E-51 |
| MT-CO3 | 3.79E-44 | -0.260822988 | 0.965 | 0.979 | 8.95E-40 |
| PABPC1 | 3.78E-25 | -0.262466289 | 0.647 | 0.743 | 8.92E-21 |
| DDX17 | 5.12E-22 | -0.266073575 | 0.275 | 0.388 | 1.21E-17 |
| PTGER4 | 8.52E-20 | -0.267576707 | 0.318 | 0.425 | 2.01E-15 |
| NKTR | 3.52E-20 | -0.26947747 | 0.209 | 0.306 | 8.30E-16 |
| IKZF1 | 4.58E-26 | -0.275657329 | 0.258 | 0.379 | 1.08E-21 |
| TNFAIP3 | 3.93E-16 | -0.28411576 | 0.457 | 0.551 | 9.27E-12 |
| MT-ND3 | 1.02E-50 | -0.286266614 | 0.849 | 0.922 | 2.41E-46 |
| RPS3A | 1.88E-29 | -0.286350172 | 0.959 | 0.968 | 4.43E-25 |
| RPS9 | 1.30E-52 | -0.286757795 | 0.907 | 0.944 | 3.07E-48 |
| MT-ATP6 | 3.86E-52 | -0.289228889 | 0.933 | 0.971 | 9.11E-48 |
| MT-ND4 | 5.61E-45 | -0.290666078 | 0.915 | 0.954 | 1.32E-40 |
| RPS18 | 1.24E-64 | -0.294600769 | 0.975 | 0.979 | 2.92E-60 |
| CRYBG1 | 5.10E-23 | -0.295070316 | 0.258 | 0.368 | 1.20E-18 |
| RPL36 | 2.06E-52 | -0.297499697 | 0.87 | 0.923 | 4.87E-48 |
| RPL31 | 2.50E-41 | -0.297931518 | 0.727 | 0.821 | 5.91E-37 |
| PIK3R1 | 6.52E-21 | -0.298936127 | 0.36 | 0.466 | 1.54E-16 |
| CNOT6L | 9.60E-23 | -0.299151717 | 0.295 | 0.405 | 2.27E-18 |
| TOMM7 | 5.29E-37 | -0.30424166 | 0.707 | 0.793 | 1.25E-32 |
| CCNL1 | 9.80E-25 | -0.307422681 | 0.36 | 0.48 | 2.31E-20 |
| ZC3HAV1 | 1.19E-23 | -0.308522708 | 0.222 | 0.328 | 2.82E-19 |
| SRSF3 | 5.60E-27 | -0.310190902 | 0.422 | 0.549 | 1.32E-22 |
| EIF4A2 | 1.01E-32 | -0.320896634 | 0.36 | 0.497 | 2.39E-28 |
| SRSF7 | 2.00E-28 | -0.320955249 | 0.626 | 0.741 | 4.72E-24 |
| DDX5 | 1.22E-51 | -0.322371868 | 0.75 | 0.868 | 2.87E-47 |
| RPL9 | 1.62E-58 | -0.331346613 | 0.928 | 0.951 | 3.83E-54 |
| EIF4B | 5.50E-34 | -0.332382367 | 0.282 | 0.42 | 1.30E-29 |
| RPL27 | 1.77E-55 | -0.341008154 | 0.796 | 0.869 | 4.17E-51 |
| AC058791.1 | 3.22E-30 | -0.349201108 | 0.179 | 0.292 | 7.60E-26 |
| RPS16 | 3.37E-76 | -0.352984006 | 0.922 | 0.952 | 7.96E-72 |
| HSP90AA1 | 2.82E-29 | -0.366905451 | 0.778 | 0.86 | 6.66E-25 |
| RPL3 | 1.94E-92 | -0.369277099 | 0.941 | 0.967 | 4.59E-88 |
| RPL37 | 1.39E-87 | -0.376403377 | 0.929 | 0.955 | 3.28E-83 |
| RPS29 | 7.06E-114 | -0.383982066 | 0.934 | 0.959 | 1.67E-109 |
| TPT1 | 5.50E-98 | -0.384384728 | 0.977 | 0.983 | 1.30E-93 |
| EEF2 | 3.18E-44 | -0.385516586 | 0.47 | 0.612 | 7.52E-40 |
| RPL23 | 2.16E-48 | -0.387406018 | 0.729 | 0.819 | 5.09E-44 |
| RPS27 | 1.61E-130 | -0.403197148 | 0.966 | 0.977 | 3.81E-126 |
| RGS1 | 4.65E-15 | -0.40587793 | 0.392 | 0.472 | 1.10E-10 |
| RPL21 | 6.29E-83 | -0.417137274 | 0.931 | 0.958 | 1.48E-78 |
| HSPA1B | 3.08E-13 | -0.421766837 | 0.211 | 0.281 | 7.26E-09 |
| RPS6 | 3.32E-93 | -0.422706582 | 0.925 | 0.956 | 7.83E-89 |
| IL7R | 2.07E-34 | -0.425587306 | 0.583 | 0.693 | 4.88E-30 |
| RPL7 | 5.49E-55 | -0.428090493 | 0.826 | 0.875 | 1.29E-50 |
| RPS11 | 8.81E-75 | -0.428440208 | 0.801 | 0.881 | 2.08E-70 |
| CXCR4 | 5.30E-65 | -0.428821941 | 0.797 | 0.903 | 1.25E-60 |
| RPL4 | 2.28E-57 | -0.429428632 | 0.658 | 0.776 | 5.38E-53 |
| FKBP5 | 1.18E-40 | -0.429445107 | 0.169 | 0.301 | 2.78E-36 |
| NEAT1 | 1.84E-31 | -0.431903263 | 0.412 | 0.544 | 4.34E-27 |
| TSC22D3 | 1.42E-48 | -0.438288966 | 0.809 | 0.893 | 3.36E-44 |
| RPL37A | 2.52E-94 | -0.458237194 | 0.812 | 0.895 | 5.94E-90 |
| RPL38 | 8.93E-94 | -0.480706458 | 0.769 | 0.874 | 2.11E-89 |
| PIK3IP1 | 5.62E-49 | -0.500149669 | 0.193 | 0.343 | 1.33E-44 |
| RPL13A | 5.06E-124 | -0.528695191 | 0.941 | 0.96 | 1.19E-119 |
| HSPA1A | 2.17E-15 | -0.560446856 | 0.32 | 0.404 | 5.13E-11 |
| RPL27A | 1.38E-121 | -0.561078484 | 0.764 | 0.886 | 3.26E-117 |
| TXNIP | 1.08E-83 | -0.572614871 | 0.701 | 0.837 | 2.54E-79 |
| RPS20 | 1.82E-103 | -0.587877578 | 0.803 | 0.879 | 4.30E-99 |

**Supplementary Table 5**. DEGs in macrophages within atherosclerotic plaque tissue

| Gene | p_val | avg_log2FC | Percentage of cells expressing genes in Control | Percentage of cells expressing genes in AS | p_val_adj |
| --- | --- | --- | --- | --- | --- |
| SPP1 | 4.52E-194 | 1.202838096 | 0.631 | 0.337 | 1.07E-189 |
| FTH1 | 4.88E-170 | 0.588554614 | 1 | 0.998 | 1.15E-165 |
| MT-CO3 | 5.79E-160 | -0.520531354 | 0.973 | 0.969 | 1.37E-155 |
| FABP5 | 5.12E-157 | 1.224746986 | 0.787 | 0.609 | 1.21E-152 |
| GAPDH | 6.31E-154 | 0.683368262 | 0.986 | 0.966 | 1.49E-149 |
| CSTB | 1.24E-150 | 1.086092814 | 0.932 | 0.866 | 2.93E-146 |
| MT-ND3 | 2.84E-148 | -0.386842936 | 0.937 | 0.947 | 6.70E-144 |
| DDX5 | 6.15E-138 | -0.571880676 | 0.856 | 0.9 | 1.45E-133 |
| RPL27A | 3.11E-133 | -0.547858598 | 0.859 | 0.906 | 7.33E-129 |
| MT-ATP6 | 1.44E-131 | -0.456890356 | 0.971 | 0.966 | 3.40E-127 |
| TPI1 | 1.11E-130 | 0.681085102 | 0.915 | 0.865 | 2.62E-126 |
| RPL13A | 3.53E-130 | -0.5849189 | 0.928 | 0.941 | 8.34E-126 |
| TMSB4X | 3.55E-129 | -0.453935895 | 0.984 | 0.993 | 8.38E-125 |
| TPT1 | 7.72E-129 | -0.411838482 | 0.99 | 0.991 | 1.82E-124 |
| TIMP1 | 4.98E-128 | 1.049812862 | 0.859 | 0.752 | 1.18E-123 |
| C15orf48 | 1.08E-125 | 0.786069068 | 0.586 | 0.365 | 2.56E-121 |
| S100A10 | 1.83E-119 | 0.648400932 | 0.95 | 0.895 | 4.31E-115 |
| VIM | 6.87E-116 | 0.569154161 | 0.979 | 0.946 | 1.62E-111 |
| RPS11 | 1.18E-115 | -0.45386569 | 0.913 | 0.935 | 2.79E-111 |
| MT-CO2 | 3.25E-115 | -0.357088422 | 0.975 | 0.968 | 7.67E-111 |
| TSC22D3 | 2.42E-114 | -0.860802813 | 0.453 | 0.646 | 5.72E-110 |
| S100A11 | 1.91E-112 | 0.399345138 | 0.987 | 0.976 | 4.50E-108 |
| RPL37A | 4.08E-111 | -0.423663327 | 0.922 | 0.939 | 9.63E-107 |
| RPL21 | 6.45E-108 | -0.353254358 | 0.966 | 0.966 | 1.52E-103 |
| SH3BGRL3 | 2.50E-103 | 0.536129603 | 0.971 | 0.946 | 5.90E-99 |
| CD52 | 3.05E-103 | 0.620089212 | 0.572 | 0.368 | 7.20E-99 |
| FN1 | 6.98E-103 | 0.973815896 | 0.441 | 0.253 | 1.65E-98 |
| RPS29 | 2.79E-101 | -0.386745922 | 0.918 | 0.943 | 6.59E-97 |
| PHLDA1 | 6.97E-101 | 0.663853385 | 0.511 | 0.315 | 1.64E-96 |
| RPL38 | 2.71E-100 | -0.391074271 | 0.864 | 0.908 | 6.40E-96 |
| RPS27 | 1.45E-99 | -0.410830386 | 0.962 | 0.965 | 3.42E-95 |
| FABP4 | 4.66E-99 | 1.447696283 | 0.281 | 0.121 | 1.10E-94 |
| RGCC | 2.21E-98 | 0.731221662 | 0.701 | 0.526 | 5.22E-94 |
| RPL23 | 5.40E-97 | -0.429081041 | 0.903 | 0.92 | 1.27E-92 |
| MYL6 | 2.03E-96 | 0.358287511 | 0.981 | 0.971 | 4.80E-92 |
| SERF2 | 4.27E-95 | 0.335698168 | 0.986 | 0.97 | 1.01E-90 |
| MT-ND1 | 4.47E-91 | -0.405403332 | 0.959 | 0.957 | 1.06E-86 |
| SLAMF9 | 2.37E-90 | 0.487177571 | 0.259 | 0.111 | 5.60E-86 |
| RPL3 | 1.55E-88 | -0.370985187 | 0.942 | 0.953 | 3.66E-84 |
| FKBP5 | 2.46E-85 | -0.56253701 | 0.244 | 0.421 | 5.81E-81 |
| CLIC1 | 2.48E-84 | 0.332138957 | 0.959 | 0.935 | 5.84E-80 |
| ENO1 | 2.67E-84 | 0.53162752 | 0.863 | 0.798 | 6.31E-80 |
| GSTO1 | 4.54E-84 | 0.460904359 | 0.846 | 0.777 | 1.07E-79 |
| MARCO | 1.27E-83 | 0.649632743 | 0.438 | 0.26 | 2.99E-79 |
| LGALS3 | 5.88E-83 | 0.660962911 | 0.882 | 0.802 | 1.39E-78 |
| FBP1 | 9.57E-80 | 0.73794446 | 0.582 | 0.426 | 2.26E-75 |
| RPS9 | 1.05E-79 | -0.294214567 | 0.97 | 0.97 | 2.48E-75 |
| TNFAIP3 | 2.17E-79 | -0.61643014 | 0.221 | 0.402 | 5.12E-75 |
| CTSD | 4.08E-77 | 0.620243033 | 0.897 | 0.854 | 9.63E-73 |
| APOC1 | 4.46E-77 | 0.81679414 | 0.544 | 0.37 | 1.05E-72 |
| CD68 | 5.69E-77 | 0.416357631 | 0.921 | 0.896 | 1.34E-72 |
| PABPC1 | 6.54E-77 | -0.39886289 | 0.854 | 0.898 | 1.54E-72 |
| RPS20 | 1.04E-76 | -0.473399408 | 0.883 | 0.883 | 2.47E-72 |
| MT-ND4 | 3.11E-76 | -0.275732035 | 0.972 | 0.965 | 7.33E-72 |
| MIF | 9.67E-76 | 0.502850308 | 0.673 | 0.547 | 2.28E-71 |
| RPS16 | 1.90E-74 | -0.322368525 | 0.954 | 0.957 | 4.48E-70 |
| ATP6V1F | 2.82E-74 | 0.40355383 | 0.888 | 0.835 | 6.66E-70 |
| CTSL | 4.72E-74 | 0.74106252 | 0.79 | 0.69 | 1.11E-69 |
| GNG5 | 7.88E-74 | 0.331535112 | 0.921 | 0.878 | 1.86E-69 |
| YBX1 | 1.18E-73 | 0.298409295 | 0.971 | 0.95 | 2.78E-69 |
| RPS19 | 3.94E-73 | -0.32628348 | 0.968 | 0.969 | 9.29E-69 |
| DUSP1 | 7.27E-73 | -0.529680279 | 0.877 | 0.911 | 1.71E-68 |
| PGK1 | 1.53E-72 | 0.539665972 | 0.875 | 0.827 | 3.62E-68 |
| GPNMB | 3.60E-71 | 0.441125962 | 0.578 | 0.399 | 8.50E-67 |
| RPL27 | 9.55E-70 | -0.312848062 | 0.908 | 0.925 | 2.25E-65 |
| HLA-DQB1 | 1.05E-69 | -0.487631889 | 0.695 | 0.824 | 2.48E-65 |
| RPS6 | 1.35E-69 | -0.309277753 | 0.956 | 0.957 | 3.19E-65 |
| FTL | 4.95E-67 | 0.441775997 | 1 | 0.998 | 1.17E-62 |
| SMAP2 | 1.58E-66 | -0.480330679 | 0.579 | 0.708 | 3.74E-62 |
| RHOB | 3.62E-66 | -0.596430165 | 0.457 | 0.603 | 8.55E-62 |
| RPL9 | 7.09E-66 | -0.260517976 | 0.96 | 0.961 | 1.67E-61 |
| ZFP36L2 | 1.94E-65 | -0.491654762 | 0.668 | 0.77 | 4.58E-61 |
| LSP1 | 2.47E-65 | 0.374283321 | 0.724 | 0.588 | 5.84E-61 |
| PLIN2 | 4.27E-65 | 0.556679633 | 0.768 | 0.656 | 1.01E-60 |
| CYSTM1 | 3.83E-64 | 0.387500548 | 0.525 | 0.381 | 9.04E-60 |
| RPL23A | 8.52E-64 | -0.299297397 | 0.934 | 0.942 | 2.01E-59 |
| IER5 | 9.13E-64 | -0.506881669 | 0.369 | 0.523 | 2.15E-59 |
| PTPRC | 4.08E-63 | -0.390058787 | 0.836 | 0.885 | 9.62E-59 |
| HSP90AA1 | 4.40E-63 | -0.599715937 | 0.925 | 0.934 | 1.04E-58 |
| IL1RN | 1.22E-62 | 0.715094784 | 0.54 | 0.389 | 2.88E-58 |
| CYP27A1 | 4.99E-62 | 0.405467248 | 0.31 | 0.175 | 1.18E-57 |
| SMIM25 | 6.44E-62 | 0.413417512 | 0.444 | 0.293 | 1.52E-57 |
| PLP2 | 9.79E-62 | 0.335666981 | 0.55 | 0.391 | 2.31E-57 |
| CD36 | 6.75E-61 | 0.869224706 | 0.561 | 0.437 | 1.59E-56 |
| EIF4A2 | 9.12E-61 | -0.39489273 | 0.385 | 0.539 | 2.15E-56 |
| RPL4 | 1.17E-60 | -0.349498401 | 0.781 | 0.843 | 2.76E-56 |
| HLA-DQA1 | 1.47E-60 | -0.468818202 | 0.614 | 0.758 | 3.46E-56 |
| WSB1 | 2.23E-59 | -0.385926593 | 0.535 | 0.67 | 5.25E-55 |
| MS4A6A | 6.09E-58 | -0.385557363 | 0.643 | 0.793 | 1.44E-53 |
| NINJ1 | 6.65E-58 | 0.340229341 | 0.784 | 0.688 | 1.57E-53 |
| PPARG | 1.49E-57 | 0.294944976 | 0.27 | 0.146 | 3.51E-53 |
| PFN1 | 1.52E-57 | 0.256898034 | 0.967 | 0.956 | 3.58E-53 |
| RGS1 | 3.00E-57 | -0.747059973 | 0.456 | 0.567 | 7.08E-53 |
| TUBA1C | 3.83E-57 | 0.380540816 | 0.695 | 0.577 | 9.04E-53 |
| HLA-DPB1 | 2.95E-56 | -0.400211904 | 0.823 | 0.894 | 6.97E-52 |
| FGL2 | 4.15E-56 | -0.407458093 | 0.529 | 0.687 | 9.79E-52 |
| CXCR4 | 5.75E-56 | -0.658046328 | 0.621 | 0.701 | 1.36E-51 |
| SAP30 | 1.11E-55 | -0.478281358 | 0.312 | 0.457 | 2.62E-51 |
| NFKBIZ | 2.10E-55 | -0.421713789 | 0.247 | 0.399 | 4.97E-51 |
| HLA-DRA | 3.16E-55 | -0.364975492 | 0.917 | 0.947 | 7.45E-51 |
| IFI6 | 3.33E-55 | 0.425264381 | 0.551 | 0.423 | 7.85E-51 |
| MT-CYB | 4.22E-55 | -0.286965798 | 0.972 | 0.966 | 9.96E-51 |
| RPL37 | 1.37E-54 | -0.254955928 | 0.974 | 0.968 | 3.23E-50 |
| HLA-DMB | 1.54E-54 | -0.362935378 | 0.578 | 0.714 | 3.63E-50 |
| EEF2 | 1.75E-54 | -0.327148665 | 0.71 | 0.782 | 4.12E-50 |
| TOMM7 | 2.17E-54 | -0.292516127 | 0.765 | 0.831 | 5.12E-50 |
| CYBB | 1.20E-53 | -0.396723776 | 0.656 | 0.765 | 2.84E-49 |
| HLA-C | 5.12E-53 | -0.304042234 | 0.939 | 0.947 | 1.21E-48 |
| MGST3 | 5.48E-53 | 0.392841869 | 0.814 | 0.764 | 1.29E-48 |
| P2RY13 | 1.06E-52 | -0.396969798 | 0.228 | 0.38 | 2.50E-48 |
| PPDPF | 1.37E-52 | 0.330720586 | 0.672 | 0.569 | 3.23E-48 |
| EGR1 | 1.93E-52 | -0.56327104 | 0.147 | 0.279 | 4.56E-48 |
| SOCS3 | 7.70E-52 | -0.542137008 | 0.535 | 0.649 | 1.82E-47 |
| GCHFR | 8.42E-52 | 0.416597572 | 0.331 | 0.206 | 1.99E-47 |
| LGALS1 | 3.86E-51 | 0.410793896 | 0.908 | 0.861 | 9.10E-47 |
| SDC2 | 1.16E-50 | 0.434932351 | 0.37 | 0.246 | 2.75E-46 |
| RNF144B | 1.18E-50 | -0.32851936 | 0.17 | 0.306 | 2.78E-46 |
| RBM39 | 5.25E-50 | -0.339992931 | 0.653 | 0.749 | 1.24E-45 |
| APOE | 6.02E-50 | 0.728537157 | 0.576 | 0.435 | 1.42E-45 |
| PGAM1 | 9.32E-50 | 0.407734898 | 0.705 | 0.622 | 2.20E-45 |
| ATP5MC3 | 1.57E-49 | 0.2861883 | 0.837 | 0.789 | 3.71E-45 |
| CD99 | 4.44E-49 | 0.318628665 | 0.823 | 0.767 | 1.05E-44 |
| HERPUD1 | 6.05E-49 | -0.433717993 | 0.744 | 0.814 | 1.43E-44 |
| RALA | 1.83E-48 | 0.35869002 | 0.61 | 0.492 | 4.32E-44 |
| PPIB | 7.22E-48 | 0.322262064 | 0.883 | 0.851 | 1.70E-43 |
| RGS2 | 7.49E-48 | -0.533960756 | 0.682 | 0.762 | 1.77E-43 |
| NCOA4 | 2.28E-47 | -0.326768706 | 0.407 | 0.546 | 5.38E-43 |
| FXYD5 | 2.47E-47 | 0.287064319 | 0.888 | 0.846 | 5.83E-43 |
| EMP3 | 3.54E-47 | 0.318435989 | 0.87 | 0.818 | 8.35E-43 |
| C1QA | 5.55E-47 | -0.451468255 | 0.474 | 0.62 | 1.31E-42 |
| CD63 | 1.03E-46 | 0.29500647 | 0.969 | 0.945 | 2.43E-42 |
| C4orf3 | 1.63E-46 | 0.35696929 | 0.801 | 0.741 | 3.84E-42 |
| PPP1R14B | 2.39E-46 | 0.348734823 | 0.701 | 0.618 | 5.65E-42 |
| IRF8 | 9.96E-46 | -0.351416761 | 0.323 | 0.457 | 2.35E-41 |
| GLIPR2 | 1.44E-45 | 0.373802432 | 0.565 | 0.449 | 3.40E-41 |
| HSPA1B | 1.47E-45 | -0.698916823 | 0.34 | 0.472 | 3.48E-41 |
| FILIP1L | 4.24E-45 | -0.534720677 | 0.237 | 0.362 | 1.00E-40 |
| FCGR2A | 2.88E-44 | -0.339613621 | 0.698 | 0.777 | 6.81E-40 |
| VSIR | 3.11E-44 | -0.370274922 | 0.442 | 0.574 | 7.34E-40 |
| AKAP13 | 3.76E-44 | -0.360774348 | 0.592 | 0.699 | 8.87E-40 |
| UBC | 6.51E-44 | -0.266258974 | 0.958 | 0.959 | 1.54E-39 |
| SARAF | 7.11E-44 | -0.2854517 | 0.776 | 0.832 | 1.68E-39 |
| PKM | 1.17E-43 | 0.370858715 | 0.81 | 0.764 | 2.76E-39 |
| NLRP3 | 1.46E-43 | -0.349682978 | 0.198 | 0.327 | 3.44E-39 |
| RRP7A | 1.65E-43 | 0.268923097 | 0.451 | 0.329 | 3.90E-39 |
| GAS6 | 1.94E-43 | -0.347138409 | 0.315 | 0.457 | 4.58E-39 |
| CCL2 | 2.18E-43 | 0.661439544 | 0.559 | 0.432 | 5.14E-39 |
| GUK1 | 2.67E-43 | 0.275664299 | 0.845 | 0.791 | 6.31E-39 |
| VCAN | 8.36E-43 | 0.490311355 | 0.548 | 0.408 | 1.97E-38 |
| TNFAIP2 | 9.49E-43 | -0.36033388 | 0.311 | 0.45 | 2.24E-38 |
| LDHA | 1.66E-42 | 0.394973999 | 0.855 | 0.809 | 3.91E-38 |
| IRS2 | 1.71E-42 | -0.352395602 | 0.179 | 0.304 | 4.03E-38 |
| ERO1A | 1.93E-42 | 0.381584501 | 0.418 | 0.307 | 4.55E-38 |
| SELENOP | 2.68E-42 | -0.693908137 | 0.319 | 0.449 | 6.33E-38 |
| FUOM | 2.99E-42 | 0.297733976 | 0.544 | 0.43 | 7.07E-38 |
| EIF4B | 1.32E-41 | -0.28285511 | 0.447 | 0.57 | 3.11E-37 |
| EFHD2 | 1.65E-41 | 0.26664813 | 0.847 | 0.79 | 3.90E-37 |
| HNRNPA2B1 | 1.93E-41 | -0.264202137 | 0.884 | 0.901 | 4.55E-37 |
| JAML | 2.16E-41 | -0.406389517 | 0.304 | 0.435 | 5.09E-37 |
| MMP19 | 2.45E-41 | 0.374560838 | 0.3 | 0.19 | 5.79E-37 |
| ZFAND5 | 3.28E-41 | -0.454504893 | 0.6 | 0.694 | 7.75E-37 |
| AP2S1 | 6.27E-41 | 0.268663562 | 0.844 | 0.805 | 1.48E-36 |
| ZC3HAV1 | 8.96E-41 | -0.346142527 | 0.185 | 0.303 | 2.11E-36 |
| CXXC5 | 1.67E-40 | 0.267681595 | 0.303 | 0.196 | 3.94E-36 |
| RPL7 | 1.69E-40 | -0.285740644 | 0.922 | 0.925 | 3.98E-36 |
| DNAJA1 | 2.05E-40 | -0.354047277 | 0.739 | 0.8 | 4.84E-36 |
| F13A1 | 3.92E-40 | -0.559190231 | 0.402 | 0.532 | 9.26E-36 |
| CCNL1 | 6.67E-40 | -0.335884702 | 0.566 | 0.671 | 1.58E-35 |
| PELI1 | 1.08E-39 | -0.315451526 | 0.18 | 0.299 | 2.55E-35 |
| MGLL | 1.53E-39 | 0.433618826 | 0.382 | 0.273 | 3.60E-35 |
| PPP1R15A | 1.54E-39 | -0.386517544 | 0.738 | 0.796 | 3.64E-35 |
| NCF1 | 2.17E-39 | -0.493585888 | 0.333 | 0.46 | 5.12E-35 |
| RPS4Y1 | 3.22E-39 | 0.278874195 | 0.773 | 0.661 | 7.60E-35 |
| HSPA1A | 6.99E-39 | -0.688491914 | 0.605 | 0.705 | 1.65E-34 |
| MRC1 | 7.06E-39 | -0.524486297 | 0.399 | 0.513 | 1.67E-34 |
| RPS21 | 1.21E-38 | -0.265956498 | 0.893 | 0.915 | 2.85E-34 |
| SAT1 | 8.78E-38 | -0.345603397 | 0.983 | 0.983 | 2.07E-33 |
| EREG | 9.62E-38 | 0.520660077 | 0.319 | 0.208 | 2.27E-33 |
| CD82 | 9.73E-38 | 0.262300348 | 0.26 | 0.16 | 2.30E-33 |
| H2AFY | 1.45E-37 | 0.34318196 | 0.831 | 0.791 | 3.41E-33 |
| STAB1 | 1.58E-37 | -0.509075462 | 0.287 | 0.412 | 3.72E-33 |
| PMAIP1 | 2.44E-37 | -0.381342447 | 0.192 | 0.307 | 5.76E-33 |
| DAD1 | 2.65E-37 | 0.275897729 | 0.797 | 0.751 | 6.25E-33 |
| KCTD12 | 2.69E-37 | -0.317122252 | 0.385 | 0.517 | 6.36E-33 |
| STK17B | 3.02E-37 | -0.346796273 | 0.544 | 0.654 | 7.12E-33 |
| CD302 | 4.08E-37 | -0.28335303 | 0.382 | 0.515 | 9.62E-33 |
| DPYSL2 | 5.87E-37 | -0.261590059 | 0.4 | 0.532 | 1.39E-32 |
| CCL4 | 7.12E-37 | -0.563263908 | 0.43 | 0.549 | 1.68E-32 |
| PPT1 | 8.92E-37 | -0.25459178 | 0.552 | 0.669 | 2.11E-32 |
| VKORC1 | 9.10E-37 | 0.289834381 | 0.589 | 0.493 | 2.15E-32 |
| SRSF5 | 1.10E-36 | -0.299935967 | 0.612 | 0.708 | 2.61E-32 |
| C1QB | 1.35E-36 | -0.376306776 | 0.454 | 0.586 | 3.18E-32 |
| SERPINB9 | 1.81E-36 | -0.277324318 | 0.277 | 0.405 | 4.27E-32 |
| MMP24OS | 2.40E-36 | 0.310943631 | 0.546 | 0.452 | 5.67E-32 |
| NASP | 3.07E-36 | -0.257145726 | 0.367 | 0.491 | 7.24E-32 |
| P4HA1 | 6.54E-36 | 0.375085979 | 0.464 | 0.359 | 1.54E-31 |
| RAB13 | 6.56E-36 | 0.302545494 | 0.532 | 0.433 | 1.55E-31 |
| CALM3 | 7.11E-36 | 0.323152922 | 0.671 | 0.598 | 1.68E-31 |
| PFKFB3 | 8.21E-36 | -0.294701058 | 0.239 | 0.358 | 1.94E-31 |
| NOP53 | 9.85E-36 | -0.259161097 | 0.524 | 0.628 | 2.33E-31 |
| TRIB1 | 1.06E-35 | -0.27523393 | 0.17 | 0.282 | 2.49E-31 |
| ETFB | 1.42E-35 | 0.286498612 | 0.595 | 0.504 | 3.34E-31 |
| HSPH1 | 2.10E-35 | -0.369568502 | 0.295 | 0.412 | 4.96E-31 |
| FYB1 | 2.49E-35 | -0.337632921 | 0.672 | 0.753 | 5.87E-31 |
| AOAH | 4.98E-35 | -0.259964579 | 0.312 | 0.441 | 1.17E-30 |
| ITPR2 | 5.12E-35 | -0.280373809 | 0.304 | 0.426 | 1.21E-30 |
| LPAR6 | 1.12E-34 | -0.324299368 | 0.313 | 0.432 | 2.64E-30 |
| SLC1A3 | 1.19E-34 | -0.301510905 | 0.168 | 0.276 | 2.81E-30 |
| BLVRB | 2.45E-34 | 0.324676398 | 0.831 | 0.797 | 5.79E-30 |
| SRSF3 | 3.04E-34 | -0.287532474 | 0.639 | 0.722 | 7.17E-30 |
| FOLR2 | 3.58E-34 | -0.376951242 | 0.34 | 0.47 | 8.44E-30 |
| BZW1 | 7.94E-34 | 0.329548292 | 0.758 | 0.704 | 1.87E-29 |
| GPBP1 | 9.74E-34 | -0.259360645 | 0.384 | 0.504 | 2.30E-29 |
| MIS18BP1 | 1.81E-33 | -0.312894673 | 0.389 | 0.498 | 4.27E-29 |
| MAT2A | 3.21E-33 | -0.268451684 | 0.326 | 0.444 | 7.58E-29 |
| KLF9 | 3.46E-33 | -0.277286775 | 0.168 | 0.274 | 8.17E-29 |
| PDXK | 4.43E-33 | 0.329182039 | 0.625 | 0.551 | 1.05E-28 |
| IRAK3 | 4.68E-33 | -0.285861509 | 0.205 | 0.314 | 1.10E-28 |
| 1-Mar | 7.40E-33 | -0.276718292 | 0.355 | 0.474 | 1.75E-28 |
| EMB | 7.41E-33 | -0.278752478 | 0.282 | 0.399 | 1.75E-28 |
| ACP5 | 7.78E-33 | 0.364631095 | 0.468 | 0.358 | 1.84E-28 |
| ATP1B3 | 9.47E-33 | -0.595998468 | 0.634 | 0.699 | 2.23E-28 |
| UTRN | 9.99E-33 | -0.254372988 | 0.222 | 0.338 | 2.36E-28 |
| C1QC | 1.62E-32 | -0.282669215 | 0.466 | 0.6 | 3.83E-28 |
| PRDX4 | 1.93E-32 | 0.250723369 | 0.498 | 0.401 | 4.55E-28 |
| HNRNPU | 2.85E-32 | -0.313589923 | 0.765 | 0.821 | 6.73E-28 |
| S100A6 | 3.26E-32 | 0.257714948 | 0.942 | 0.905 | 7.69E-28 |
| BRD2 | 3.65E-32 | -0.289657737 | 0.414 | 0.536 | 8.62E-28 |
| KLF4 | 8.42E-32 | -0.343486866 | 0.608 | 0.696 | 1.99E-27 |
| HNRNPA1 | 1.65E-31 | -0.266347913 | 0.76 | 0.807 | 3.89E-27 |
| FOS | 2.27E-31 | -0.38600101 | 0.92 | 0.928 | 5.36E-27 |
| ALDH2 | 2.97E-31 | -0.262334149 | 0.476 | 0.587 | 7.01E-27 |
| DNAJB1 | 3.05E-31 | -0.385703292 | 0.421 | 0.532 | 7.19E-27 |
| FOSB | 3.54E-31 | -0.406666602 | 0.663 | 0.73 | 8.36E-27 |
| TREM1 | 5.02E-31 | 0.318393866 | 0.457 | 0.357 | 1.19E-26 |
| RNF7 | 5.56E-31 | 0.255349033 | 0.728 | 0.664 | 1.31E-26 |
| RSRP1 | 6.53E-31 | -0.295044639 | 0.498 | 0.608 | 1.54E-26 |
| NAMPT | 9.24E-31 | -0.429620749 | 0.84 | 0.864 | 2.18E-26 |
| H2AFJ | 1.58E-30 | 0.279280583 | 0.632 | 0.551 | 3.73E-26 |
| CTSS | 2.91E-30 | -0.35985365 | 0.921 | 0.934 | 6.86E-26 |
| FAM107B | 3.22E-30 | 0.257735886 | 0.596 | 0.497 | 7.60E-26 |
| DDX3X | 3.28E-30 | -0.268253379 | 0.651 | 0.734 | 7.74E-26 |
| CELF2 | 6.61E-30 | -0.275492868 | 0.384 | 0.492 | 1.56E-25 |
| SEC61G | 1.01E-29 | 0.287542965 | 0.736 | 0.68 | 2.39E-25 |
| GPR34 | 2.85E-29 | -0.302375111 | 0.289 | 0.403 | 6.73E-25 |
| MEF2A | 3.43E-29 | -0.280271649 | 0.393 | 0.508 | 8.08E-25 |
| C3AR1 | 4.33E-29 | -0.31667282 | 0.546 | 0.633 | 1.02E-24 |
| IL10RA | 4.45E-29 | -0.250004994 | 0.39 | 0.501 | 1.05E-24 |
| TET2 | 4.60E-29 | -0.262446073 | 0.29 | 0.405 | 1.09E-24 |
| BIRC3 | 5.38E-29 | -0.409543554 | 0.208 | 0.31 | 1.27E-24 |
| FAM162A | 1.59E-28 | 0.341129824 | 0.5 | 0.421 | 3.75E-24 |
| VMP1 | 1.83E-28 | -0.358890496 | 0.587 | 0.672 | 4.31E-24 |
| CD55 | 2.67E-28 | -0.459608807 | 0.467 | 0.562 | 6.30E-24 |
| CFLAR | 4.89E-28 | -0.273511698 | 0.479 | 0.577 | 1.15E-23 |
| POU2F2 | 6.28E-28 | -0.264374488 | 0.288 | 0.398 | 1.48E-23 |
| GRASP | 1.49E-27 | -0.309775449 | 0.236 | 0.338 | 3.51E-23 |
| LILRB4 | 2.05E-27 | 0.252897379 | 0.647 | 0.564 | 4.84E-23 |
| ZFAS1 | 2.10E-27 | -0.28808534 | 0.634 | 0.712 | 4.97E-23 |
| TAGAP | 3.03E-27 | -0.306892899 | 0.318 | 0.422 | 7.16E-23 |
| FKBP2 | 3.64E-27 | 0.264054409 | 0.733 | 0.671 | 8.60E-23 |
| CLEC7A | 3.91E-27 | -0.26239084 | 0.611 | 0.702 | 9.23E-23 |
| NABP1 | 5.53E-27 | -0.280936381 | 0.436 | 0.542 | 1.30E-22 |
| AMD1 | 2.03E-26 | -0.251157207 | 0.492 | 0.582 | 4.78E-22 |
| PLAUR | 2.51E-26 | 0.328762108 | 0.874 | 0.823 | 5.92E-22 |
| IFITM2 | 3.05E-26 | -0.57180343 | 0.674 | 0.756 | 7.19E-22 |
| CTSB | 3.20E-26 | 0.295679255 | 0.929 | 0.91 | 7.55E-22 |
| ANPEP | 5.68E-26 | 0.283208966 | 0.348 | 0.257 | 1.34E-21 |
| NUFIP2 | 6.01E-26 | -0.262903581 | 0.412 | 0.51 | 1.42E-21 |
| MRPS6 | 6.03E-26 | 0.272965033 | 0.447 | 0.366 | 1.42E-21 |
| SLCO2B1 | 6.38E-26 | -0.257580452 | 0.285 | 0.388 | 1.51E-21 |
| MPP1 | 1.61E-25 | 0.266899746 | 0.534 | 0.456 | 3.80E-21 |
| LY6E | 2.82E-25 | 0.275833426 | 0.531 | 0.445 | 6.65E-21 |
| CCL4L2 | 3.39E-25 | -0.32373848 | 0.353 | 0.456 | 8.01E-21 |
| JMJD1C | 3.87E-25 | -0.289618221 | 0.58 | 0.663 | 9.13E-21 |
| FLNA | 4.65E-25 | 0.273267362 | 0.49 | 0.402 | 1.10E-20 |
| MCL1 | 4.82E-25 | -0.254741812 | 0.813 | 0.847 | 1.14E-20 |
| BTG1 | 6.75E-25 | 0.325849453 | 0.937 | 0.912 | 1.59E-20 |
| KDELR2 | 1.03E-24 | 0.274563105 | 0.639 | 0.584 | 2.44E-20 |
| IRF1 | 2.64E-24 | -0.386896026 | 0.468 | 0.56 | 6.23E-20 |
| PLA2G7 | 3.27E-24 | 0.303131349 | 0.313 | 0.23 | 7.72E-20 |
| NAP1L1 | 4.16E-24 | -0.276814875 | 0.636 | 0.703 | 9.82E-20 |
| JUNB | 7.97E-24 | -0.251496279 | 0.913 | 0.924 | 1.88E-19 |
| MEF2C | 8.83E-24 | -0.269877491 | 0.527 | 0.626 | 2.08E-19 |
| RPL36A | 3.13E-23 | -0.325094729 | 0.526 | 0.618 | 7.38E-19 |
| SRSF7 | 6.48E-23 | -0.278984746 | 0.628 | 0.701 | 1.53E-18 |
| TCF4 | 9.26E-23 | -0.264886866 | 0.238 | 0.328 | 2.19E-18 |
| MAF | 1.01E-22 | -0.368673225 | 0.338 | 0.428 | 2.38E-18 |
| AC020916.1 | 3.29E-22 | -0.300378703 | 0.24 | 0.327 | 7.77E-18 |
| NEAT1 | 8.54E-22 | -0.331716183 | 0.951 | 0.948 | 2.01E-17 |
| MNDA | 1.02E-21 | -0.451683541 | 0.737 | 0.786 | 2.40E-17 |
| INTS6 | 1.38E-21 | -0.27486062 | 0.248 | 0.337 | 3.25E-17 |
| IER2 | 1.56E-21 | -0.454107985 | 0.735 | 0.775 | 3.67E-17 |
| BCAP31 | 2.05E-21 | 0.256878862 | 0.686 | 0.639 | 4.84E-17 |
| CLEC10A | 2.66E-21 | -0.399031448 | 0.204 | 0.286 | 6.28E-17 |
| CPM | 4.34E-21 | -0.250615334 | 0.294 | 0.387 | 1.02E-16 |
| MT1X | 1.40E-20 | 0.666925465 | 0.325 | 0.252 | 3.30E-16 |
| DUSP6 | 2.65E-20 | -0.343351229 | 0.347 | 0.435 | 6.26E-16 |
| LTC4S | 1.66E-18 | -0.288375901 | 0.305 | 0.388 | 3.91E-14 |
| CCL3L1 | 2.42E-18 | -0.321763513 | 0.393 | 0.476 | 5.72E-14 |
| LST1 | 4.75E-18 | -0.283533672 | 0.748 | 0.788 | 1.12E-13 |
| KLF6 | 4.86E-18 | -0.284319855 | 0.806 | 0.856 | 1.15E-13 |
| ACSL1 | 7.76E-18 | -0.273162569 | 0.292 | 0.375 | 1.83E-13 |
| SOD2 | 1.02E-17 | -0.534455131 | 0.566 | 0.599 | 2.40E-13 |
| SAMSN1 | 1.10E-17 | -0.462934515 | 0.463 | 0.53 | 2.59E-13 |
| HMGB2 | 3.95E-17 | -0.302345359 | 0.386 | 0.463 | 9.33E-13 |
| SOX4 | 4.28E-17 | -0.288103375 | 0.211 | 0.287 | 1.01E-12 |
| NR4A2 | 7.06E-17 | -0.272278809 | 0.673 | 0.723 | 1.67E-12 |
| OTUD1 | 8.02E-17 | -0.277301211 | 0.44 | 0.512 | 1.89E-12 |
| SMCHD1 | 8.77E-17 | -0.336030311 | 0.333 | 0.414 | 2.07E-12 |
| KLF2 | 9.40E-17 | -0.29756003 | 0.514 | 0.589 | 2.22E-12 |
| PLBD1 | 3.23E-16 | -0.273265478 | 0.446 | 0.517 | 7.63E-12 |
| GCA | 3.14E-15 | -0.264286835 | 0.502 | 0.572 | 7.42E-11 |
| ABHD5 | 6.06E-15 | -0.270313664 | 0.265 | 0.338 | 1.43E-10 |
| ID2 | 1.08E-14 | -0.267280457 | 0.432 | 0.501 | 2.54E-10 |
| NR4A3 | 7.47E-14 | -0.257403309 | 0.346 | 0.417 | 1.76E-09 |
| PLAU | 9.40E-12 | -0.281647636 | 0.256 | 0.319 | 2.22E-07 |
| VSIG4 | 9.42E-12 | -0.253848436 | 0.526 | 0.571 | 2.22E-07 |
| AC020656.1 | 9.54E-12 | -0.379177534 | 0.38 | 0.442 | 2.25E-07 |
| TXNIP | 1.90E-11 | -0.329265191 | 0.739 | 0.765 | 4.48E-07 |
| IL1B | 6.02E-11 | -0.4078567 | 0.427 | 0.488 | 1.42E-06 |
| CFD | 8.35E-11 | -0.274474712 | 0.721 | 0.751 | 1.97E-06 |
| PLTP | 3.62E-09 | -0.457163882 | 0.514 | 0.539 | 8.55E-05 |
| CCL3 | 1.25E-08 | -0.358344867 | 0.66 | 0.675 | 0.000295212 |
| CSF3R | 1.76E-08 | -0.252547916 | 0.249 | 0.302 | 0.000415224 |
| MS4A4A | 3.92E-08 | -0.262917907 | 0.562 | 0.6 | 0.000924426 |
| TFRC | 4.53E-08 | -0.298078427 | 0.351 | 0.395 | 0.001068422 |
| MTRNR2L8 | 1.16E-07 | 0.396121495 | 0.21 | 0.269 | 0.002744535 |
| S100A9 | 1.51E-06 | -1.100177599 | 0.588 | 0.588 | 0.035714297 |
| SELENOK | 5.54E-06 | 0.281243821 | 0.744 | 0.734 | 0.130799654 |
| MTRNR2L12 | 5.32E-05 | 0.983911334 | 0.632 | 0.686 | 1 |
| MT2A | 0.015474598 | 0.407195713 | 0.535 | 0.513 | 1 |
| PLEK | 0.090237204 | -0.263273459 | 0.826 | 0.809 | 1 |
| PLCG2 | 0.139989143 | 0.275878199 | 0.383 | 0.385 | 1 |
| S100A8 | 0.687206357 | -1.014420936 | 0.364 | 0.341 | 1 |
| IGKC | 0.701628589 | -0.425192526 | 0.35 | 0.333 | 1 |

**Supplementary** Table 6. Potential drugs for the treatment of AS were mined through the CMAP.

| **Rank** | **Score** | **Type** | **ID** | **Name** | **Description** |
| --- | --- | --- | --- | --- | --- |
| **1** | 99.75 | cp | BRD-K06792661 | narciclasine | Coflilin signalling pathway activator |
| **5** | 99.3 | cp | BRD-A47513740 | calyculin | Protein phosphatase inhibitor |
| **6** | 99.08 | cp | BRD-A26002865 | verrucarin-a | Protein synthesis inhibitor |
| **8** | 98.63 | cp | BRD-K67506692 | tyrphostin-AG-126 | ERK1 and ERK2 phosphorylation inhibitor |
| **10** | 98.45 | cp | BRD-K76674262 | homoharringtonine | Protein synthesis inhibitor |
| **13** | 98.27 | cp | BRD-U86922168 | QL-XII-47 | BTK inhibitor |
| **15** | 98.08 | cp | BRD-K80348542 | cephaeline | Protein synthesis inhibitor |
| **16** | 98.06 | cp | BRD-A25687296 | emetine | Protein synthesis inhibitor |
| **17** | 98.03 | cp | BRD-A52193669 | withaferin-a | IKK inhibitor |
| **24** | 97.36 | cp | BRD-A71459254 | cymarin | ATPase inhibitor |

**Supplementary** Table 7. The target of narciclasine.

| **Datebase** | **Target gene** |
| --- | --- |
| **Binding DB** | RIPK2 CYP3A4 |
| **PubChem** | EEF1A1 RHOA PRKAA2 TNF MAPK8 TERF1 RELA MAPK14 ACHE AKT1 ULK1 TRAF6 GORASP1 SYT1 S100A8 MTOR ANXA5 IL6 PRKAA1 VEGFA KLHL41PPP1R12B CDS2 AIMP2 AHSA1 PTGS2 VCAM1 PSMA8 ANG TEAD4 TLR4 CLC PIN4 GRHPR SPOP ICAM1 NOS2 CYP3A4 RP2 RORA CYP2D4 TRAF3IP2 IL10 CTSB PIK3CA TPM2 IRAK1 CASP8 KCNA2 ROCK2 SGSH PTX3 CYP19A1 CASP9 SPAG9 KCNJ2 PANK2 ROCK1 CASP3 IL1R1 GFER BRD4 CASP10 IRF3 RUNX1 GRIN1 CD69 DEGS1 TP53 CDH5 BMAL1 PTK2 SCN5A CD80 BECN1 TNFRSF10B IL17A CASP7 PGAM1 CD86 |
| **STITCH** | EEF1A1 WEE2 |
| **TargetNet** | TUBB2B CLK1 PTGS1 MIF ALOX15 DYRK1A CES2 HSP90AA1 CA6 BCL2A1 AHR RIPK2 PIM1 CYP2C19 CDC7 DRD1 NR3C2 GPR35 RPS6KA3 KIF11 |
| **SwissTarget** | MMP3 ADAM17 PNP TYR ADORA1 ADK ADA |

**Supplementary Table 8.** Thermodynamic parameters of VCAM-1 and ICAM-1

| Parameter | VCAM-1 | ICAM-1 |
| --- | --- | --- |
| KD | 3.5 ± 0.2 | 2.8 ± 0.3 |
| ΔG^o^ | -31.2 ± 0.8 | -32.1 ± 0.7 |
| ΔH^o^ | -22.8 ± 1.5 | -25.6 ± 1.3 |
| TΔS^o^ | 8.4 ± 0.9 | 6.5 ± 0.8 |
